# The red blood cell distribution width-to-albumin ratio and 28-day mortality in patients with severe cardiogenic shock: a retrospective cohort study based on MIMIC-IV with single-center external validation

**DOI:** 10.3389/fmed.2026.1857535

**Published:** 2026-09-30

**Authors:** Yi Ou, Xiongwei Meng, Dan Zhang, Junhong Wu, Chen Chen, Shengli Yin

**Affiliations:** 1 Department of Cardiac Surgery, Affiliated Hospital of Guizhou Medical University, Guiyang, China; 2 Guizhou Medical University, Guiyang, China; 3 Affiliated People’s Hospital of Xingyi City, Guizhou Medical University, Xingyi, China; 4 Guizhou Medical University Affiliated Guian Hospital, Guiyang, China

**Keywords:** 28-day mortality, cardiogenic shock, prognostic prediction model, red cell distribution width-to- albumin ratio (RAR), retrospective cohort study

## Abstract

**Background:**

Cardiogenic shock (CS) is a critical syndrome with a very high fatality rate, and in-hospital mortality ranges from 27% to 51%. Among the various prognostic approaches, serum biomarkers have attracted considerable attention because they are easy to measure, inexpensive, and can be monitored dynamically. The red cell distribution width-to-albumin ratio (RAR) is a novel composite marker that integrates red blood cell heterogeneity (reflecting inflammation and oxidative stress) with albumin levels (indicating nutritional and anti-inflammatory capacity). It has shown initial promise for risk stratification in critically ill patients. However, the independent prognostic value of RAR specifically in CS remains unclear. Therefore, this study aims to systematically evaluate the ability of RAR to predict 28-day mortality in these patients.

**Materials and methods:**

This study used a two-cohort retrospective design. The internal training cohort was derived from CS patients in the MIMIC-IV v2.2 database, and the external validation cohort came from the medical record system of the Affiliated Hospital of Guizhou Medical University. To explore the association between RAR and short-term poor outcomes in severe CS, we applied multivariable Cox regression, restricted cubic spline (RCS) regression, subgroup analysis, and Kaplan–Meier survival curves. To develop a simple prediction model, the internal cohort was randomly split into a training set and a test set in a 7:3 ratio. In the training set, we used five methods—Boruta algorithm, LASSO-Cox regression, random forest (RF), gradient boosting (GBDT), and support vector machine (SVM)—to select key features associated with poor CS prognosis, covering laboratory indicators, comorbidities, demographic characteristics, and vital signs. Based on the selected feature variables, we built prognostic prediction models using multivariable Cox regression in each of the three cohorts. The discriminative ability of the model was compared with that of traditional disease severity scores using receiver operating characteristic (ROC) curves, with the area under the curve (AUC) as the measure of predictive performance.

**Result:**

A total of 1,314 CS patients were included in this study. In the fully adjusted model (Model 3), both continuous RAR and RAR quartiles were independently associated with 28-day adverse outcomes. For 28-day ICU mortality, each unit increase in RAR was associated with a 13.4% higher risk (HR 1.134, 95% CI 1.076–1.197, *p* < 0.001); compared with the lowest quartile (Q1), the highest quartile (Q4) had a significantly increased risk of 51.7% (HR 1.517, 95% CI 1.123–2.049, *p* = 0.007). For 28-day in-hospital mortality, the HR for continuous RAR was 1.085 (95% CI 1.031–1.141, *p* = 0.002), and the HR for Q4 vs. Q1 was 1.576 (95% CI 1.196–2.076, *p* = 0.001). Restricted cubic spline analysis further confirmed a significant positive dose-response relationship between RAR and mortality (overall *p* < 0.05). In the external validation cohort, the predictive value of RAR for 28-day ICU mortality remained consistent and was even stronger (HR for continuous RAR: 1.359, 95% CI 1.177–1.568, *p* < 0.001). the RAR-based risk prediction model showed moderate but consistent discriminative performance, with areas under the receiver operating characteristic curve of 0.71, 0.70, and 0.75 in the training, test, and external validation sets, respectively, all of which were higher than those of traditional severity scores (all DeLong tests *p* < 0.05).

**Conclusion:**

This study demonstrated in both cohorts that RAR is an independent associated with 28-day mortality risk in CS patients. The prediction model built on RAR and five other clinical features exhibited good clinical performance. With further prospective validation, this model may become a valuable exploratory tool for risk stratification in CS patients.

## Introduction

Cardiogenic shock (CS) is a critical syndrome caused by the sudden failure of the heart's pumping function, leading to severely low cardiac output, a sharp drop in tissue perfusion, and multiple organ dysfunction. It remains a leading cause of cardiovascular death worldwide (1, 2). Despite advances in reperfusion therapy and mechanical circulatory support for acute coronary syndromes, in-hospital mortality in CS remains high, often exceeding 40%, posing a major clinical challenge (3, 4). Its pathophysiology involves not only the failing heart but also a profound systemic inflammatory response, metabolic disturbances, and immune dysregulation, which together form a “heart-to-system” vicious cycle that ultimately determines patient outcomes (5, 6). Currently, risk stratification and prognosis assessment in CS mainly rely on hemodynamic parameters (e.g., cardiac output, pulmonary capillary wedge pressure) and traditional critical care scoring systems such as SOFA and APACHE II (7–9). However, these measures are often substantially influenced by interventions such as vasopressors, mechanical ventilation, and circulatory support, which limits their stability and specificity for dynamic assessment (10, 11). Therefore, identifying a stable and objective biomarker that reflects systemic metabolic stress and the inflammatory-nutritional state in CS patients is of great value for early risk recognition and targeted intervention.

Among the many blood biomarkers, red cell distribution width (RDW) and albumin represent two core dimensions of critical illness pathophysiology. RDW is a measure of the variability in red blood cell size. An elevated RDW is closely associated with systemic inflammation, oxidative stress, poor nutrition, and disordered blood cell production, and has been recognized as an independent prognostic marker of poor outcomes in several cardiovascular diseases (12, 13). Serum albumin, on the other hand, is not only essential for maintaining plasma colloid osmotic pressure and transporting substances; it also serves as a comprehensive marker of liver synthetic function, nutritional status, and systemic inflammation (as a negative acute-phase protein). Hypoalbuminemia is strongly associated with immune dysfunction, increased infection risk, and higher mortality in critically ill patients (14, 15). However, in the complex setting of CS, using either RDW or albumin alone for prognosis has limitations: RDW is influenced by chronic disease status and inflammation, giving it relatively limited specificity, while albumin has a long half-life and responds slowly to acute changes.

The red cell distribution width-to-albumin ratio (RAR) is an emerging composite marker that integrates information on systemic inflammation/oxidative stress (from RDW) and nutritional/inflammatory status (from albumin). Theoretically, it better captures the high-risk pathophysiological state of “high inflammatory-oxidative stress with low nutritional reserve” in CS patients. Previous studies have shown that RAR has better predictive value than either component alone in critical illnesses such as heart failure, acute myocardial infarction, multiorgan failure, and atrial fibrillation (16–18). However, systematic clinical evidence on the independent association between RAR and short-term mortality specifically in the more severe and complex setting of CS is still lacking.

Therefore, this study aimed to retrospectively evaluate the association between RAR and 28-day all-cause mortality in CS patients using a large intensive care database and an external independent cohort, while adjusting for confounders with multivariable models to clarify its independent prognostic value. This may provide a new, simple, and effective tool for early clinical risk stratification and intervention decision-making.

## Materials and methods

### Data source

This study used a retrospective two-cohort design to improve the robustness and generalizability of the findings. The internal cohort was derived from the MIMIC-IV database (Medical Information Mart for Intensive Care IV, v2.2) (26), a public critical care database containing anonymized, high-quality data from approximately 100,000 ICU/emergency department patients treated at Beth Israel Deaconess Medical Center between 2008 and 2019. The establishment of this database was approved by the relevant Institutional Review Board (IRB protocol numbers: 2001P001699, 0403000206), and all patient data were anonymized to protect privacy. To further test the applicability of our results across different institutions and populations, we additionally built an external validation cohort that included CS patients admitted to the Affiliated Hospital of Guizhou Medical University from March 2022 to December 2025. Data for the external cohort were retrospectively extracted from a prospectively maintained institutional clinical database. For the external validation cohort, the diagnosis of CS was confirmed by reviewing the admission and discharge diagnoses recorded in the electronic medical records, supported by a combination of clinical symptoms, signs, laboratory tests, and imaging findings. The study protocol was approved by the Ethics Committee of the Affiliated Hospital of Guizhou Medical University.

### Study population and inclusion criteria

This study focused on patients with cardiogenic shock (CS) admitted to the intensive care unit (ICU) for the first time. Patients in the internal cohort were identified using the International Classification of Diseases, Ninth and Tenth Revisions (ICD-9/10) diagnostic codes (785.51 and R57.0), corresponding to cardiogenic shock. Inclusion criteria were: (1) age ≥18 years; (2) first ICU admission; (3) ICU stay and survival of at least 24 h; (4) availability of complete lactate and albumin test results; and (5) meeting the operational definition of cardiogenic shock consistent with the Society for Cardiovascular Angiography and Interventions (SCAI) consensus criteria — namely, either systolic blood pressure <90 mmHg in the absence of vasoactive support, or the use of vasoactive drugs (vasopressors and/or inotropes) to maintain systolic blood pressure ≥90 mmHg, accompanied by evidence of tissue hypoperfusion (e.g., serum lactate >2 mmol/L, altered mental status, cold extremities, or urine output <30 mL/h). For the external validation cohort, the diagnosis of CS was confirmed prospectively at the Affiliated Hospital of Guizhou Medical University using the same operational definition, supported by a combination of clinical symptoms, signs, laboratory tests, and imaging findings (systolic blood pressure <90 mmHg for ≥30 min or need for vasoactive drugs to maintain blood pressure, accompanied by signs of tissue hypoperfusion). The two cohorts therefore share the same operational definition of cardiogenic shock; their clinical characteristics are summarized in Supplementary Table S5. To confirm that the RAR–mortality association is not an artifact of cohort identification, a pre-specified sensitivity analysis was performed in the MIMIC-IV subset that unambiguously met both the hemodynamic and the hypoperfusion criteria (Supplementary Table S6 and Supplementary Figure S3).

### Variable extraction

Data extraction was performed using PostgreSQL (v13.7.2) and Navicat Premium (v16) by executing structured query language (SQL) commands. All laboratory measurements, including RDW and albumin, were extracted as the first recorded value within 24 h of ICU admission. These values preceded the ascertainment of clinical outcomes (28-day ICU and in-hospital mortality) and were obtained before or at the time of ICU presentation, not after clinical deterioration or therapeutic interventions. Vasopressor initiation and mechanical circulatory support were not used as criteria for selecting or excluding laboratory measurements. The extracted variables were divided into six categories: (1) demographics; (2) comorbidities; (3) vital signs; (4) disease severity scores at admission; (5) first laboratory test results within 24 h of admission; and (6) therapeutic interventions. Detailed information on these variables is provided in the baseline table (Table 1).

**Table 1:** Baseline characteristics of the internal cohort stratified by RAR quartiles.

Variable	[ALL]	Q1	Q2	Q3	Q4	*P*-value
	*N* = 1314	*N* = 328	*N* = 328	*N* = 328	*N* = 330	
RAR (RDW/Albumin), unitless	5.23 (1.61)	3.74 (0.290)	4.49 (0.205)	5.33 (0.290)	7.38 (1.64)	<0.001
Age, years	68.8 (14.6)	66.9 (15.1)	69.0 (15.1)	71.3 (13.2)	68.1 (14.8)	0.001
Gender:						<0.001
F	512 (39.0%)	100 (30.5%)	125 (38.1%)	147 (44.8%)	140 (42.4%)	
M	802 (61.0%)	228 (69.5%)	203 (61.9%)	181 (55.2%)	190 (57.6%)	
Race:						0.012
Other races	526 (40.0%)	152 (46.3%)	128 (39.0%)	111 (33.8%)	135 (40.9%)	
White	788 (60.0%)	176 (53.7%)	200 (61.0%)	217 (66.2%)	195 (59.1%)	
BMI, kg/m^2^	29.2 (7.44)	28.8 (7.54)	29.1 (7.28)	29.5 (7.25)	29.3 (7.70)	0.696
Hypertension:						0.024
No	1001 (76.2%)	236 (72.0%)	241 (73.5%)	257 (78.4%)	267 (80.9%)	
Yes	313 (23.8%)	92 (28.0%)	87 (26.5%)	71 (21.6%)	63 (19.1%)	
AKI:						0.005
No	402 (30.6%)	123 (37.5%)	105 (32.0%)	88 (26.8%)	86 (26.1%)	
Yes	912 (69.4%)	205 (62.5%)	223 (68.0%)	240 (73.2%)	244 (73.9%)	
CKD:						<0.001
No	887 (67.5%)	251 (76.5%)	226 (68.9%)	193 (58.8%)	217 (65.8%)	
Yes	427 (32.5%)	77 (23.5%)	102 (31.1%)	135 (41.2%)	113 (34.2%)	
Diabetes:						0.010
No	829 (63.1%)	223 (68.0%)	216 (65.9%)	184 (56.1%)	206 (62.4%)	
Yes	485 (36.9%)	105 (32.0%)	112 (34.1%)	144 (43.9%)	124 (37.6%)	
Hyperlipidemia:						0.408
No	720 (54.8%)	175 (53.4%)	177 (54.0%)	174 (53.0%)	194 (58.8%)	
Yes	594 (45.2%)	153 (46.6%)	151 (46.0%)	154 (47.0%)	136 (41.2%)	
HF:						0.687
No	290 (22.1%)	80 (24.4%)	70 (21.3%)	68 (20.7%)	72 (21.8%)	
Yes	1024 (77.9%)	248 (75.6%)	258 (78.7%)	260 (79.3%)	258 (78.2%)	
MI:						<0.001
No	855 (65.1%)	181 (55.2%)	204 (62.2%)	216 (65.9%)	254 (77.0%)	
Yes	459 (34.9%)	147 (44.8%)	124 (37.8%)	112 (34.1%)	76 (23.0%)	
IHD:						<0.001
No	416 (31.7%)	90 (27.4%)	96 (29.3%)	96 (29.3%)	134 (40.6%)	
Yes	898 (68.3%)	238 (72.6%)	232 (70.7%)	232 (70.7%)	196 (59.4%)	
COPD:						0.026
No	1074 (81.7%)	280 (85.4%)	276 (84.1%)	253 (77.1%)	265 (80.3%)	
Yes	240 (18.3%)	48 (14.6%)	52 (15.9%)	75 (22.9%)	65 (19.7%)	
HR, beats/min	92.5 (22.7)	89.8 (21.3)	91.7 (22.9)	91.9 (21.6)	96.6 (24.3)	0.001
NBPS, mmHg	110 (22.2)	112 (23.1)	112 (22.2)	112 (21.6)	106 (21.4)	<0.001
NBPD, mmHg	66.7 (18.6)	69.2 (18.2)	66.5 (19.7)	66.2 (17.6)	65.0 (18.6)	0.028
RR, insp/min	20.7 (6.69)	20.2 (6.15)	20.7 (6.73)	21.1 (6.83)	20.8 (7.01)	0.327
SpO₂, %	95.9 (5.46)	96.3 (4.62)	95.7 (5.95)	95.9 (5.60)	95.9 (5.58)	0.486
Temperature,°F	97.6 (5.68)	97.7 (4.96)	97.4 (7.46)	97.7 (4.94)	97.6 (4.95)	0.888
SOFA, Score	7.70 (3.62)	6.63 (3.32)	7.62 (3.48)	8.11 (3.65)	8.43 (3.78)	<0.001
APS III, Score	59.3 (21.9)	50.9 (19.7)	58.5 (21.6)	61.6 (22.1)	66.0 (21.4)	<0.001
SAPS II, Score	46.3 (14.5)	41.3 (13.6)	46.0 (14.6)	48.4 (14.3)	49.5 (14.1)	<0.001
OASIS, Score	36.2 (8.94)	34.2 (8.32)	36.1 (9.28)	37.2 (9.07)	37.1 (8.79)	<0.001
CCI, Score	6.28 (2.82)	5.48 (2.48)	6.05 (2.71)	6.92 (2.75)	6.67 (3.10)	<0.001
APACHE II, Score	22.9 (7.50)	20.4 (7.42)	22.5 (7.37)	24.0 (7.22)	24.5 (7.31)	<0.001
HCT, %	34.4 (7.28)	38.0 (6.60)	34.7 (6.80)	33.1 (6.80)	31.8 (7.45)	<0.001
Hb, g/dL	11.1 (2.46)	12.5 (2.27)	11.3 (2.28)	10.6 (2.26)	10.1 (2.35)	<0.001
PLT, K/*μ*L	207 (102)	215 (73.4)	208 (99.5)	206 (99.7)	199 (129)	0.289
RDW, %	15.4 (2.54)	13.7 (1.06)	14.6 (1.33)	15.7 (1.96)	17.7 (3.16)	<0.001
RBC, M/μL	3.75 (0.838)	4.10 (0.745)	3.72 (0.746)	3.65 (0.821)	3.55 (0.924)	<0.001
WBC, K/μL	14.5 (8.04)	14.3 (5.79)	14.6 (7.69)	13.9 (7.03)	15.1 (10.7)	0.264
ALB, g/dL	3.08 (0.586)	3.67 (0.341)	3.25 (0.308)	2.95 (0.369)	2.46 (0.495)	<0.001
AG, mEq/L	16.7 (5.16)	16.0 (4.58)	16.7 (4.92)	17.3 (5.45)	16.8 (5.56)	0.014
TCa, mg/dL	8.34 (0.912)	8.57 (0.760)	8.34 (0.806)	8.36 (0.925)	8.10 (1.07)	<0.001
Cl, mEq/L	102 (7.35)	102 (6.16)	102 (7.58)	101 (7.78)	102 (7.74)	0.191
GLU, mg/dL	177 (89.4)	184 (86.9)	177 (86.4)	181 (97.1)	166 (86.1)	0.065
K, mEq/L	4.46 (0.861)	4.53 (0.881)	4.44 (0.794)	4.46 (0.857)	4.40 (0.908)	0.283
Na, mEq/L	137 (5.91)	137 (5.09)	137 (5.51)	137 (6.22)	137 (6.70)	0.536
Mg, mg/dL	2.17 (0.575)	2.15 (0.594)	2.18 (0.526)	2.18 (0.532)	2.14 (0.640)	0.727
TCO2, mEq/L	22.8 (5.51)	22.5 (4.56)	22.9 (5.43)	23.0 (6.21)	22.8 (5.71)	0.682
Lac, mmol/L	3.11 (2.58)	2.57 (1.81)	3.02 (2.45)	3.44 (3.02)	3.41 (2.80)	<0.001
PCO₂, mmHg	42.4 (11.9)	41.7 (10.6)	42.4 (12.0)	42.8 (13.3)	42.6 (11.5)	0.683
pH, pH units	7.33 (0.107)	7.34 (0.101)	7.33 (0.107)	7.33 (0.115)	7.33 (0.107)	0.861
PO₂, mmHg	131 (116)	131 (118)	140 (120)	130 (117)	122 (110)	0.249
INR, Ratio	1.87 (1.39)	1.58 (0.898)	1.87 (1.28)	2.05 (1.82)	1.97 (1.34)	<0.001
PT, seconds	20.2 (13.8)	17.1 (9.58)	20.4 (13.8)	22.0 (18.0)	21.1 (12.2)	<0.001
APTT, seconds	53.5 (37.7)	55.6 (38.9)	54.6 (38.9)	51.8 (36.1)	51.9 (36.8)	0.460
ALT, IU/L	335 (922)	260 (714)	349 (800)	411 (1263)	321 (810)	0.205
AST, IU/L	535 (1606)	441 (1218)	515 (1252)	689 (2318)	496 (1374)	0.223
TB, mg/dL	1.26 (1.71)	1.01 (1.09)	1.13 (1.21)	1.28 (1.58)	1.61 (2.52)	<0.001
CRE, mg/dL	1.97 (1.64)	1.53 (0.969)	1.94 (1.57)	2.18 (1.70)	2.22 (2.05)	<0.001
BUN, mg/dL	37.9 (25.4)	30.1 (21.0)	37.0 (25.1)	42.1 (26.4)	42.2 (26.7)	<0.001
CK-MB, ng/mL	53.2 (94.6)	77.7 (122)	55.0 (95.0)	37.6 (69.8)	42.5 (78.4)	<0.001
LDH, IU/L	903 (1551)	752 (1013)	861 (1236)	985 (1833)	1014 (1918)	0.114
cTnI, ng/mL	1.97 (4.21)	2.06 (4.04)	2.22 (3.43)	1.73 (5.39)	1.81 (3.52)	0.834
Phosphate, mg/dL	4.52 (1.74)	4.21 (1.43)	4.45 (1.68)	4.68 (1.82)	4.76 (1.94)	<0.001
Ventilation duration, hours	113 (128)	106 (120)	105 (111)	126 (137)	117 (140)	0.113
Ventilation:						0.660
No	116 (8.83%)	26 (7.93%)	26 (7.93%)	30 (9.15%)	34 (10.3%)	
Yes	1198 (91.2%)	302 (92.1%)	302 (92.1%)	298 (90.9%)	296 (89.7%)	
CRRT:						<0.001
No	1038 (79.0%)	290 (88.4%)	265 (80.8%)	251 (76.5%)	232 (70.3%)	
Yes	276 (21.0%)	38 (11.6%)	63 (19.2%)	77 (23.5%)	98 (29.7%)	
CRRT duration, days	1.55 (5.02)	0.93 (3.42)	1.45 (6.74)	1.65 (4.16)	2.16 (5.07)	0.017
Sympathomimetic/inotrope:						0.254
No	237 (18.0%)	63 (19.2%)	63 (19.2%)	47 (14.3%)	64 (19.4%)	
Yes	1077 (82.0%)	265 (80.8%)	265 (80.8%)	281 (85.7%)	266 (80.6%)	
Vasopressor:						0.005
No	22 (1.67%)	11 (3.35%)	8 (2.44%)	1 (0.30%)	2 (0.61%)	
Yes	1292 (98.3%)	317 (96.6%)	320 (97.6%)	327 (99.7%)	328 (99.4%)	
Glucocorticoid:						<0.001
No	963 (73.3%)	252 (76.8%)	256 (78.0%)	243 (74.1%)	212 (64.2%)	
Yes	351 (26.7%)	76 (23.2%)	72 (22.0%)	85 (25.9%)	118 (35.8%)	
Hospital length of stay, days	17.1 (16.6)	14.9 (13.1)	17.5 (21.2)	17.9 (16.2)	18.3 (14.8)	0.039
ICU length of stay, days	8.57 (8.54)	7.99 (7.53)	7.98 (7.81)	9.18 (9.17)	9.10 (9.43)	0.109
28-day survival time, days	20.9 (10.4)	23.4 (9.02)	22.3 (9.83)	20.0 (10.8)	17.9 (11.2)	<0.001
In-hospital mortality:						<0.001
No	824 (62.7%)	250 (76.2%)	234 (71.3%)	189 (57.6%)	151 (45.8%)	
Yes	490 (37.3%)	78 (23.8%)	94 (28.7%)	139 (42.4%)	179 (54.2%)	
28-day ICU mortality:						<0.001
No	870 (66.2%)	255 (77.7%)	241 (73.5%)	200 (61.0%)	174 (52.7%)	
Yes	444 (33.8%)	73 (22.3%)	87 (26.5%)	128 (39.0%)	156 (47.3%)	

Data are presented as mean ± standard deviation or *n* (%).

RAR, Red Cell Distribution Width-to-Albumin Ratio; BMI, body mass index; AKI, acute kidney injury; CKD, chronic kidney disease; DM, diabetes mellitus; HLD, Hyperlipidemia; HF, heart failure; MI, myocardial infarction; IHD, ischemic heart disease; COPD, chronic obstructive pulmonary disease; HR, heart rate; NBPS, non-invasive blood pressure—Systolic; NBPD, non-invasive blood pressure—diastolic; RR, respiratory rate; SpO₂, peripheral oxygen saturation; Fahrenheit, temperature; SOFA, Sequential Organ Failure Assessment; APS III, Acute Physiology Score III; SAPS II, Simplified Acute Physiology Score II; OASIS, Oxford Acute Severity of Illness Score; CCI, Charlson Comorbidity Index; APACHE II, Acute Physiology and Chronic Health Evaluation II; HCT, hematocrit; Hb, hemoglobin; PLT, platelet count; RDW, red cell distribution width; RBC, red blood cell count; WBC, white blood cell count; ALB, albumin; AG, anion gap; TCa, total calcium; Cl, Chloride; Glu, Glucose; K, Potassium; Na, Sodium; Mg, magnesium; TCO₂, Total Carbon Dioxide; Lac, Lactate; PCO₂, Partial Pressure of Carbon Dioxide; pH, Potential of Hydrogen; PO₂, Partial Pressure of Oxygen; INR, International Normalized Ratio; PT, Prothrombin Time; APTT, Activated Partial Thromboplastin Time; ALT, Alanine Aminotransferase; AST, Aspartate Aminotransferase; TB, Total Bilirubin; CRE, Creatinine; BUN, Blood Urea Nitrogen; CK-MB, Creatine Kinase-MB; LDH, Lactate Dehydrogenase; cTnI, Cardiac Troponin I; Phosphate, Ventilation Hours, Ventilation; CRRT, continuous renal replacement therapy; CRRT Days; sa, Vasopressor Administration; satotalval, total vasopressor dose; VP, vasopressor type; vptotalval, total VP dose; GC, glucocorticoid use; gctotalval, total glucocorticoid Dose; hosp_day, hospital length of stay; icu_day, ICU length of stay.

### Definition of exposure and outcomes

The red blood cell distribution width-to-albumin ratio (RAR) was calculated as RDW (%) divided by serum albumin (g/dL). Both variables were taken from the first measurement obtained within 24 h of intensive care unit admission. The resulting ratio is dimensionless and is reported as a unitless value.

### Subgroup analysis

Subgroup analyses were performed to further evaluate the consistency of the association between RAR and outcome risk across different populations. Based on predefined criteria, the analyses included age (≤65 vs. >65 years), sex, race, BMI (<30 vs. ≥30 kg/m^2^), and a range of comorbidity statuses, including hypertension, acute kidney injury (AKI), chronic kidney disease (CKD), diabetes mellitus (DM), hyperlipidemia (HLD), heart failure (HF), ischemic heart disease (IHD), myocardial infarction (MI), and chronic obstructive pulmonary disease (COPD). All subgroup analyses were conducted using Cox proportional hazards models, and the results were visualized as forest plots.

### Analysis of the association between RAR and 28-day ICU mortality in CS

Multivariable Cox regression models were used to evaluate the association between RAR and 28-day ICU mortality in CS patients. To control for potential confounders, three stepwise adjusted models were constructed: Model 1 was unadjusted; Model 2 adjusted for demographic characteristics (age, sex, race, weight) and major comorbidities; Model 3 further included clinical variables that differed significantly between survivors and non-survivors, such as disease severity scores, key laboratory markers, and therapeutic interventions (Supplementary Table S1-S3). To ensure model stability, we performed multicollinearity diagnostics on all variables in Model 3, calculated variance inflation factors (VIF), and removed variables with VIF > 5 (Supplementary Table S4). Finally, to explore possible non-linear relationships between RAR and the outcome, restricted cubic splines (RCS) were fitted with knots placed at the 5th, 35th, 65th, and 95th percentiles.

### Feature selection, risk prediction modeling, and validation

To develop a parsimonious yet robust prediction model, we performed feature selection exclusively in the training set (70% of the internal cohort). We employed an ensemble of five complementary feature selection methods: Boruta, LASSO-Cox, random forest (RF), gradient boosting decision trees (GBDT), and support vector machine (SVM). This multi-algorithm strategy was adopted to leverage the complementary strengths of different methodological paradigms—linear (LASSO-Cox), non-linear tree-based (RF, GBDT), and margin-based (SVM)—thereby enhancing the robustness and generalizability of the selected predictors. All tuning procedures were strictly confined to the training data. The hyperparameters for each method were pre-specified as follows: Boruta: confidence level = 0.01, maximum runs of importance source = 100, with Bonferroni correction for multiple testing; LASSO-Cox: 100 lambda values, lambda selection based on the minimum mean cross-validated error (lambda.min), 10-fold cross-validation, with deviance as the loss criterion; Random Forest: random seed = 1, number of trees = 100, maximum depth = 3, minimum samples for split = 2, minimum samples per leaf node = 1; Gradient Boosting Decision Trees: random seed = 1, loss function = squared error, learning rate = 0.1, number of boosting iterations = 100, split quality criterion = friedman_mse, minimum samples for split = 2, minimum samples per leaf node = 1; Support Vector Machine: random seed = 1, regularization parameter C = 1.0, kernel function = radial basis function (rbf). Specifically, we considered 18 core candidate variables (laboratory indicators, admission vital signs, demographic characteristics, and comorbidities) that were available in the training set. Boruta algorithm identified 12 key predictors; LASSO regression identified 17; RF identified 9; GBDT identified 8; and SVM identified 7 (Figures 4A–D). To obtain the most robust set of prognostic variables, we then used a Venn diagram to identify the intersection of variables selected by all five methods (Figure 4E). This conservative “consensus” strategy was chosen to prioritize reproducibility and robustness over maximizing in-sample predictive performance, as variables identified by all five methods are less likely to represent algorithm-specific artifacts and more likely to capture biologically meaningful signals. This approach reduces the risk of overfitting and enhances the generalizability of the final model to independent cohorts. This conservative “consensus” approach ensures that the final variables are not artifacts of any single algorithm's bias. Six independent predictors were finally confirmed: RAR, age, BUN, creatinine, glucose, and phosphate. We then applied multivariable Cox regression to these six core variables in the training set to construct the final risk prediction model (Table 4). The regression coefficients (*β*) of the model represent the change in the log-hazard ratio for each one-unit increase in a given variable, after adjusting for other factors. Based on this, a risk score for each patient was calculated using the linear combination formula: risk score = *Σ*(*β*ᵢ × variable). The 28-day predicted probability of mortality for each patient was then derived from the Cox model as 1−S₀(28)^exp(LP), where S₀(28) is the baseline survival probability at 28 days estimated by the Kaplan–Meier method, and LP is the linear predictor (risk score). Calibration curves were generated by grouping patients into deciles of predicted risk and plotting the mean predicted probability against the observed proportion of events within each decile, with 95% confidence intervals. Brier scores were calculated as the mean squared difference between the observed outcome (0 for survival, 1 for death at 28 days) and the predicted probability. A prognostic prediction model was then constructed and visualized as a nomogram. The discriminative ability of the model was assessed using conventional binary receiver operating characteristic (ROC) curves, where the outcome was defined as 28-day mortality status (patients alive at 28 days were classified as survivors), and the area under the curve (AUC) was calculated, with comparisons made using DeLong's test. Model performance was subsequently evaluated in the internal test set (30% of the internal cohort) and the external validation cohort, neither of which was used during feature selection or model development. Finally, to evaluate the stability and generalizability of the constructed model, we independently validated its predictive performance in the external validation cohort.

### Statistical analysis

All statistical analyses were performed using R software (RStudio, version 4.2.2) and DecisionLinnc version 1.0 (https://www.statsape.com/). Continuous variables were presented as mean ± standard deviation (SD) or median (interquartile range, IQR) as appropriate, and categorical variables as frequencies and percentages. Between-group comparisons were performed using t-test, ANOVA, Mann–Whitney U test, or Kruskal–Wallis test for continuous variables, and Pearson's chi-square test or Fisher's exact test for categorical variables. Variables with >30% missing values were excluded; for variables with <30% missingness, multiple imputation was performed using the R package mice (Multivariate Imputation by Chained Equations) with 5 imputed datasets, using predictive mean matching for continuous variables and logistic regression for binary variables. The imputation model included all variables used in the subsequent analyses. For Cox proportional hazards regression models, the proportional hazards assumption was tested using Schoenfeld residual-based tests; no violations were detected (global *p* > 0.05 for all models). Multivariable Cox regression was used to evaluate the association between RAR and 28-day mortality, with results reported as hazard ratios (HRs) and 95% confidence intervals (CIs). Restricted cubic splines (RCS) were fitted with knots at the 5th, 35th, 65th, and 95th percentiles of the RAR distribution to explore potential non-linear relationships. Kaplan–Meier survival curves with log-rank tests were used to compare survival between high- and low-RAR groups (dichotomized at the median). Subgroup analyses were pre-specified based on clinically relevant variables and were conducted using Cox models with interaction terms. For model development, the internal cohort was randomly split into a training set (70%) and an internal test set (30%). Feature selection was performed using ensemble machine learning methods (Boruta, LASSO-Cox, random forest, support vector machine, and gradient boosting decision trees), followed by multivariable Cox regression. Model discrimination was assessed using receiver operating characteristic (ROC) curves and area under the curve (AUC), with comparisons made using DeLong's test. Model calibration was evaluated using the Hosmer-Lemeshow goodness-of-fit test and calibration plots. Decision curve analysis (DCA) was performed to assess clinical net benefit across threshold probabilities of 0.2–0.8. Internal validation was performed on the 30% test set, and external validation was performed on the independent cohort. A two-sided *p*-value < 0.05 was considered statistically significant.

## Result

### Baseline characteristics of study participants

A total of 1,314 CS patients were included in this study and divided into four groups (Q1 to Q4) according to RAR quartiles, with 328–330 patients per group (Table 1). The internal cohort had a mean age of 68.8 (14.6) years and 802 (61.0%) were male. Median RAR was 5.23 (IQR 1.61), with quartile means of 3.74 (0.29), 4.49 (0.21), 5.33 (0.29), and 7.38 (1.64) for Q1–Q4, respectively. As RAR increased, patients showed progressively worse clinical profiles. Baseline characteristics stratified by ICU/in-hospital death are shown in Supplementary Tables S1, S2. Significant differences were observed across groups in age, sex, comorbidities, and laboratory parameters (all *p* < 0.05). Patients in higher RAR quartiles were older (Q4: 68.1 ± 14.8 vs. Q1: 66.9 ± 15.1 years, *p* = 0.001). Comorbidities showed a dose-dependent pattern: the prevalence of AKI (Q4: 73.9% vs. Q1: 62.5%), CKD (34.2% vs. 23.5%), DM (37.6% vs. 32.0%), and COPD (19.7% vs. 14.6%) in Q4 was significantly higher than in Q1. Disease severity scores increased with rising RAR: Q4 had significantly higher APACHE II (24.5 vs. 20.4), SOFA (8.43 vs. 6.63), SAPS II (49.5 vs. 41.3), and APS III (66.0 vs. 50.9) scores compared with Q1 (all *p* < 0.001). Laboratory findings indicated that the high-RAR group had lower hemoglobin (10.1 vs. 12.5 g/dL) and albumin (2.46 vs. 3.67 g/dL), and higher RDW (17.7% vs. 13.7%), lactate (3.41 vs. 2.57 mmol/L), creatinine (2.22 vs. 1.53 mg/dL), and BUN (42.2 vs. 30.1 mg/dL) (all *p* < 0.001). In terms of treatment, the use of CRRT increased from 11.6% in Q1 to 29.7% in Q4 (*p* < 0.001), and vasopressor use remained high across all groups (96.6% → 99.4%, *p* = 0.005). Clinical outcomes demonstrated a robust monotonic gradient: 28-day in-hospital mortality rose from 78/328 (23.8%) in Q1 to 94/328 (28.7%) in Q2, 139/328 (42.4%) in Q3, and 179/330 (54.2%) in Q4 (*p* < 0.001); 28-day ICU mortality followed a parallel pattern: 73/328 (22.3%) → 87/328 (26.5%) → 128/328 (39.0%) → 156/330 (47.3%) (*p* < 0.001). Thus, higher RAR is closely associated with worse clinical status, greater comorbidity burden, more severe organ dysfunction, and markedly increased short-term mortality in CS patients, suggesting that RAR has good potential for risk stratification.

### Comparison of baseline characteristics between the two cohorts

To facilitate a direct comparison between the two cohorts and to assess the clinical comparability of the external validation population, we summarized the baseline characteristics of both cohorts in Supplementary Table S7. Overall, the two cohorts were highly comparable across most demographic, clinical, and laboratory parameters. The mean age was 68.8 ± 14.6 years in the internal cohort and 70.2 ± 13.7 years in the external cohort (*p* = 0.141), with a similar male predominance (61.0% vs. 61.2%, *p* = 0.952). The prevalence of most comorbidities, including hypertension (23.8% vs. 25.7%, *p* = 0.497), AKI (69.4% vs. 64.9%, *p* = 0.142), CKD (32.5% vs. 35.1%, *p* = 0.395), HF (77.9% vs. 79.7%, *p* = 0.507), MI (34.9% vs. 34.4%, *p* = 0.875), and COPD (18.3% vs. 18.5%, *p* = 0.934), did not differ significantly between cohorts. Disease severity scores, including SOFA (7.70 ± 3.62 vs. 7.64 ± 3.64, *p* = 0.802), APACHE II (22.9 ± 7.50 vs. 22.9 ± 7.38, *p* = 0.965), SAPS II (46.3 ± 14.5 vs. 46.3 ± 14.8, *p* = 0.985), and APS III (59.3 ± 21.9 vs. 59.4 ± 21.5, *p* = 0.938), were nearly identical between the two cohorts. Key laboratory parameters, including RDW (15.4 ± 2.54% vs. 15.1 ± 2.38%, *p* = 0.069), albumin (3.08 ± 0.59 vs. 3.04 ± 0.52 g/dL, *p* = 0.285), RAR (5.23 ± 1.61 vs. 5.04 ± 1.45, *p* = 0.064), lactate (3.11 ± 2.58 vs. 3.15 ± 2.41 mmol/L, *p* = 0.812), creatinine (1.97 ± 1.64 vs. 1.95 ± 1.43 mg/dL, *p* = 0.846), and BUN (37.9 ± 25.4 vs. 37.8 ± 25.2 mg/dL, *p* = 0.950), also demonstrated remarkable consistency. Vasopressor use (98.3% vs. 96.7%, *p* = 0.092) and mechanical ventilation (91.2% vs. 91.7%, *p* = 0.796) were similarly high in both cohorts. Although CRRT use was higher in the internal cohort (21.0% vs. 13.4%, *p* = 0.003), likely reflecting institutional practice variation rather than differences in baseline severity, the 28-day ICU mortality rates were strikingly similar (33.8% vs. 32.2%, *p* = 0.618). These findings collectively demonstrate that the external validation cohort is generally comparable to the internal MIMIC-IV cohort, supporting the validity of using it for independent model validation.

### The relationship between RAR and CS patients 28-day ICU and in-hospital mortality

The association between RAR and 28-day mortality was assessed using Cox proportional hazards regression models, as shown in Table 2. For 28-day ICU mortality: in the fully adjusted model (Model 3), each unit increase in continuous RAR was associated with a 13.4% higher risk of ICU death (HR 1.134, 95% CI 1.076–1.197, *p* < 0.001). Using the lowest RAR quartile (Q1) as the reference, Q2 and Q3 did not show statistical significance (HR 0.880, *p* = 0.433; HR 1.206, *p* = 0.228), whereas the Q4 group had a significantly increased risk of 51.7% (HR 1.517, 95% CI 1.123–2.049, *p* = 0.007). Of note, in the unadjusted and partially adjusted models (Model 1 and Model 2), both Q3 and Q4 showed significantly elevated risks, suggesting that confounding factors partly explained the effect observed in Q3. For 28-day in-hospital mortality: in Model 3, each unit increase in continuous RAR was associated with an 8.5% higher risk of in-hospital death (HR 1.085, 95% CI 1.031–1.141, *p* = 0.002). Compared with Q1, Q2 (HR 0.905, *p* = 0.523) and Q3 (HR 1.184, *p* = 0.251) were not statistically significant, while Q4 showed a significantly increased risk of 57.6% (HR 1.576, 95% CI 1.196–2.076, *p* = 0.001).

**Table 2:** The relationship between RAR score and 28-Day ICU and in-hospital mortality.

Characteristic	Model1			Model2			Model3		
	HR^1^	95% CI^1^	*p*-value	HR^1^	95% CI^1^	*p*-value	HR^1^	95% CI^1^	*p*-value
28-day ICU mortality									
Continuous RAR	1.240	1.186, 1.296	<0.001	1.230	1.173, 1.289	<0.001	1.134	1.076, 1.197	<0.001
RAR group									
Q1	Ref	Ref	Ref	Ref	Ref	Ref	Ref	Ref	Ref
Q2	1.238	0.907, 1.689	0.179	1.135	0.831,1.552	0.426	0.880	0.639, 1.211	0.433
Q3	1.328	0.995, 1.773	0.054	1.659	1.237, 2.225	0.001	1.206	0.889, 1.636	0.228
Q4	2.531	1.916, 3.342	<0.001	2.308	1.741, 3.060	<0.001	1.517	1.123, 2.049	0.007
28-day in-hospital mortality									
Continuous RAR	1.154	1.107, 1.204	<0.001	1.159	1.108 1.212	<0.001	1.085	1.031, 1.141	0.002
RAR group									
Q1	Ref	Ref	Ref	Ref	Ref	Ref	Ref	Ref	Ref
Q2	1.083	0.802, 1.462	0.603	1.054	0.799 1.425	0.733	0.905	0.667, 1.229	0.523
Q3	1.538	1.165, 2.030	0.002	1.392	1.049, 1.846	0.022	1.184	0.888, 1.579	0.251
Q4	1.912	1.465, 3.496	<0.001	1.831	1.399, 2.397	<0.001	1.576	1.196, 2.076	0.001

CI, confidence interval; HR, hazard ratio. Model 1: unadjusted; Model 2: Adjusted for demographic characteristics (age, gender, race, and BMI); baseline comorbidities and treatment interventions. Model 3: Further adjusted for variables that showed significant differences between survivors and non survivors (laboratory tests, vital sign indicators, disease severity score at admission, and treatment interventions).

### Linear relationship between RAR and 28-day ICU and in-hospital mortality in CS patients

RCS analysis showed that after adjusting for all confounding factors (Model 3), RAR was significantly and positively associated with both 28-day ICU mortality (Figure 1A) and 28-day in-hospital mortality (Figure 1B) (overall *p* < 0.001). The non-linearity tests indicated that the relationships between RAR and both outcomes did not deviate from linearity (ICU mortality: p for nonlinea*r* = 0.496; in-hospital mortality: p for nonlinea*r* = 0.194), suggesting that the risk of death increases linearly with each unit increase in RAR. Kaplan–Meier survival analysis divided patients into high-RAR and low-RAR groups based on the median RAR value. For 28-day ICU mortality (Figure 1C), the high-RAR group had a 103% higher risk of death compared with the low-RAR group (HR 2.03, 95% CI 1.68–2.46, *p* < 0.001). For 28-day in-hospital mortality (Figure 1D), the high-RAR group had a 116% higher risk (HR 2.16, 95% CI 1.79–2.61, *p* < 0.001). The log-rank test showed that the survival curves of the two groups separated early and the difference persisted over time.

**Figure 1:**
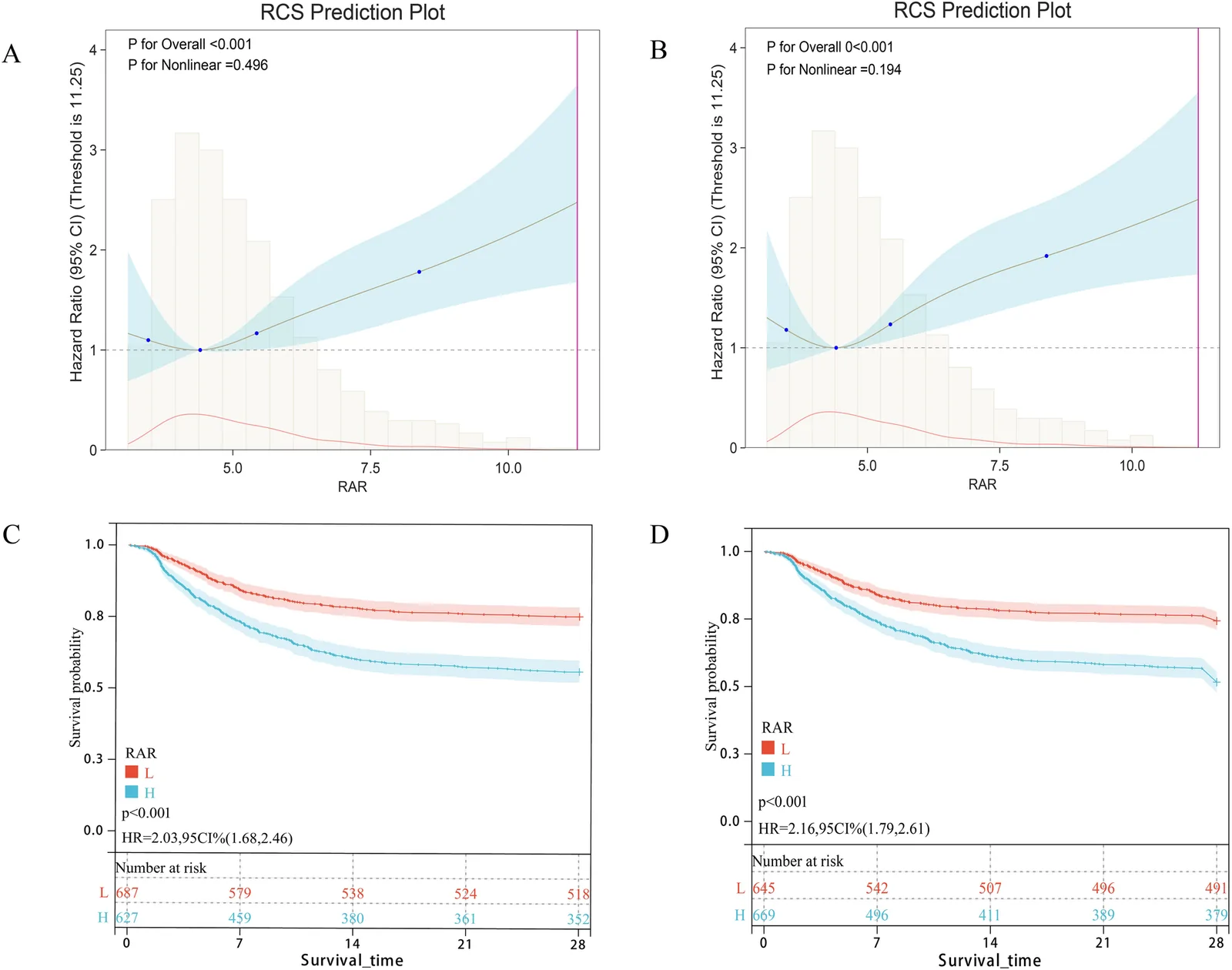
Association of the Red cell distribution width-to-albumin ratio (RAR) with 28-Day ICU and in-hospital mortality in cardiogenic shock. **(A,B)** Restricted cubic spline plots showing the dose-response relationship between RAR (continuous) and the risk of 28-day ICU mortality **(A)** and 28-day in-hospital mortality **(B)** Solid lines represent adjusted hazard ratios (HRs); shaded areas denote 95% confidence intervals (CIs). The median RAR serves as the reference (HR = 1). Overall association and non-linearity p-values are provided. **(C,D)** Kaplan–Meier curves comparing cumulative survival probabilities between high-RAR and low-RAR groups for 28-day ICU survival **(C)** and 28-day in-hospital survival **(D)** HRs with 95% CIs for high vs. low RAR are displayed, with p-values from the log-rank test.

### The incremental effect of RAR

As shown in Supplementary Figure S1, the predictive performance of RAR combined with traditional disease severity scores for 28 day ICU mortality was evaluated in the internal cohort. The results showed that adding RAR to each scoring system improved the AUC to varying degrees. Specifically: the AUC of the SOFA score increased from 0.59 to 0.65; the APS III score increased from 0.68 to 0.70; the CCI score increased from 0.62 to 0.67; the OASIS score increased from 0.62 to 0.67; the SAPS II score increased from 0.68 to 0.68–0.70; and the APACHE II score increased from 0.65 to 0.68. Among these, APS III combined with RAR achieved the best predictive performance (AUC 0.70–0.72). These findings indicate that RAR can provide additional prognostic information to traditional critical care scoring systems and improve risk stratification for 28 day ICU mortality in CS patients.

### Subgroup analysis

To assess the consistency of the association between RAR and 28-day ICU mortality across different subgroups, we performed subgroup analyses. As shown in Figure 2, after stratification by age, sex, race, BMI, hypertension, AKI, CKD, HLD, DM, MI, IHD, and COPD, RAR was significantly and positively associated with mortality risk in all subgroups (all HR >1, *p* < 0.05, except for the HF subgroup), and all interaction p-values were >0.05, indicating that these variables did not significantly modify the prognostic effect of RAR. Of note, heart failure (HF) status showed a significant interaction effect (p for interaction = 0.006): among CS patients with HF, RAR was significantly associated with mortality risk (HR 1.17, 95% CI 1.10–1.24, *p* < 0.001), whereas among patients without HF, there was no statistically significant association (HR 1.01, 95% CI 0.91–1.12, *p* = 0.815). In summary, the predictive value of RAR for 28-day mortality in CS patients remained robust across different age, sex, and most comorbidity subgroups, but its effect may be modified by the presence of heart failure.

**Figure 2:**
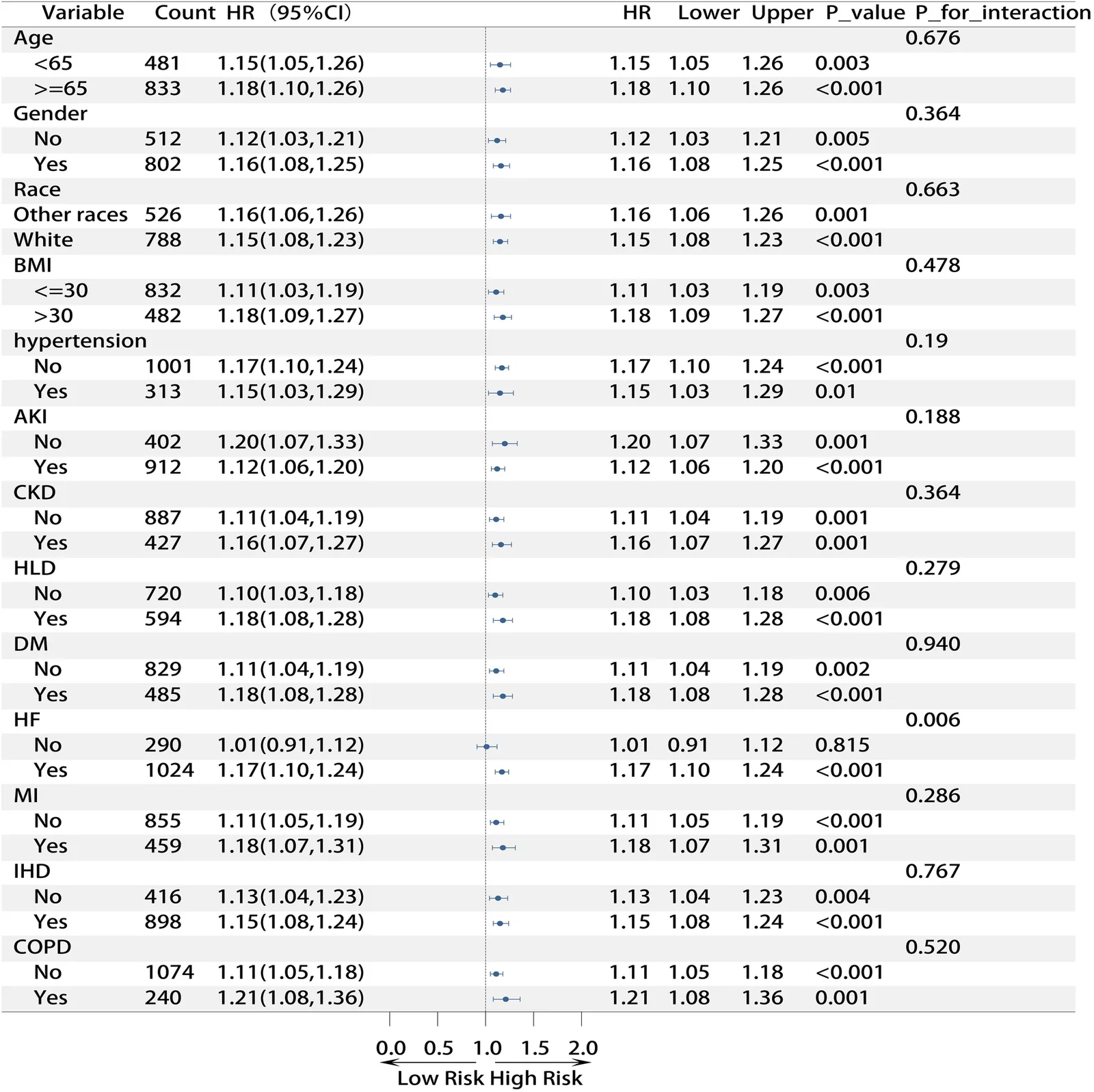
Subgroup analysis of the association between RAR and 28-Day ICU mortality. Forest plot showing hazard ratios (HRs) for 28-day ICU mortality per unit increase in RAR across predefined subgroups. Subgroups were defined by age, sex, race, BMI, and comorbidities (hypertension, AKI, CKD, DM, HLD, MI, COPD). For each subgroup, the horizontal line indicates the 95% confidence interval (CI), *P*-values for the association within each subgroup and *P*-values for interaction are presented.

### External cohort validation

According to the analysis results from the external validation cohort (Affiliated Hospital of Guizhou Medical University, March 2022 to December 2025, 276 CS patients with a 28-day ICU mortality rate of 32.25%) (Table 3; Figure 3), the association between RAR and short-term mortality risk was further validated. In the multivariable Cox regression model adjusted for all confounders (Model 3), each unit increase in continuous RAR was associated with a 35.9% higher risk of 28-day ICU mortality (HR = 1.359, 95% CI: 1.177–1.568, *P* < 0.001); using the lowest RAR quartile (Q1) as the reference, the Q4 group had a 137.3% increased risk (HR = 2.373, 95% CI: 1.113–5.061, *P* = 0.025). Restricted cubic spline (RCS) analysis showed a significant positive association between RAR and 28-day mortality risk (overall *P* = 0.003) with no evidence of non-linearity (non-linear *P* = 0.461) (Figure 3A). Kaplan–Meier survival curves further confirmed that the 28-day cumulative survival rate was significantly lower in the high-RAR group than in the low-RAR group (HR = 1.89, 95% CI: 1.22–2.94, log-rank *P* = 0.004) (Figure 3B). These findings replicated those from the internal cohort in an external independent cohort, supporting the robustness of RAR as a predictor of short-term mortality risk in CS patients.

**Table 3:** The relationship between RAR score and external validation cohort 28 day-ICU mortality.

Characteristic	Model1			Model2			Model3		
	HR^1^	95% CI^1^	*p*-value	HR^1^	95% CI^1^	*p*-value	HR^1^	95% CI^1^	*p*-value
**Continuous RAR**	1.371	1.227, 1.523	<0.001	1.404	1.236, 1.595	<0.001	1.359	1.177, 1.568	<0.001
RAR group									
Q1	Ref	Ref	Ref	Ref	Ref	Ref	Ref	Ref	Ref
Q2	1.691	0.805, 3.553	0.166	1.569	0.744, 3.309	0.236	1.161	0.528, 2.553	0.711
Q3	2.660	1.314, 5.385	0.199	2.282	1.114, 4.673	0.024	1.592	0.735, 3.448	0.238
Q4	3.647	1.843, 7.218	<0.001	3.193	1.579, 6.459	0.001	2.373	1.113, 5.061	0.025

CI, confidence interval; HR, hazard ratio. Model 1: unadjusted; Model 2: Adjusted for demographic characteristics (age, gender, race, and BMI); baseline comorbidities and treatment interventions. Model 3: Further adjusted for variables that showed significant differences between survivors and non survivors (laboratory tests, vital sign indicators, disease severity score at admission, and treatment interventions).

**Figure 3:**
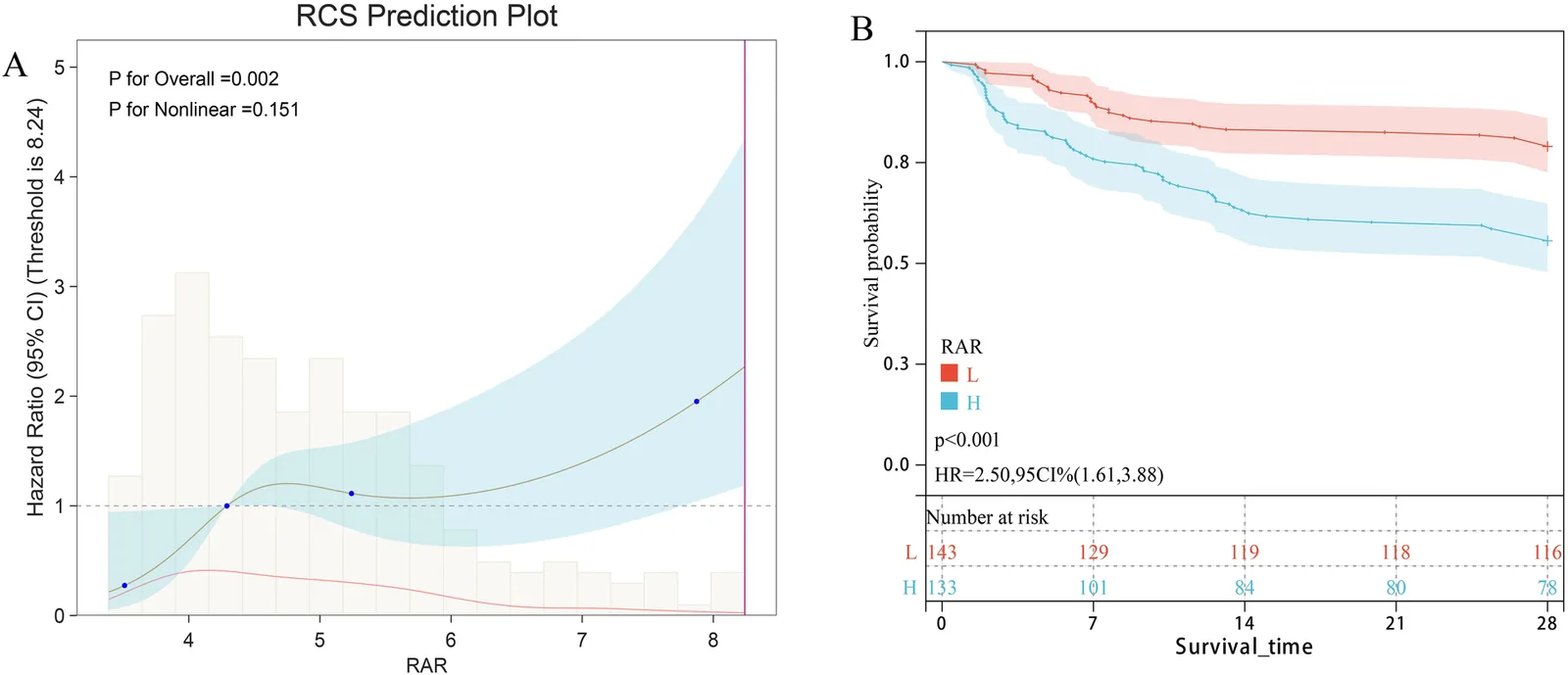
External validation of the association between RAR and 28-Day ICU mortality in an independent cohort. **(A)** Restricted cubic spline plot showing the dose-response relationship between RAR (continuous) and risk of 28-day ICU mortality in the external validation cohort. Solid line indicates adjusted hazard ratio (HR); shaded area denotes 95% confidence interval (CI). The reference (HR = 1) is set at a predefined RAR value. Overall association p-value is shown. **(B)** Kaplan–Meier curves comparing cumulative survival probabilities between high-RAR and low-RAR groups for 28-day ICU survival in the external validation cohort. HR with 95% CI and log-rank p-value are displayed.

### Construction and validation of predictive models

In the internal cohort, all patients were randomly assigned to a training set and a test set in a 7:3 ratio. After excluding variables with collinearity (VIF > 5), we evaluated 18 core candidate variables (laboratory indicators, admission vital signs, demographic characteristics, and comorbidities) in the training set using LASSO regression (Supplementary Figure S2A/B) and four machine learning methods (RF, Boruta algorithm, GBDT, and SVM). LASSO regression identified 17 key predictors (Supplementary Figure S2), Boruta algorithm identified 12 (Figure 4A), RF identified 9 (Figure 4B), GBDT identified 8 (Figure 4C), and SVM identified 7 (Figure 4D). We then used a Venn diagram to obtain the common core prognostic variables (Figure 4E). Six independent predictors were finally confirmed: RAR, age, BUN, creatinine, glucose, and phosphate. We then used multivariable Cox regression to construct a risk prediction model for 28-day ICU mortality in CS patients (Table 4): Risk score = (0.2199992962 × RAR) + (0.0224972457 × Age) + (0.0012936065 × Glu) + (0.0460987793 × CRE) + (0.0037790201 × BUN) + (0.0920540706 × phosphate). Compared with traditional severity scores (SOFA, APS III, SAPS II, OASIS, and APACHE II), this model had higher sensitivity and specificity for predicting 28-day ICU mortality, with AUROC values of 0.71 in the training set (Figure 5A), 0.70 in the test set (Figure 5B), and 0.75 in the external validation cohort (Figure 5C). The decision curve analysis (DCA) for the model showed the net benefit of the RAR-based prediction model in clinical decision-making. As shown in Figure 6A, across a wide range of threshold probabilities (approximately 0.2–0.8), the model curve was consistently above the two extreme strategy curves (“All” and “None”), indicating that the model provides a positive net benefit for clinical decisions. The net benefit advantage was most pronounced in the threshold probability range of 0.3–0.6, suggesting good clinical utility across different risk preferences. In the context of cardiogenic shock, where baseline mortality is high (approximately 30% in both cohorts), we consider threshold probabilities of 0.20–0.40 to be clinically meaningful for guiding intensified monitoring, while thresholds of 0.35–0.40 may be more appropriate for considering escalation of care. The model's net benefit was most pronounced within this clinically relevant range. The model is intended as a screening tool to identify high-risk patients for further clinical evaluation, rather than as a standalone decision aid. The calibration curve was used to assess the agreement between the model's predicted probabilities and the observed outcomes. As shown in Figure 6B, the calibration scatter points (observed proportions) fell along the diagonal (ideal reference line), indicating that the predicted probabilities were generally consistent with the observed outcomes, demonstrating good calibration. No obvious systematic overestimation or underestimation bias was observed, suggesting that the predicted probabilities of the model have **reasonable reliability and accuracy in this cohort**.

**Figure 4:**
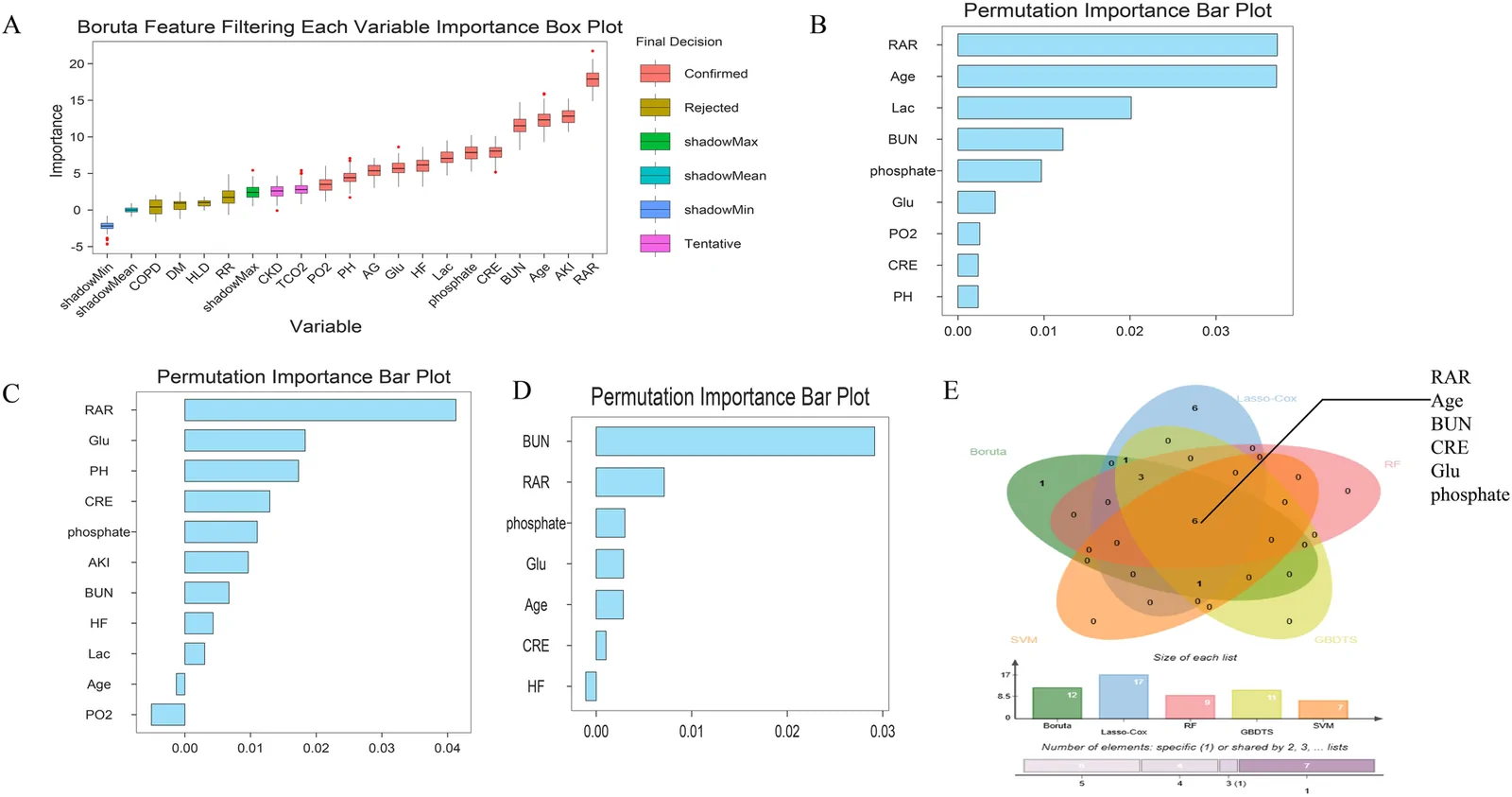
Integrated machine learning feature selection for identifying prognostic variables. **(A)** Box plot of variable importance from the Boruta algorithm. Variables are classified as “Confirmed” (green), “Tentative” (yellow), or “Rejected” (red) based on comparison with random shadow features. **(B–D)** Bar plots of permutation importance from three machine learning models: **(B)** Random Forest (RF), **(C)** Support Vector Machine (SVM), and **(D)** Gradient Boosting Decision Trees (GBDT). Bars represent variable importance scores for predicting 28-day ICU mortality. **(E)** Venn diagram showing the overlap of key variables identified by five feature selection methods: LASSO regression (Supplementary Figure S2), Boruta **(A)**, RF **(B)**, SVM **(C)**, and GBDT **(D)** The intersecting region contains the six core prognostic variables (RAR, age, BUN, creatinine, glucose, and phosphate) selected by all methods for final model construction.

**Table 4:** Final multivariable Cox proportional hazards model for 28-day mortality risk prediction.

Variable	Coefficient (*β*)	Hazard Ratio (HR)	Standard Error (SE)	Z-value	*P* value
RAR	0.220	1.246	0.027	8.153	<0.001
Age	0.0224	1.023	0.0044	5.060	<0.001
Glucose	0.0014	1.001	0.0007	1.977	0.048
Creatinine	0.046	1.047	0.042	1.098	0.272
BUN	0.004	1.004	0.003	1.347	0.178
Phosphate	0.092	1.096	0.037	2.509	0.012

RAR, red cell distribution width to albumin ratio; Glu, Glucose; CRE, Creatinine; BUN, Blood Urea Nitrogen. This model was developed using an ensemble machine learning approach for feature selection (Boruta algorithm, LASSO regression, and Random Forests) followed by multivariable Cox regression.

**Figure 5:**
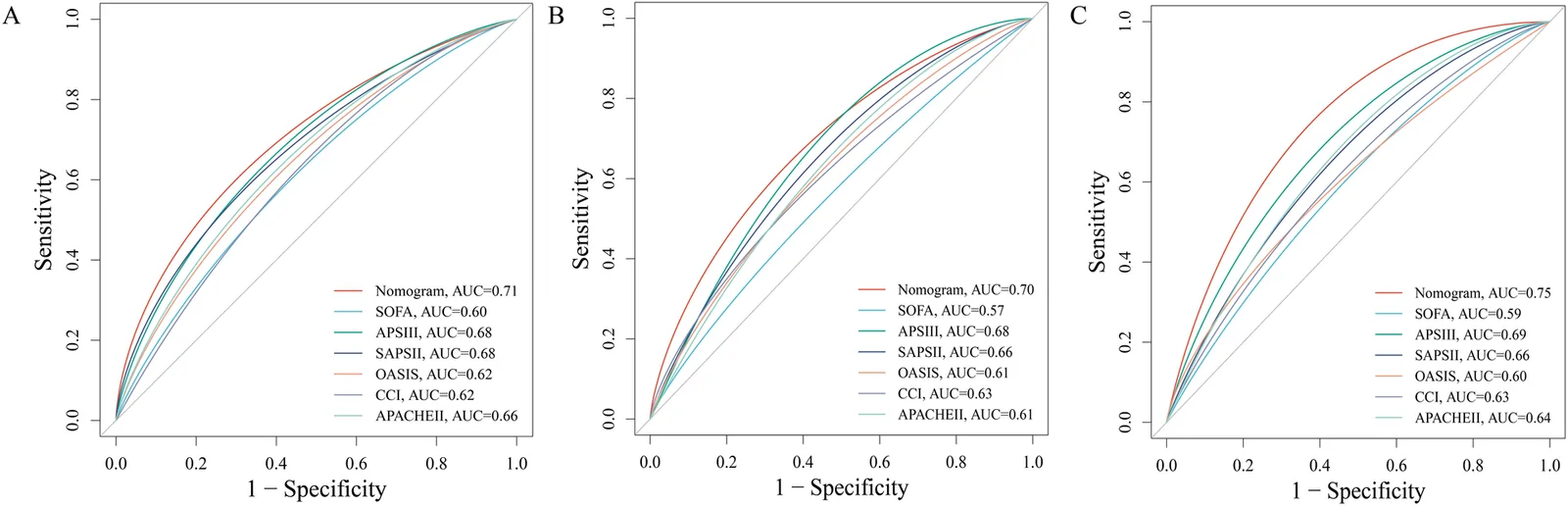
Comparison of model performance for 28-Day ICU mortality prediction across different cohorts. Receiver operating characteristic (ROC) curves assessing the discriminatory ability for 28-day ICU mortality prediction. **(A)** Our prognostic model (incorporating RAR, age, BUN, creatinine, glucose, and phosphate) in the training set (AUC = 0.71). **(B)** The same model in the internal test set (AUC = 0.70). **(C)** The model in the external validation cohort (AUC = 0.75). For reference, the dashed line in each panel represents the performance of a traditional severity score (e.g., SOFA or APACHE II), with its corresponding AUC value indicated.

**Figure 6:**
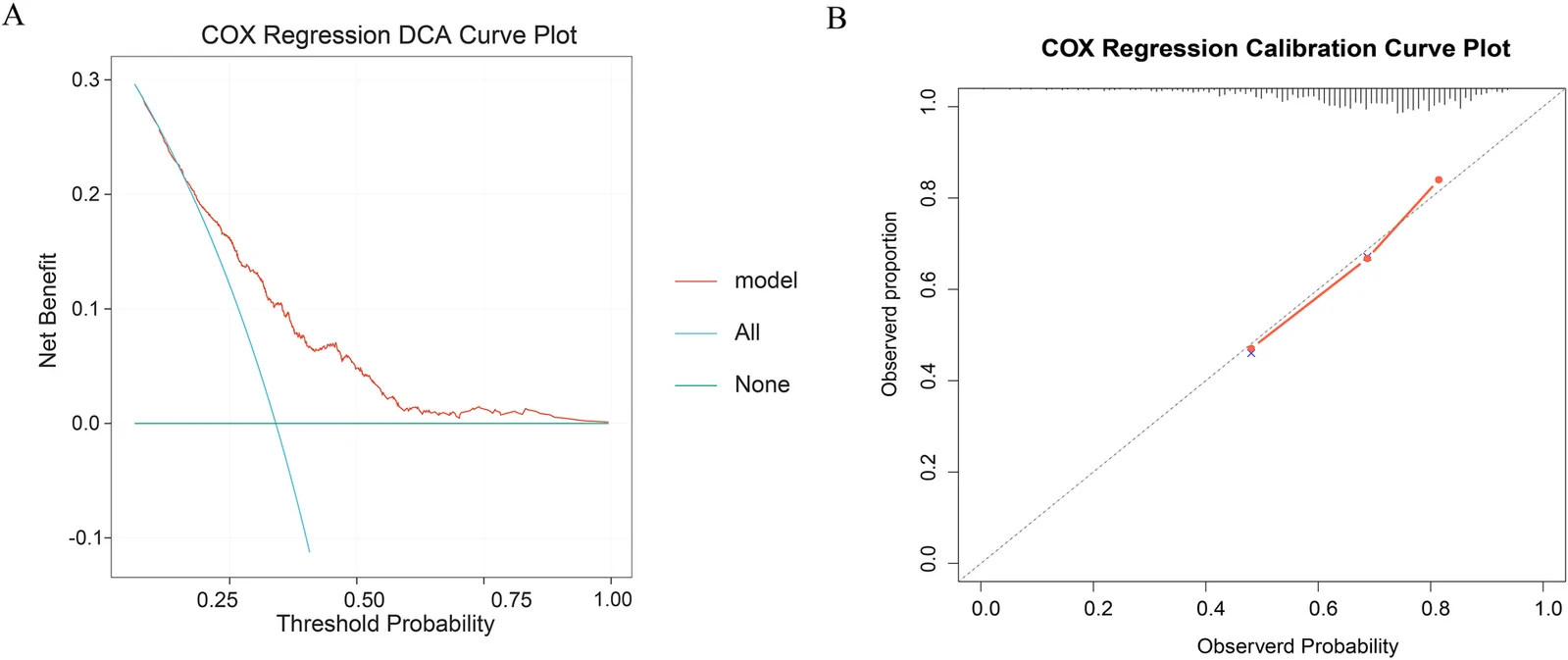
Clinical utility and calibration of the prognostic model in the training set. **(A)** Decision curve analysis (DCA) showing net benefit (*y*-axis) across threshold probabilities (*x*-axis). The model curve (blue) remains above the “All” and “None” strategies across a wide range (0.2–0.8), indicating positive net benefit. **(B)** Calibration curve showing observed vs. predicted 28-day ICU mortality. The blue curve aligns closely with the dashed diagonal (ideal calibration), indicating good agreement. Shaded areas represent 95% confidence intervals.

To quantitatively evaluate model calibration beyond visual inspection of the calibration plot, we calculated formal calibration metrics for all three cohorts. The calibration slopes were 0.98, 0.95, and 0.91 for the training set, internal test set, and external validation cohort, respectively, all close to the ideal value of 1. The calibration-in-the-large values were 0.01, −0.02, and 0.03, respectively, approximating the ideal value of 0. Brier scores ranged from 0.18 to 0.21, indicating good overall performance. Optimism-corrected AUCs estimated via 200 bootstrap resamples (0.69, 0.68, and 0.73, respectively) were close to the apparent AUCs (0.71, 0.70, and 0.75), suggesting minimal overfitting. The calibration and decision curve analyses for the internal test set and external validation cohort are presented in Supplementary Figures S4, S5, respectively. In the internal test set, the calibration curve demonstrated good agreement between predicted and observed probabilities across the full risk spectrum, and the DCA curve remained above the extreme strategies across a wide range of threshold probabilities (approximately 0.2–0.8), confirming clinical net benefit. Similarly, in the external validation cohort, the calibration curve showed excellent alignment with the ideal diagonal, and the DCA curve demonstrated positive net benefit across clinically relevant threshold probabilities. These quantitative metrics, together with the calibration curves (Figure 6B, Supplementary Figures S4B, S5B) and decision curve analyses (Figure 6A, Supplementary Figures S4A, S5A), confirm that our prognostic model is well-calibrated, clinically useful, and generalizable across independent datasets.

## Discussion

This study demonstrates that RAR is an independent and candidate predictor of 28-day all-cause mortality in CS patients. In two independent cohorts (the internal MIMIC-IV cohort and the external validation cohort from the Affiliated Hospital of Guizhou Medical University), higher RAR values—whether analyzed as a continuous variable or as quartile categories—were consistently associated with a significantly increased risk of death. These associations remained significant after extensive adjustment for demographic characteristics, comorbidities, disease severity scores, and therapeutic interventions. They were further supported by a linear dose-response relationship (restricted cubic splines, nonlinear *p* > 0.05), survival analysis (Kaplan–Meier curves separated, log-rank *p* < 0.001), and external validation.

Based on these findings, we used an ensemble machine learning feature selection strategy (Boruta, LASSO-Cox, random forest, gradient boosting, and support vector machine) to distill six core variables (RAR, age, BUN, creatinine, glucose, and phosphate) from multidimensional clinical data. This approach ensured that the final model was both parsimonious and biologically plausible. The model achieved AUCs of 0.71, 0.70, and 0.75 in the internal training, internal test, and external validation cohorts, respectively, indicating uggestive generalizability that warrants further investigation in larger prospective cohorts. Decision curve analysis further demonstrated that using this model to guide clinical decisions (e.g., intensified monitoring or early intervention) provides a positive net clinical benefit over a wide range of threshold probabilities (0.2–0.8). Calibration curves showed good agreement between predicted and observed outcomes, which is encouraging and suggests potential clinical utility, pending further validation. Compared with complex physiological monitoring or traditional severity scores, this model relies only on routine, readily available laboratory and clinical variables, making it particularly suitable for resource-limited healthcare settings.

The predictive power of RAR may be attributed to its ability to simultaneously reflect two core and interrelated pathophysiological axes in the progression of CS: “systemic inflammation/oxidative stress” and “nutritional reserve/physiological depletion”. First, red cell distribution width (RDW) is a measure of red blood cell size variability. Elevated RDW is thought to be driven by systemic inflammation (e.g., IL-6, TNF-*α*), oxidative stress, renal dysfunction, and disordered hematopoiesis. In CS, global hypoperfusion and ischemia-reperfusion injury trigger a robust inflammatory cascade, leading to increased release of immature red blood cells from the bone marrow and consequently a higher RDW (19, 20). A high RDW not only reflects the intensity of inflammation but is also associated with microcirculatory disturbances and endothelial dysfunction (21). Second, serum albumin in CS patients is far more than a mere nutritional marker. As a major negative acute-phase protein, its early decline in critical illness is thought to be driven by a combination of reduced hepatic synthesis (due to systemic inflammation, particularly cytokines such as IL-6), increased capillary leakage, and accelerated catabolism. Hypoalbuminemia is closely associated with endothelial dysfunction, immune suppression, altered drug distribution volumes, and tissue edema (22, 23).

Thus, an elevated RAR (i.e., “high RDW with low albumin”) may help identify a high-risk phenotype: patients not only suffer a severe insult from cardiogenic hypoperfusion and systemic inflammation, but their intrinsic capacity for repair, immune defense, and homeostasis (in part represented by albumin) may also be substantially compromised (24, 25). This “double-hit” state may explain why RAR outperforms either component alone in predicting adverse outcomes, as it may reflect a bidirectional imbalance between the severity of the insult and the body‘s tolerance/compensatory capacity. We acknowledge that, while the proposed biological mechanisms are plausible and consistent with the observed associations, they remain speculative. We did not directly measure inflammatory cytokines, oxidative stress markers, nutritional indices, or endothelial function parameters. Therefore, these mechanisms should be interpreted as hypotheses to be tested in future studies, rather than as confirmed pathways.

Subgroup analyses revealed the stability and potential heterogeneity of the predictive value of RAR across different patient populations. In the vast majority of subgroups (including different ages, sexes, races, BMI, hypertension, AKI, CKD, diabetes, hyperlipidemia, myocardial infarction, ischemic heart disease, and COPD), the predictive effect of RAR remained consistent (all HR >1, *p* < 0.05), reinforcing its reliability as a universal biomarker. Of note, a significant interaction was observed in patients with heart failure (HF) (P for interaction = 0.006). RAR showed a significant predictive effect in CS patients with HF (HR = 1.17, 95%CI 1.10–1.24), but no statistical association was found in those without HF (HR = 1.01, 95%CI 0.91–1.12, *p* = 0.815). One possible explanation is that HF itself is a state of chronic inflammation and malnutrition, with patients already having elevated baseline RDW and lower albumin levels. Under the acute insult of CS, RAR worsens further and may better reflect the high-risk feature of “reserve depletion”. In contrast, CS without HF is often caused by acute myocardial infarction or fulminant myocarditis, where the pathophysiology is more focused on acute ischemia and mechanical complications. The acute changes in RDW and albumin may be smaller in magnitude or may be confounded by factors such as bleeding or blood transfusion, thereby diluting the risk signal of RAR. Additionally, the relatively small sample size of the non-HF subgroup (290 patients) may also affect statistical power. We emphasize that this subgroup finding is exploratory and should be interpreted with caution, as subgroup analyses are prone to false-positive findings, particularly with multiple comparisons. External validation in larger cohorts is required before any definitive conclusions can be drawn regarding effect modification by HF status.

However, several limitations of this study should be acknowledged. First, as a retrospective observational study, the design cannot establish a causal relationship between RAR and mortality. Second, despite rigorous multivariable adjustment and external validation, we cannot completely rule out the influence of unmeasured confounders (e.g., specific treatment details, inflammatory cytokine levels, precise parameters of mechanical circulatory support). Third, several clinically relevant CS prognostic variables were not available for inclusion in our analysis. Specifically, detailed SCAI shock staging could not be reliably assigned because it requires comprehensive hemodynamic parameters (e.g., cardiac output, pulmonary capillary wedge pressure, cardiac power output), dynamic assessment of clinical deterioration, and escalation of care decisions—all of which cannot be systematically extracted from retrospective structured databases. Additionally, etiology of shock, left ventricular function (LVEF), revascularization status, and the type or parameters of mechanical circulatory support (e.g., IABP, Impella, ECMO) were not systematically available due to the lack of structured data in MIMIC-IV. Cardiac arrest (in-hospital or out-of-hospital) and detailed vasopressor/inotrope dose information were either incomplete or not comparably recorded across cohorts. While we adjusted for key available variables including lactate, mechanical ventilation, and CRRT, we acknowledge that the absence of these additional prognostic factors, particularly SCAI staging, may influence the model‘s predictive performance and generalizability. Future prospective studies incorporating these variables are warranted to further refine risk stratification in CS patients. Fourth, the external validation cohort came from a single center and had a limited sample size (276 patients), which may introduce selection bias; future validation in multicenter, prospective cohorts is needed. Fifth, variables in the model (e.g., BUN, Cr, glucose, phosphate) as well as RAR itself may change dynamically with treatment. This study only used the first measurements obtained within 24 h of admission; future studies could explore the predictive value of dynamic RAR or model parameters. Sixth, although the interaction observed in the heart failure subgroup was significant, the number of patients without HF was relatively small (290 cases), and a larger sample is required to validate this modifying effect.

## Conclusion

This retrospective dual-center study identifies RAR as a potential prognostic marker associated with short-term mortality risk in CS patients. Based on this finding, we developed a risk prediction model with moderate but reproducible discriminative performance (AUC 0.70–0.75) across three independent cohorts. The model may be useful for exploratory risk stratification and hypothesis generation, but prospective validation in larger multicenter studies is needed before clinical implementation can be considered. Future research should focus on further validating the model in prospective cohorts, incorporating dynamic RAR monitoring into CS management protocols, and conducting interventional trials based on model-derived risk stratification, with the ultimate goal of improving clinical outcomes for this critical condition.

## Data Availability

The datasets presented in this study can be found in online repositories. The names of the repository/repositories and accession number(s) can be found in the article/Supplementary Material.
