# Supplementary material for: The red blood cell distribution width-to-albumin ratio and 28-day mortality in patients with severe cardiogenic shock: a retrospective cohort study based on MIMIC-IV with single-center external validation
Source: Front Med (Lausanne). 2026 Sep 30;13:1857535. doi: 10.3389/fmed.2026.1857535 (PMC13661497; doi:10.3389/fmed.2026.1857535)
Supplement: Supplementary file 1 [file Datasheet1.pdf]

```sql
asic AS (
    WITH
    --Diagnosis included
    -- Cardiogenic shock
    includedDiagnose1 AS (
        SELECT DISTINCT hadm_id
        FROM mimiciv_hosp.diagnoses_icd
        WHERE icd_code IN ('78551')
    ),
    -- Cardiogenic shock
    includedDiagnose2 AS (
        SELECT DISTINCT hadm_id
        FROM mimiciv_hosp.diagnoses_icd
        WHERE icd_code IN ('R570')
    )
    SELECT
        DISTINCT ON (icu.subject_id)
        icu.subject_id,
        icu.stay_id,
        icu.hadm_id,
        icu.los_icu
    FROM mimiciv_derived.icustay_detail AS icu
    JOIN mimiciv_derived.age AS age ON age.hadm_id = icu.hadm_id
    WHERE
        TRUE
        AND age.age BETWEEN 18 AND 120
        AND icu.los_icu > 24.0/24.0
        AND icu.first_icu_stay = true
        AND icu.first_hosp_stay = true
        AND (icu.survival_day >= 1 OR icu.survival_day IS NULL)
        --Diagnosis included
        AND (
            -- Cardiogenic shock
            icu.hadm_id IN (SELECT hadm_id FROM includedDiagnose1)
            -- Cardiogenic shock
            OR icu.hadm_id IN (SELECT hadm_id FROM includedDiagnose2)
        )
)
--SELECT * FROM basic;

SELECT DISTINCT(subject.subject_id),
    subject.stay_id,
    subject.hadm_id,
    ROUND(age.age,0) AS age,
```

```
    icu.gender,
    icu.race,
    weight.weight,
    height.height,
    adm.insurance,
    adm.language,
    adm.marital_status
FROM basic AS subject
LEFT JOIN mimiciv_derived.age AS age ON subject.hadm_id = age.hadm_id
LEFT JOIN mimiciv_derived.icustay_detail AS icu ON subject.stay_id = icu.stay_id
LEFT JOIN mimiciv_derived.first_day_weight AS weight ON subject.stay_id = weight.stay_id
LEFT JOIN mimiciv_hosp.admissions AS adm ON subject.hadm_id = adm.hadm_id
LEFT JOIN mimiciv_derived.first_day_height AS height ON subject.stay_id = height.stay_id
ORDER BY subject.subject_id;
basic AS (
    WITH
    --Diagnosis included
    -- Cardiogenic shock
    includedDiagnose1 AS (
        SELECT DISTINCT hadm_id
        FROM mimiciv_hosp.diagnoses_icd
        WHERE icd_code IN ('78551')
    ),
    -- Cardiogenic shock
    includedDiagnose2 AS (
        SELECT DISTINCT hadm_id
        FROM mimiciv_hosp.diagnoses_icd
        WHERE icd_code IN ('R570')
    )
    SELECT
        DISTINCT ON (icu.subject_id)
        icu.subject_id,
        icu.stay_id,
        icu.hadm_id,
        icu.los_icu
    FROM mimiciv_derived.icustay_detail AS icu
    JOIN mimiciv_derived.age AS age ON age.hadm_id = icu.hadm_id
    WHERE
        TRUE
        AND age.age BETWEEN 18 AND 120
        AND icu.los_icu > 24.0/24.0
        AND icu.first_icu_stay = true
        AND icu.first_hosp_stay = true
        AND (icu.survival_day >= 1 OR icu.survival_day IS NULL)
```

```
        --Diagnosis included
        AND (
            -- Cardiogenic shock
            icu.hadm_id IN (SELECT hadm_id FROM includedDiagnose1)
            -- Cardiogenic shock
            OR icu.hadm_id IN (SELECT hadm_id FROM includedDiagnose2)
        )
)
--SELECT * FROM basic;

,
disease AS (
SELECT
    DISTINCT subject.hadm_id,
    -- HTN
    MAX(CASE WHEN dia.icd_code IN ('4019','I10','4011','I161','4010') THEN 1 ELSE 0 END)
AS HTN,
    -- AKI
    MAX(CASE WHEN dia.icd_code IN ('5849','N179','5845','N170') THEN 1 ELSE 0 END)
AS AKI,
    -- CKD
    MAX(CASE WHEN dia.icd_code IN
('40390','5859','I129','N189','N183','5853','I130','I120','5854','N184','5852','40310','N182','5855','N
185','E1122') THEN 1 ELSE 0 END) AS CKD,
    -- T2DM
    MAX(CASE WHEN dia.icd_code IN
('E119','E1122','E1165','E1140','E1151','E11319','E1142','E1121','E11649','E11621','E1169','E114
3','E1152','E118','E11610','E11622','E1110','E11628','E1139','E1136','25000','25060','25040','2505
0','25002','25080','25062','25042','25082','25052','25070','25012','25092','25072','25090') THEN 1
ELSE 0 END) AS T2DM,
    --T1DM
    MAX(CASE WHEN dia.icd_code IN
('E1022','E10319','E1065','E1040','E1043','E10649','E1010','E1021','E109','E1042','E1051','E1062
1','25061','25001','25051','25041','25063','25013','25053','25043','25081') THEN 1 ELSE 0 END)
AS T1DM,
    -- HLD
    MAX(CASE WHEN dia.icd_code IN ('2724','E785','E7849','E782','E784','2722') THEN 1
ELSE 0 END) AS HLD,
    --HF
    MAX(CASE WHEN dia.icd_code IN
('4280','42832','42822','I5032','42833','I5033','I5022','42823','I5023','I509','42830','42843','42831','I
5030','42821','42842','I5021','I5020','42820','I5031','I5043','I5042','40491','40291','42841','4289','4
2840','I5084','I50810','I5041','I5082','I5040','4281','I50814','I50811','I50813','I50812','40201','4049
2','I5089','I5083') THEN 1 ELSE 0 END) AS HF,
```

```
    -- MI
    MAX(CASE WHEN dia.icd_code IN
('41000','41001','41002','41010','41011','41012','41020','41021','41022','41030','41031','41032','410
40','41041','41042','41050','41051','41052','41080','41081','41082','41090','41091','41092','I21','I21
9','I230','I231','I232','I233','I234','I235','I236','I238','I210','I2101','I2102','I2109','I211','I2111','I211
9','I2121','I2129','I213','I214','I21A1','I21A9','I222') THEN 1 ELSE 0 END) AS MI,
    -- IHD
    MAX(CASE WHEN dia.icd_code IN
('I21A1','I237','41001','41002','41010','41011','41012','41021','41022','41031','41032','41040','4104
1','41042','41051','41052','41061','41062','41070','41071','41072','41081','41082','41090','41091','41
092','4110','4111','41181','41189','412','4130','4131','4139','41400','41401','41402','41404','41405','4
1407','41410','41411','41412','41419','4142','4143','4144','4148','4149','I200','I201','I208','I209','I21
01','I2102','I2109','I2111','I2119','I2121','I2129','I213','I219','I21A9','I220','I221','I222','I228','I229','
I232','I234','I235','I236','I238','I240','I241','I248','I249','I2510','I25110','I25111','I25118','I25119','I2
52','I253','I2541','I2542','I255','I256','I25700','I25708','I25709','I25710','I25711','I25718','I25719','I
25720','I25721','I25728','I25729','I25758','I25790','I25798','I25810','I25811','I2582','I2583','I2584','
I2589','I259','I214') THEN 1 ELSE 0 END) AS IHD,
    - COPD
    MAX(CASE WHEN dia.icd_code IN
('J430','490','4910','4911','49120','49121','49122','4918','4919','4928','4940','4941','496','J40','J410','
J411','J42','J431','J432','J438','J439','J44','J440','J441','J449','4920') THEN 1 ELSE 0 END) AS
COPD
  FROM basic AS subject
  INNER JOIN mimiciv_hosp.diagnoses_icd AS dia
  ON subject.hadm_id = dia.hadm_id
  GROUP BY subject.hadm_id
)

SELECT DISTINCT
    basic.subject_id,
    basic.stay_id,
    basic.hadm_id,
    --HTN
    COALESCE(disease.HTN, 0) AS HTN,
    --AKI
    COALESCE(disease.AKI, 0) AS AKI,
    --CKD
    COALESCE(disease.CKD, 0) AS CKD,
    --T2DM
    COALESCE(disease.T2DM, 0) AS T2DM,
    --T1DM
    COALESCE(disease.T1DM, 0) AS T1DM,
    --HLD
    COALESCE(disease.HLD, 0) AS HLD,
```

```
    --HF
    COALESCE(disease.HF, 0) AS HF,
    --MI
    COALESCE(disease.MI, 0) AS MI,
    --HD
    COALESCE(disease.IHD, 0) AS IHD,
    --COPD
    COALESCE(disease.COPD, 0) AS COPD
FROM basic
LEFT JOIN disease ON basic.hadm_id = disease.hadm_id
ORDER BY basic.subject_id;
basic AS (
    WITH
    --Diagnosis included
    -- Cardiogenic shock
    includedDiagnose1 AS (
        SELECT DISTINCT hadm_id
        FROM mimiciv_hosp.diagnoses_icd
        WHERE icd_code IN ('78551')
    ),
    -- Cardiogenic shock
    includedDiagnose2 AS (
        SELECT DISTINCT hadm_id
        FROM mimiciv_hosp.diagnoses_icd
        WHERE icd_code IN ('R570')
    )
    SELECT
        DISTINCT ON (icu.subject_id)
        icu.subject_id,
        icu.stay_id,
        icu.hadm_id,
        icu.los_icu
    FROM mimiciv_derived.icustay_detail AS icu
    JOIN mimiciv_derived.age AS age ON age.hadm_id = icu.hadm_id
    WHERE
        TRUE
        AND age.age BETWEEN 18 AND 120
        AND icu.los_icu > 24.0/24.0
        AND icu.first_icu_stay = true
        AND icu.first_hosp_stay = true
        AND (icu.survival_day >= 1 OR icu.survival_day IS NULL)
        --Diagnosis included
        AND (
            -- Cardiogenic shock
```

```sql
            icu.hadm_id IN (SELECT hadm_id FROM includedDiagnose1)
            -- Cardiogenic shock
            OR icu.hadm_id IN (SELECT hadm_id FROM includedDiagnose2)
        )
)
--SELECT * FROM basic;
FIRST_HR AS (
SELECT
    DISTINCT ON (LAB.STAY_ID)
    LAB.STAY_ID,
    LAB.VALUENUM,
    LAB.VALUEUOM,
    LAB.charttime AS HR_outputevents_charttime,
    LAB.storetime AS HR_outputevents_storetime
    FROM basic AS SUBJECT,
    MIMICIV_DERIVED.ICUSTAY_DETAIL AS ICU,
    MIMICIV_ICU.CHARTEVENTS AS LAB
    WHERE LAB.ITEMID = 220045
    AND LAB.CHARTTIME >= ICU.ICU_INTIME
    AND SUBJECT.STAY_ID = ICU.STAY_ID
    AND SUBJECT.STAY_ID = LAB.STAY_ID
    ORDER BY LAB.STAY_ID,LAB.CHARTTIME
),
FIRST_NBPs AS (
SELECT
    DISTINCT ON (LAB.STAY_ID)
    LAB.STAY_ID,
    LAB.VALUENUM,
    LAB.VALUEUOM,
    LAB.charttime AS NBPs_outputevents_charttime,
    LAB.storetime AS NBPs_outputevents_storetime
    FROM basic AS SUBJECT,
    MIMICIV_DERIVED.ICUSTAY_DETAIL AS ICU,
    MIMICIV_ICU.CHARTEVENTS AS LAB
    WHERE LAB.ITEMID = 220179
    AND LAB.CHARTTIME >= ICU.ICU_INTIME
    AND SUBJECT.STAY_ID = ICU.STAY_ID
    AND SUBJECT.STAY_ID = LAB.STAY_ID
    ORDER BY LAB.STAY_ID,LAB.CHARTTIME
),
FIRST_NBPd AS (
SELECT
    DISTINCT ON (LAB.STAY_ID)
    LAB.STAY_ID,
```

```
    LAB.VALUENUM,
    LAB.VALUEUOM,
    LAB.charttime AS NBPd_outputevents_charttime,
    LAB.storetime AS NBPd_outputevents_storetime
    FROM basic AS SUBJECT,
    MIMICIV_DERIVED.ICUSTAY_DETAIL AS ICU,
    MIMICIV_ICU.CHARTEVENTS AS LAB
    WHERE LAB.ITEMID = 220180
    AND LAB.CHARTTIME >= ICU.ICU_INTIME
    AND SUBJECT.STAY_ID = ICU.STAY_ID
    AND SUBJECT.STAY_ID = LAB.STAY_ID
    ORDER BY LAB.STAY_ID,LAB.CHARTTIME
),
FIRST_NBPm AS (
SELECT
    DISTINCT ON (LAB.STAY_ID)
    LAB.STAY_ID,
    LAB.VALUENUM,
    LAB.VALUEUOM,
    LAB.charttime AS NBPm_outputevents_charttime,
    LAB.storetime AS NBPm_outputevents_storetime
    FROM basic AS SUBJECT,
    MIMICIV_DERIVED.ICUSTAY_DETAIL AS ICU,
    MIMICIV_ICU.CHARTEVENTS AS LAB
    WHERE LAB.ITEMID = 220181
    AND LAB.CHARTTIME >= ICU.ICU_INTIME
    AND SUBJECT.STAY_ID = ICU.STAY_ID
    AND SUBJECT.STAY_ID = LAB.STAY_ID
    ORDER BY LAB.STAY_ID,LAB.CHARTTIME
),
FIRST_ABPs AS (
SELECT
    DISTINCT ON (LAB.STAY_ID)
    LAB.STAY_ID,
    LAB.VALUENUM,
    LAB.VALUEUOM,
    LAB.charttime AS ABPs_outputevents_charttime,
    LAB.storetime AS ABPs_outputevents_storetime
    FROM basic AS SUBJECT,
    MIMICIV_DERIVED.ICUSTAY_DETAIL AS ICU,
    MIMICIV_ICU.CHARTEVENTS AS LAB
    WHERE LAB.ITEMID = 220050
    AND LAB.CHARTTIME >= ICU.ICU_INTIME
    AND SUBJECT.STAY_ID = ICU.STAY_ID
```

```
    AND SUBJECT.STAY_ID = LAB.STAY_ID
    ORDER BY LAB.STAY_ID,LAB.CHARTTIME
),
FIRST_ABPd AS (
SELECT
    DISTINCT ON (LAB.STAY_ID)
    LAB.STAY_ID,
    LAB.VALUENUM,
    LAB.VALUEUOM,
    LAB.charttime AS ABPd_outputevents_charttime,
    LAB.storetime AS ABPd_outputevents_storetime
    FROM basic AS SUBJECT,
    MIMICIV_DERIVED.ICUSTAY_DETAIL AS ICU,
    MIMICIV_ICU.CHARTEVENTS AS LAB
    WHERE LAB.ITEMID = 220051
    AND LAB.CHARTTIME >= ICU.ICU_INTIME
    AND SUBJECT.STAY_ID = ICU.STAY_ID
    AND SUBJECT.STAY_ID = LAB.STAY_ID
    ORDER BY LAB.STAY_ID,LAB.CHARTTIME
),
FIRST_ABPm AS (
SELECT
    DISTINCT ON (LAB.STAY_ID)
    LAB.STAY_ID,
    LAB.VALUENUM,
    LAB.VALUEUOM,
    LAB.charttime AS ABPm_outputevents_charttime,
    LAB.storetime AS ABPm_outputevents_storetime
    FROM basic AS SUBJECT,
    MIMICIV_DERIVED.ICUSTAY_DETAIL AS ICU,
    MIMICIV_ICU.CHARTEVENTS AS LAB
    WHERE LAB.ITEMID = 220052
    AND LAB.CHARTTIME >= ICU.ICU_INTIME
    AND SUBJECT.STAY_ID = ICU.STAY_ID
    AND SUBJECT.STAY_ID = LAB.STAY_ID
    ORDER BY LAB.STAY_ID,LAB.CHARTTIME
),
FIRST_RR AS (
SELECT
    DISTINCT ON (LAB.STAY_ID)
    LAB.STAY_ID,
    LAB.VALUENUM,
    LAB.VALUEUOM,
    LAB.charttime AS RR_outputevents_charttime,
```

```
    LAB.storetime AS RR_outputevents_storetime
    FROM basic AS SUBJECT,
    MIMICIV_DERIVED.ICUSTAY_DETAIL AS ICU,
    MIMICIV_ICU.CHARTEVENTS AS LAB
    WHERE LAB.ITEMID = 220210
    AND LAB.CHARTTIME >= ICU.ICU_INTIME
    AND SUBJECT.STAY_ID = ICU.STAY_ID
    AND SUBJECT.STAY_ID = LAB.STAY_ID
    ORDER BY LAB.STAY_ID,LAB.CHARTTIME
),
FIRST_SpO2 AS (
SELECT
    DISTINCT ON (LAB.STAY_ID)
    LAB.STAY_ID,
    LAB.VALUENUM,
    LAB.VALUEUOM,
    LAB.charttime AS SpO2_outputevents_charttime,
    LAB.storetime AS SpO2_outputevents_storetime
    FROM basic AS SUBJECT,
    MIMICIV_DERIVED.ICUSTAY_DETAIL AS ICU,
    MIMICIV_ICU.CHARTEVENTS AS LAB
    WHERE LAB.ITEMID = 220277
    AND LAB.CHARTTIME >= ICU.ICU_INTIME
    AND SUBJECT.STAY_ID = ICU.STAY_ID
    AND SUBJECT.STAY_ID = LAB.STAY_ID
    ORDER BY LAB.STAY_ID,LAB.CHARTTIME
),
FIRST_TemperatureF AS (
SELECT
    DISTINCT ON (LAB.STAY_ID)
    LAB.STAY_ID,
    LAB.VALUENUM,
    LAB.VALUEUOM,
    LAB.charttime AS TemperatureF_outputevents_charttime,
    LAB.storetime AS TemperatureF_outputevents_storetime
    FROM basic AS SUBJECT,
    MIMICIV_DERIVED.ICUSTAY_DETAIL AS ICU,
    MIMICIV_ICU.CHARTEVENTS AS LAB
    WHERE LAB.ITEMID = 223761
    AND LAB.CHARTTIME >= ICU.ICU_INTIME
    AND SUBJECT.STAY_ID = ICU.STAY_ID
    AND SUBJECT.STAY_ID = LAB.STAY_ID
    ORDER BY LAB.STAY_ID,LAB.CHARTTIME
)
```

```
SELECT
    DISTINCT(SUBJECT.SUBJECT_ID),
    SUBJECT.STAY_ID,
    SUBJECT.HADM_ID,
    ROUND(FIRST_HR.VALUENUM::numeric,2) AS FIRST_HR,
    FIRST_HR.VALUEUOM AS FIRST_HR_UOM,
    FIRST_HR.HR_outputevents_charttime,
    FIRST_HR.HR_outputevents_storetime,
    ROUND(FIRST_NBPs.VALUENUM::numeric,2) AS FIRST_NBPs,
    FIRST_NBPs.VALUEUOM AS FIRST_NBPs_UOM,
    FIRST_NBPs.NBPs_outputevents_charttime,
    FIRST_NBPs.NBPs_outputevents_storetime,
    ROUND(FIRST_NBPd.VALUENUM::numeric,2) AS FIRST_NBPd,
    FIRST_NBPd.VALUEUOM AS FIRST_NBPd_UOM,
    FIRST_NBPd.NBPd_outputevents_charttime,
    FIRST_NBPd.NBPd_outputevents_storetime,
    ROUND(FIRST_NBPm.VALUENUM::numeric,2) AS FIRST_NBPm,
    FIRST_NBPm.VALUEUOM AS FIRST_NBPm_UOM,
    FIRST_NBPm.NBPm_outputevents_charttime,
    FIRST_NBPm.NBPm_outputevents_storetime,
    ROUND(FIRST_ABPs.VALUENUM::numeric,2) AS FIRST_ABPs,
    FIRST_ABPs.VALUEUOM AS FIRST_ABPs_UOM,
    FIRST_ABPs.ABPs_outputevents_charttime,
    FIRST_ABPs.ABPs_outputevents_storetime,
    ROUND(FIRST_ABPd.VALUENUM::numeric,2) AS FIRST_ABPd,
    FIRST_ABPd.VALUEUOM AS FIRST_ABPd_UOM,
    FIRST_ABPd.ABPd_outputevents_charttime,
    FIRST_ABPd.ABPd_outputevents_storetime,
    ROUND(FIRST_ABPm.VALUENUM::numeric,2) AS FIRST_ABPm,
    FIRST_ABPm.VALUEUOM AS FIRST_ABPm_UOM,
    FIRST_ABPm.ABPm_outputevents_charttime,
    FIRST_ABPm.ABPm_outputevents_storetime,
    ROUND(FIRST_RR.VALUENUM::numeric,2) AS FIRST_RR,
    FIRST_RR.VALUEUOM AS FIRST_RR_UOM,
    FIRST_RR.RR_outputevents_charttime,
    FIRST_RR.RR_outputevents_storetime,
    ROUND(FIRST_SpO2.VALUENUM::numeric,2) AS FIRST_SpO2,
    FIRST_SpO2.VALUEUOM AS FIRST_SpO2_UOM,
    FIRST_SpO2.SpO2_outputevents_charttime,
    FIRST_SpO2.SpO2_outputevents_storetime,
    ROUND(FIRST_TemperatureF.VALUENUM::numeric,2) AS FIRST_TemperatureF,
    FIRST_TemperatureF.VALUEUOM AS FIRST_TemperatureF_UOM,
    FIRST_TemperatureF.TemperatureF_outputevents_charttime,
```

```
    FIRST_TemperatureF.TemperatureF_outputevents_storetime
    FROM basic AS SUBJECT
LEFT JOIN FIRST_HR ON SUBJECT.STAY_ID = FIRST_HR.STAY_ID
LEFT JOIN FIRST_NBPs ON SUBJECT.STAY_ID = FIRST_NBPs.STAY_ID
LEFT JOIN FIRST_NBPd ON SUBJECT.STAY_ID = FIRST_NBPd.STAY_ID
LEFT JOIN FIRST_NBPm ON SUBJECT.STAY_ID = FIRST_NBPm.STAY_ID
LEFT JOIN FIRST_ABPs ON SUBJECT.STAY_ID = FIRST_ABPs.STAY_ID
LEFT JOIN FIRST_ABPd ON SUBJECT.STAY_ID = FIRST_ABPd.STAY_ID
LEFT JOIN FIRST_ABPm ON SUBJECT.STAY_ID = FIRST_ABPm.STAY_ID
LEFT JOIN FIRST_RR ON SUBJECT.STAY_ID = FIRST_RR.STAY_ID
LEFT JOIN FIRST_SpO2 ON SUBJECT.STAY_ID = FIRST_SpO2.STAY_ID
LEFT JOIN FIRST_TemperatureF ON SUBJECT.STAY_ID = FIRST_TemperatureF.STAY_ID
ORDER BY SUBJECT.SUBJECT_ID;
basic AS (
    WITH
    --Diagnosis included
    -- Cardiogenic shock
    includedDiagnose1 AS (
        SELECT DISTINCT hadm_id
        FROM mimiciv_hosp.diagnoses_icd
        WHERE icd_code IN ('78551')
    ),
    -- Cardiogenic shock
    includedDiagnose2 AS (
        SELECT DISTINCT hadm_id
        FROM mimiciv_hosp.diagnoses_icd
        WHERE icd_code IN ('R570')
    )
    SELECT
        DISTINCT ON (icu.subject_id)
        icu.subject_id,
        icu.stay_id,
        icu.hadm_id,
        icu.los_icu
    FROM mimiciv_derived.icustay_detail AS icu
    JOIN mimiciv_derived.age AS age ON age.hadm_id = icu.hadm_id
    WHERE
        TRUE
        AND age.age BETWEEN 18 AND 120
        AND icu.los_icu > 24.0/24.0
        AND icu.first_icu_stay = true
        AND icu.first_hosp_stay = true
        AND (icu.survival_day >= 1 OR icu.survival_day IS NULL)
        --Diagnosis included
```

```
        AND (
            -- Cardiogenic shock
            icu.hadm_id IN (SELECT hadm_id FROM includedDiagnose1)
            -- Cardiogenic shock
            OR icu.hadm_id IN (SELECT hadm_id FROM includedDiagnose2)
        )
)
--SELECT * FROM basic;

SELECT
    DISTINCT(subject.subject_id),
    subject.stay_id,
    subject.hadm_id,
    sofa.sofa,
    apsiii.apsiii,
    sirs.sirs,
    sapsii.sapsii,
    oasis.oasis,
    charlson.charlson_comorbidity_index AS charlson,
    apacheii.apacheii AS apacheii

FROM basic AS subject
LEFT JOIN mimiciv_derived.first_day_sofa AS sofa ON subject.stay_id = sofa.stay_id
LEFT JOIN mimiciv_derived.apsiii AS apsiii ON subject.stay_id = apsiii.stay_id
LEFT JOIN mimiciv_derived.sirs AS sirs ON subject.stay_id = sirs.stay_id
LEFT JOIN mimiciv_derived.sapsii AS sapsii ON subject.stay_id = sapsii.stay_id
LEFT JOIN mimiciv_derived.oasis AS oasis ON subject.stay_id = oasis.stay_id
LEFT JOIN mimiciv_derived.charlson AS charlson ON subject.hadm_id = charlson.hadm_id
LEFT JOIN mimiciv_derived.apacheii AS apacheii ON subject.stay_id = apacheii.stay_id
ORDER BY subject.subject_id;
basic AS (
    WITH
    --Diagnosis included
    -- Cardiogenic shock
    includedDiagnose1 AS (
        SELECT DISTINCT hadm_id
        FROM mimiciv_hosp.diagnoses_icd
        WHERE icd_code IN ('78551')
    ),
    -- Cardiogenic shock
    includedDiagnose2 AS (
        SELECT DISTINCT hadm_id
        FROM mimiciv_hosp.diagnoses_icd
        WHERE icd_code IN ('R570')
```

```
    )
    SELECT
        DISTINCT ON (icu.subject_id)
        icu.subject_id,
        icu.stay_id,
        icu.hadm_id,
        icu.los_icu
    FROM mimiciv_derived.icustay_detail AS icu
    JOIN mimiciv_derived.age AS age ON age.hadm_id = icu.hadm_id
    WHERE
        TRUE
        AND age.age BETWEEN 18 AND 120
        AND icu.los_icu > 24.0/24.0
        AND icu.first_icu_stay = true
        AND icu.first_hosp_stay = true
        AND (icu.survival_day >= 1 OR icu.survival_day IS NULL)
        --Diagnosis included
        AND (
            -- Cardiogenic shock
            icu.hadm_id IN (SELECT hadm_id FROM includedDiagnose1)
            -- Cardiogenic shock
            OR icu.hadm_id IN (SELECT hadm_id FROM includedDiagnose2)
        )
)
--SELECT * FROM basic;


,
--   Absolute Lymphocyte Count
FIRST_Absolute_Lymphocyte_Count AS
    (SELECT DISTINCT ON (LAB.HADM_ID) LAB.HADM_ID,LAB.VALUENUM,
LAB.VALUEUOM,
    LAB.charttime AS Absolute_Lymphocyte_Count_charttime,
    LAB.storetime AS Absolute_Lymphocyte_Count_storetime
    FROM basic AS SUBJECT,
    MIMICIV_DERIVED.ICUSTAY_DETAIL AS ICU,
    MIMICIV_HOSP.LABEVENTS AS LAB
    WHERE LAB.ITEMID = 51133
    AND LAB.CHARTTIME >= ICU.ICU_INTIME
    AND SUBJECT.STAY_ID = ICU.STAY_ID
    AND SUBJECT.HADM_ID = LAB.HADM_ID
    ORDER BY LAB.HADM_ID,LAB.CHARTTIME)
,
-- 红细胞比容 Hematocrit 血常规
FIRST_Hematocrit AS
```

```
    (SELECT DISTINCT ON (LAB.HADM_ID) LAB.HADM_ID,LAB.VALUENUM,
LAB.VALUEUOM,
    LAB.charttime AS Hematocrit_charttime,
    LAB.storetime AS Hematocrit_storetime
    FROM basic AS SUBJECT,
    MIMICIV_DERIVED.ICUSTAY_DETAIL AS ICU,
    MIMICIV_HOSP.LABEVENTS AS LAB
    WHERE LAB.ITEMID = 51221
    AND LAB.CHARTTIME >= ICU.ICU_INTIME
    AND SUBJECT.STAY_ID = ICU.STAY_ID
    AND SUBJECT.HADM_ID = LAB.HADM_ID
    ORDER BY LAB.HADM_ID,LAB.CHARTTIME)
,
-- 血红蛋白 Hemoglobin 血常规
FIRST_Hemoglobin AS
    (SELECT DISTINCT ON (LAB.HADM_ID) LAB.HADM_ID,LAB.VALUENUM,
LAB.VALUEUOM,
    LAB.charttime AS Hemoglobin_charttime,
    LAB.storetime AS Hemoglobin_storetime
    FROM basic AS SUBJECT,
    MIMICIV_DERIVED.ICUSTAY_DETAIL AS ICU,
    MIMICIV_HOSP.LABEVENTS AS LAB
    WHERE LAB.ITEMID = 51222
    AND LAB.CHARTTIME >= ICU.ICU_INTIME
    AND SUBJECT.STAY_ID = ICU.STAY_ID
    AND SUBJECT.HADM_ID = LAB.HADM_ID
    ORDER BY LAB.HADM_ID,LAB.CHARTTIME)
,
-- 血小板计数 Platelet Count 血常规
FIRST_Platelet_Count AS
    (SELECT DISTINCT ON (LAB.HADM_ID) LAB.HADM_ID,LAB.VALUENUM,
LAB.VALUEUOM,
    LAB.charttime AS Platelet_Count_charttime,
    LAB.storetime AS Platelet_Count_storetime
    FROM basic AS SUBJECT,
    MIMICIV_DERIVED.ICUSTAY_DETAIL AS ICU,
    MIMICIV_HOSP.LABEVENTS AS LAB
    WHERE LAB.ITEMID = 51265
    AND LAB.CHARTTIME >= ICU.ICU_INTIME
    AND SUBJECT.STAY_ID = ICU.STAY_ID
    AND SUBJECT.HADM_ID = LAB.HADM_ID
    ORDER BY LAB.HADM_ID,LAB.CHARTTIME)
,
-- RDW RDW 血常规
```

```
FIRST_RDW AS
    (SELECT DISTINCT ON (LAB.HADM_ID) LAB.HADM_ID,LAB.VALUENUM,
LAB.VALUEUOM,
    LAB.charttime AS RDW_charttime,
    LAB.storetime AS RDW_storetime
    FROM basic AS SUBJECT,
    MIMICIV_DERIVED.ICUSTAY_DETAIL AS ICU,
    MIMICIV_HOSP.LABEVENTS AS LAB
    WHERE LAB.ITEMID = 51277
    AND LAB.CHARTTIME >= ICU.ICU_INTIME
    AND SUBJECT.STAY_ID = ICU.STAY_ID
    AND SUBJECT.HADM_ID = LAB.HADM_ID
    ORDER BY LAB.HADM_ID,LAB.CHARTTIME)
,
-- 红细胞  Red Blood Cells  血常规
FIRST_Red_Blood_Cells AS
    (SELECT DISTINCT ON (LAB.HADM_ID) LAB.HADM_ID,LAB.VALUENUM,
LAB.VALUEUOM,
    LAB.charttime AS Red_Blood_Cells_charttime,
    LAB.storetime AS Red_Blood_Cells_storetime
    FROM basic AS SUBJECT,
    MIMICIV_DERIVED.ICUSTAY_DETAIL AS ICU,
    MIMICIV_HOSP.LABEVENTS AS LAB
    WHERE LAB.ITEMID = 51279
    AND LAB.CHARTTIME >= ICU.ICU_INTIME
    AND SUBJECT.STAY_ID = ICU.STAY_ID
    AND SUBJECT.HADM_ID = LAB.HADM_ID
    ORDER BY LAB.HADM_ID,LAB.CHARTTIME)
,
-- 白细胞  White Blood Cells  血常规
FIRST_White_Blood_Cells AS
    (SELECT DISTINCT ON (LAB.HADM_ID) LAB.HADM_ID,LAB.VALUENUM,
LAB.VALUEUOM,
    LAB.charttime AS White_Blood_Cells_charttime,
    LAB.storetime AS White_Blood_Cells_storetime
    FROM basic AS SUBJECT,
    MIMICIV_DERIVED.ICUSTAY_DETAIL AS ICU,
    MIMICIV_HOSP.LABEVENTS AS LAB
    WHERE LAB.ITEMID = 51301
    AND LAB.CHARTTIME >= ICU.ICU_INTIME
    AND SUBJECT.STAY_ID = ICU.STAY_ID
    AND SUBJECT.HADM_ID = LAB.HADM_ID
    ORDER BY LAB.HADM_ID,LAB.CHARTTIME)
,
```

```
-- 绝对中性粒细胞计数 Absolute Neutrophil Count 血常规
FIRST_Absolute_Neutrophil_Count AS
    (SELECT DISTINCT ON (LAB.HADM_ID) LAB.HADM_ID,LAB.VALUENUM,
LAB.VALUEUOM,
    LAB.charttime AS Absolute_Neutrophil_Count_charttime,
    LAB.storetime AS Absolute_Neutrophil_Count_storetime
    FROM basic AS SUBJECT,
    MIMICIV_DERIVED.ICUSTAY_DETAIL AS ICU,
    MIMICIV_HOSP.LABEVENTS AS LAB
    WHERE LAB.ITEMID = 52075
    AND LAB.CHARTTIME >= ICU.ICU_INTIME
    AND SUBJECT.STAY_ID = ICU.STAY_ID
    AND SUBJECT.HADM_ID = LAB.HADM_ID
    ORDER BY LAB.HADM_ID,LAB.CHARTTIME)
,
-- %糖化血红蛋白 % Hemoglobin A1c 血生化
FIRST_Hemoglobin_A1c AS
    (SELECT DISTINCT ON (LAB.HADM_ID) LAB.HADM_ID,LAB.VALUENUM,
LAB.VALUEUOM,
    LAB.charttime AS Hemoglobin_A1c_charttime,
    LAB.storetime AS Hemoglobin_A1c_storetime
    FROM basic AS SUBJECT,
    MIMICIV_DERIVED.ICUSTAY_DETAIL AS ICU,
    MIMICIV_HOSP.LABEVENTS AS LAB
    WHERE LAB.ITEMID = 50852
    AND LAB.CHARTTIME >= ICU.ICU_INTIME
    AND SUBJECT.STAY_ID = ICU.STAY_ID
    AND SUBJECT.HADM_ID = LAB.HADM_ID
    ORDER BY LAB.HADM_ID,LAB.CHARTTIME)
,
-- 白蛋白 Albumin 血生化
FIRST_Albumin AS
    (SELECT DISTINCT ON (LAB.HADM_ID) LAB.HADM_ID,LAB.VALUENUM,
LAB.VALUEUOM,
    LAB.charttime AS Albumin_charttime,
    LAB.storetime AS Albumin_storetime
    FROM basic AS SUBJECT,
    MIMICIV_DERIVED.ICUSTAY_DETAIL AS ICU,
    MIMICIV_HOSP.LABEVENTS AS LAB
    WHERE LAB.ITEMID = 50862
    AND LAB.CHARTTIME >= ICU.ICU_INTIME
    AND SUBJECT.STAY_ID = ICU.STAY_ID
    AND SUBJECT.HADM_ID = LAB.HADM_ID
    ORDER BY LAB.HADM_ID,LAB.CHARTTIME)
```

```
,
-- 阴离子间隙 Anion Gap 血生化
FIRST_Anion_Gap AS
    (SELECT DISTINCT ON (LAB.HADM_ID) LAB.HADM_ID,LAB.VALUENUM,
LAB.VALUEUOM,
    LAB.charttime AS Anion_Gap_charttime,
    LAB.storetime AS Anion_Gap_storetime
    FROM basic AS SUBJECT,
    MIMICIV_DERIVED.ICUSTAY_DETAIL AS ICU,
    MIMICIV_HOSP.LABEVENTS AS LAB
    WHERE LAB.ITEMID = 50868
    AND LAB.CHARTTIME >= ICU.ICU_INTIME
    AND SUBJECT.STAY_ID = ICU.STAY_ID
    AND SUBJECT.HADM_ID = LAB.HADM_ID
    ORDER BY LAB.HADM_ID,LAB.CHARTTIME)
,
-- 总钙 Calcium, Total 血生化
FIRST_Calcium_Total AS
    (SELECT DISTINCT ON (LAB.HADM_ID) LAB.HADM_ID,LAB.VALUENUM,
LAB.VALUEUOM,
    LAB.charttime AS Calcium_Total_charttime,
    LAB.storetime AS Calcium_Total_storetime
    FROM basic AS SUBJECT,
    MIMICIV_DERIVED.ICUSTAY_DETAIL AS ICU,
    MIMICIV_HOSP.LABEVENTS AS LAB
    WHERE LAB.ITEMID = 50893
    AND LAB.CHARTTIME >= ICU.ICU_INTIME
    AND SUBJECT.STAY_ID = ICU.STAY_ID
    AND SUBJECT.HADM_ID = LAB.HADM_ID
    ORDER BY LAB.HADM_ID,LAB.CHARTTIME)
,
-- 氯浓度 Chloride 血生化
FIRST_Chloride AS
    (SELECT DISTINCT ON (LAB.HADM_ID) LAB.HADM_ID,LAB.VALUENUM,
LAB.VALUEUOM,
    LAB.charttime AS Chloride_charttime,
    LAB.storetime AS Chloride_storetime
    FROM basic AS SUBJECT,
    MIMICIV_DERIVED.ICUSTAY_DETAIL AS ICU,
    MIMICIV_HOSP.LABEVENTS AS LAB
    WHERE LAB.ITEMID = 50902
    AND LAB.CHARTTIME >= ICU.ICU_INTIME
    AND SUBJECT.STAY_ID = ICU.STAY_ID
    AND SUBJECT.HADM_ID = LAB.HADM_ID
```

```
    ORDER BY LAB.HADM_ID,LAB.CHARTTIME)
,
-- 球蛋白 Globulin 血生化
FIRST_Globulin AS
    (SELECT DISTINCT ON (LAB.HADM_ID) LAB.HADM_ID,LAB.VALUENUM,
LAB.VALUEUOM,
    LAB.charttime AS Globulin_charttime,
    LAB.storetime AS Globulin_storetime
    FROM basic AS SUBJECT,
    MIMICIV_DERIVED.ICUSTAY_DETAIL AS ICU,
    MIMICIV_HOSP.LABEVENTS AS LAB
    WHERE LAB.ITEMID = 50930
    AND LAB.CHARTTIME >= ICU.ICU_INTIME
    AND SUBJECT.STAY_ID = ICU.STAY_ID
    AND SUBJECT.HADM_ID = LAB.HADM_ID
    ORDER BY LAB.HADM_ID,LAB.CHARTTIME)
,
-- 葡萄糖 Glucose 血生化
FIRST_Glucose AS
    (SELECT DISTINCT ON (LAB.HADM_ID) LAB.HADM_ID,LAB.VALUENUM,
LAB.VALUEUOM,
    LAB.charttime AS Glucose_charttime,
    LAB.storetime AS Glucose_storetime
    FROM basic AS SUBJECT,
    MIMICIV_DERIVED.ICUSTAY_DETAIL AS ICU,
    MIMICIV_HOSP.LABEVENTS AS LAB
    WHERE LAB.ITEMID = 50931
    AND LAB.CHARTTIME >= ICU.ICU_INTIME
    AND SUBJECT.STAY_ID = ICU.STAY_ID
    AND SUBJECT.HADM_ID = LAB.HADM_ID
    ORDER BY LAB.HADM_ID,LAB.CHARTTIME)
,
-- 钾 Potassium 血生化
FIRST_Potassium AS
    (SELECT DISTINCT ON (LAB.HADM_ID) LAB.HADM_ID,LAB.VALUENUM,
LAB.VALUEUOM,
    LAB.charttime AS Potassium_charttime,
    LAB.storetime AS Potassium_storetime
    FROM basic AS SUBJECT,
    MIMICIV_DERIVED.ICUSTAY_DETAIL AS ICU,
    MIMICIV_HOSP.LABEVENTS AS LAB
    WHERE LAB.ITEMID = 50971
    AND LAB.CHARTTIME >= ICU.ICU_INTIME
    AND SUBJECT.STAY_ID = ICU.STAY_ID
```

```sql
    AND SUBJECT.HADM_ID = LAB.HADM_ID
    ORDER BY LAB.HADM_ID,LAB.CHARTTIME)
,
-- 总蛋白  Protein, Total  血生化
FIRST_Protein_Total AS
    (SELECT DISTINCT ON (LAB.HADM_ID) LAB.HADM_ID,LAB.VALUENUM,
LAB.VALUEUOM,
    LAB.charttime AS Protein_Total_charttime,
    LAB.storetime AS Protein_Total_storetime
    FROM basic AS SUBJECT,
    MIMICIV_DERIVED.ICUSTAY_DETAIL AS ICU,
    MIMICIV_HOSP.LABEVENTS AS LAB
    WHERE LAB.ITEMID = 50976
    AND LAB.CHARTTIME >= ICU.ICU_INTIME
    AND SUBJECT.STAY_ID = ICU.STAY_ID
    AND SUBJECT.HADM_ID = LAB.HADM_ID
    ORDER BY LAB.HADM_ID,LAB.CHARTTIME)
,
-- 钠  Sodium  血生化
FIRST_Sodium AS
    (SELECT DISTINCT ON (LAB.HADM_ID) LAB.HADM_ID,LAB.VALUENUM,
LAB.VALUEUOM,
    LAB.charttime AS Sodium_charttime,
    LAB.storetime AS Sodium_storetime
    FROM basic AS SUBJECT,
    MIMICIV_DERIVED.ICUSTAY_DETAIL AS ICU,
    MIMICIV_HOSP.LABEVENTS AS LAB
    WHERE LAB.ITEMID = 50983
    AND LAB.CHARTTIME >= ICU.ICU_INTIME
    AND SUBJECT.STAY_ID = ICU.STAY_ID
    AND SUBJECT.HADM_ID = LAB.HADM_ID
    ORDER BY LAB.HADM_ID,LAB.CHARTTIME)
,
-- 计算总二氧化碳  Calculated Total CO2  血气
FIRST_Calculated_Total_CO2 AS
    (SELECT DISTINCT ON (LAB.HADM_ID) LAB.HADM_ID,LAB.VALUENUM,
LAB.VALUEUOM,
    LAB.charttime AS Calculated_Total_CO2_charttime,
    LAB.storetime AS Calculated_Total_CO2_storetime
    FROM basic AS SUBJECT,
    MIMICIV_DERIVED.ICUSTAY_DETAIL AS ICU,
    MIMICIV_HOSP.LABEVENTS AS LAB
    WHERE LAB.ITEMID = 50804
    AND LAB.CHARTTIME >= ICU.ICU_INTIME
```

```sql
    AND SUBJECT.STAY_ID = ICU.STAY_ID
    AND SUBJECT.HADM_ID = LAB.HADM_ID
    ORDER BY LAB.HADM_ID,LAB.CHARTTIME)
,
-- 游离钙 Free Calcium 血气
FIRST_Free_Calcium AS
    (SELECT DISTINCT ON (LAB.HADM_ID) LAB.HADM_ID,LAB.VALUENUM,
LAB.VALUEUOM,
    LAB.charttime AS Free_Calcium_charttime,
    LAB.storetime AS Free_Calcium_storetime
    FROM basic AS SUBJECT,
    MIMICIV_DERIVED.ICUSTAY_DETAIL AS ICU,
    MIMICIV_HOSP.LABEVENTS AS LAB
    WHERE LAB.ITEMID = 50808
    AND LAB.CHARTTIME >= ICU.ICU_INTIME
    AND SUBJECT.STAY_ID = ICU.STAY_ID
    AND SUBJECT.HADM_ID = LAB.HADM_ID
    ORDER BY LAB.HADM_ID,LAB.CHARTTIME)
,
-- 乳酸 Lactate 血气
FIRST_Lactate AS
    (SELECT DISTINCT ON (LAB.HADM_ID) LAB.HADM_ID,LAB.VALUENUM,
LAB.VALUEUOM,
    LAB.charttime AS Lactate_charttime,
    LAB.storetime AS Lactate_storetime
    FROM basic AS SUBJECT,
    MIMICIV_DERIVED.ICUSTAY_DETAIL AS ICU,
    MIMICIV_HOSP.LABEVENTS AS LAB
    WHERE LAB.ITEMID = 50813
    AND LAB.CHARTTIME >= ICU.ICU_INTIME
    AND SUBJECT.STAY_ID = ICU.STAY_ID
    AND SUBJECT.HADM_ID = LAB.HADM_ID
    ORDER BY LAB.HADM_ID,LAB.CHARTTIME)
,
-- 二氧化碳分压 pCO2 血气
FIRST_pCO2 AS
    (SELECT DISTINCT ON (LAB.HADM_ID) LAB.HADM_ID,LAB.VALUENUM,
LAB.VALUEUOM,
    LAB.charttime AS pCO2_charttime,
    LAB.storetime AS pCO2_storetime
    FROM basic AS SUBJECT,
    MIMICIV_DERIVED.ICUSTAY_DETAIL AS ICU,
    MIMICIV_HOSP.LABEVENTS AS LAB
    WHERE LAB.ITEMID = 50818
```

```
    AND LAB.CHARTTIME >= ICU.ICU_INTIME
    AND SUBJECT.STAY_ID = ICU.STAY_ID
    AND SUBJECT.HADM_ID = LAB.HADM_ID
    ORDER BY LAB.HADM_ID,LAB.CHARTTIME)
,
-- pH 值  pH  血气
FIRST_pH AS
    (SELECT DISTINCT ON (LAB.HADM_ID) LAB.HADM_ID,LAB.VALUENUM,
LAB.VALUEUOM,
    LAB.charttime AS pH_charttime,
    LAB.storetime AS pH_storetime
    FROM basic AS SUBJECT,
    MIMICIV_DERIVED.ICUSTAY_DETAIL AS ICU,
    MIMICIV_HOSP.LABEVENTS AS LAB
    WHERE LAB.ITEMID = 50820
    AND LAB.CHARTTIME >= ICU.ICU_INTIME
    AND SUBJECT.STAY_ID = ICU.STAY_ID
    AND SUBJECT.HADM_ID = LAB.HADM_ID
    ORDER BY LAB.HADM_ID,LAB.CHARTTIME)
,
--  氧分压  pO2  血气
FIRST_pO2 AS
    (SELECT DISTINCT ON (LAB.HADM_ID) LAB.HADM_ID,LAB.VALUENUM,
LAB.VALUEUOM,
    LAB.charttime AS pO2_charttime,
    LAB.storetime AS pO2_storetime
    FROM basic AS SUBJECT,
    MIMICIV_DERIVED.ICUSTAY_DETAIL AS ICU,
    MIMICIV_HOSP.LABEVENTS AS LAB
    WHERE LAB.ITEMID = 50821
    AND LAB.CHARTTIME >= ICU.ICU_INTIME
    AND SUBJECT.STAY_ID = ICU.STAY_ID
    AND SUBJECT.HADM_ID = LAB.HADM_ID
    ORDER BY LAB.HADM_ID,LAB.CHARTTIME)
,
-- D-二聚体  D-Dimer  凝血
FIRST_DDimer AS
    (SELECT DISTINCT ON (LAB.HADM_ID) LAB.HADM_ID,LAB.VALUENUM,
LAB.VALUEUOM,
    LAB.charttime AS DDimer_charttime,
    LAB.storetime AS DDimer_storetime
    FROM basic AS SUBJECT,
    MIMICIV_DERIVED.ICUSTAY_DETAIL AS ICU,
    MIMICIV_HOSP.LABEVENTS AS LAB
```

```
    WHERE LAB.ITEMID = 51196
    AND LAB.CHARTTIME >= ICU.ICU_INTIME
    AND SUBJECT.STAY_ID = ICU.STAY_ID
    AND SUBJECT.HADM_ID = LAB.HADM_ID
    ORDER BY LAB.HADM_ID,LAB.CHARTTIME)
,
-- 功能性纤维蛋白原 Fibrinogen, Functional 凝血
FIRST_Fibrinogen_Functional AS
    (SELECT DISTINCT ON (LAB.HADM_ID) LAB.HADM_ID,LAB.VALUENUM,
LAB.VALUEUOM,
    LAB.charttime AS Fibrinogen_Functional_charttime,
    LAB.storetime AS Fibrinogen_Functional_storetime
    FROM basic AS SUBJECT,
    MIMICIV_DERIVED.ICUSTAY_DETAIL AS ICU,
    MIMICIV_HOSP.LABEVENTS AS LAB
    WHERE LAB.ITEMID = 51214
    AND LAB.CHARTTIME >= ICU.ICU_INTIME
    AND SUBJECT.STAY_ID = ICU.STAY_ID
    AND SUBJECT.HADM_ID = LAB.HADM_ID
    ORDER BY LAB.HADM_ID,LAB.CHARTTIME)
,
-- 凝血酶原时间国际标准化比值(PT-INR) INR(PT) 凝血
FIRST_INRPT AS
    (SELECT DISTINCT ON (LAB.HADM_ID) LAB.HADM_ID,LAB.VALUENUM,
LAB.VALUEUOM,
    LAB.charttime AS INRPT_charttime,
    LAB.storetime AS INRPT_storetime
    FROM basic AS SUBJECT,
    MIMICIV_DERIVED.ICUSTAY_DETAIL AS ICU,
    MIMICIV_HOSP.LABEVENTS AS LAB
    WHERE LAB.ITEMID = 51237
    AND LAB.CHARTTIME >= ICU.ICU_INTIME
    AND SUBJECT.STAY_ID = ICU.STAY_ID
    AND SUBJECT.HADM_ID = LAB.HADM_ID
    ORDER BY LAB.HADM_ID,LAB.CHARTTIME)
,
-- PT PT 凝血
FIRST_PT AS
    (SELECT DISTINCT ON (LAB.HADM_ID) LAB.HADM_ID,LAB.VALUENUM,
LAB.VALUEUOM,
    LAB.charttime AS PT_charttime,
    LAB.storetime AS PT_storetime
    FROM basic AS SUBJECT,
    MIMICIV_DERIVED.ICUSTAY_DETAIL AS ICU,
```

```
    MIMICIV_HOSP.LABEVENTS AS LAB
    WHERE LAB.ITEMID = 51274
    AND LAB.CHARTTIME >= ICU.ICU_INTIME
    AND SUBJECT.STAY_ID = ICU.STAY_ID
    AND SUBJECT.HADM_ID = LAB.HADM_ID
    ORDER BY LAB.HADM_ID,LAB.CHARTTIME)
,
-- PTT PTT 凝血
FIRST_PTT AS
    (SELECT DISTINCT ON (LAB.HADM_ID) LAB.HADM_ID,LAB.VALUENUM,
LAB.VALUEUOM,
    LAB.charttime AS PTT_charttime,
    LAB.storetime AS PTT_storetime
    FROM basic AS SUBJECT,
    MIMICIV_DERIVED.ICUSTAY_DETAIL AS ICU,
    MIMICIV_HOSP.LABEVENTS AS LAB
    WHERE LAB.ITEMID = 51275
    AND LAB.CHARTTIME >= ICU.ICU_INTIME
    AND SUBJECT.STAY_ID = ICU.STAY_ID
    AND SUBJECT.HADM_ID = LAB.HADM_ID
    ORDER BY LAB.HADM_ID,LAB.CHARTTIME)
,
-- 凝血酶 Thrombin 凝血
FIRST_Thrombin AS
    (SELECT DISTINCT ON (LAB.HADM_ID) LAB.HADM_ID,LAB.VALUENUM,
LAB.VALUEUOM,
    LAB.charttime AS Thrombin_charttime,
    LAB.storetime AS Thrombin_storetime
    FROM basic AS SUBJECT,
    MIMICIV_DERIVED.ICUSTAY_DETAIL AS ICU,
    MIMICIV_HOSP.LABEVENTS AS LAB
    WHERE LAB.ITEMID = 51297
    AND LAB.CHARTTIME >= ICU.ICU_INTIME
    AND SUBJECT.STAY_ID = ICU.STAY_ID
    AND SUBJECT.HADM_ID = LAB.HADM_ID
    ORDER BY LAB.HADM_ID,LAB.CHARTTIME)
,
-- 高密度脂蛋白胆固醇 Cholesterol, HDL 血脂
FIRST_Cholesterol_HDL AS
    (SELECT DISTINCT ON (LAB.HADM_ID) LAB.HADM_ID,LAB.VALUENUM,
LAB.VALUEUOM,
    LAB.charttime AS Cholesterol_HDL_charttime,
    LAB.storetime AS Cholesterol_HDL_storetime
    FROM basic AS SUBJECT,
```

```
    MIMICIV_DERIVED.ICUSTAY_DETAIL AS ICU,
    MIMICIV_HOSP.LABEVENTS AS LAB
    WHERE LAB.ITEMID = 50904
    AND LAB.CHARTTIME >= ICU.ICU_INTIME
    AND SUBJECT.STAY_ID = ICU.STAY_ID
    AND SUBJECT.HADM_ID = LAB.HADM_ID
    ORDER BY LAB.HADM_ID,LAB.CHARTTIME)
,
-- 计算低密度脂蛋白胆固醇 Cholesterol, LDL, Calculated 血脂
FIRST_Cholesterol_LDL_Calculated AS
    (SELECT DISTINCT ON (LAB.HADM_ID) LAB.HADM_ID,LAB.VALUENUM,
LAB.VALUEUOM,
    LAB.charttime AS Cholesterol_LDL_Calculated_charttime,
    LAB.storetime AS Cholesterol_LDL_Calculated_storetime
    FROM basic AS SUBJECT,
    MIMICIV_DERIVED.ICUSTAY_DETAIL AS ICU,
    MIMICIV_HOSP.LABEVENTS AS LAB
    WHERE LAB.ITEMID = 50905
    AND LAB.CHARTTIME >= ICU.ICU_INTIME
    AND SUBJECT.STAY_ID = ICU.STAY_ID
    AND SUBJECT.HADM_ID = LAB.HADM_ID
    ORDER BY LAB.HADM_ID,LAB.CHARTTIME)
,
-- 总胆固醇 Cholesterol, Total 血脂
FIRST_Cholesterol_Total AS
    (SELECT DISTINCT ON (LAB.HADM_ID) LAB.HADM_ID,LAB.VALUENUM,
LAB.VALUEUOM,
    LAB.charttime AS Cholesterol_Total_charttime,
    LAB.storetime AS Cholesterol_Total_storetime
    FROM basic AS SUBJECT,
    MIMICIV_DERIVED.ICUSTAY_DETAIL AS ICU,
    MIMICIV_HOSP.LABEVENTS AS LAB
    WHERE LAB.ITEMID = 50907
    AND LAB.CHARTTIME >= ICU.ICU_INTIME
    AND SUBJECT.STAY_ID = ICU.STAY_ID
    AND SUBJECT.HADM_ID = LAB.HADM_ID
    ORDER BY LAB.HADM_ID,LAB.CHARTTIME)
,
-- 甘油三酯 Triglycerides 血脂
FIRST_Triglycerides AS
    (SELECT DISTINCT ON (LAB.HADM_ID) LAB.HADM_ID,LAB.VALUENUM,
LAB.VALUEUOM,
    LAB.charttime AS Triglycerides_charttime,
    LAB.storetime AS Triglycerides_storetime
```

```
    FROM basic AS SUBJECT,
    MIMICIV_DERIVED.ICUSTAY_DETAIL AS ICU,
    MIMICIV_HOSP.LABEVENTS AS LAB
    WHERE LAB.ITEMID = 51000
    AND LAB.CHARTTIME >= ICU.ICU_INTIME
    AND SUBJECT.STAY_ID = ICU.STAY_ID
    AND SUBJECT.HADM_ID = LAB.HADM_ID
    ORDER BY LAB.HADM_ID,LAB.CHARTTIME)
,
-- 丙氨酸氨基转移酶（ALT） Alanine Aminotransferase (ALT) 肝肾功能
FIRST_Alanine_Aminotransferase_ALT AS
    (SELECT DISTINCT ON (LAB.HADM_ID) LAB.HADM_ID,LAB.VALUENUM,
LAB.VALUEUOM,
    LAB.charttime AS Alanine_Aminotransferase_ALT_charttime,
    LAB.storetime AS Alanine_Aminotransferase_ALT_storetime
    FROM basic AS SUBJECT,
    MIMICIV_DERIVED.ICUSTAY_DETAIL AS ICU,
    MIMICIV_HOSP.LABEVENTS AS LAB
    WHERE LAB.ITEMID = 50861
    AND LAB.CHARTTIME >= ICU.ICU_INTIME
    AND SUBJECT.STAY_ID = ICU.STAY_ID
    AND SUBJECT.HADM_ID = LAB.HADM_ID
    ORDER BY LAB.HADM_ID,LAB.CHARTTIME)
,
-- 门冬氨酸氨基转移酶（AST） Asparate Aminotransferase (AST) 肝肾功能
FIRST_Asparate_Aminotransferase_AST AS
    (SELECT DISTINCT ON (LAB.HADM_ID) LAB.HADM_ID,LAB.VALUENUM,
LAB.VALUEUOM,
    LAB.charttime AS Asparate_Aminotransferase_AST_charttime,
    LAB.storetime AS Asparate_Aminotransferase_AST_storetime
    FROM basic AS SUBJECT,
    MIMICIV_DERIVED.ICUSTAY_DETAIL AS ICU,
    MIMICIV_HOSP.LABEVENTS AS LAB
    WHERE LAB.ITEMID = 50878
    AND LAB.CHARTTIME >= ICU.ICU_INTIME
    AND SUBJECT.STAY_ID = ICU.STAY_ID
    AND SUBJECT.HADM_ID = LAB.HADM_ID
    ORDER BY LAB.HADM_ID,LAB.CHARTTIME)
,
-- 直接胆红素 Bilirubin, Direct 肝肾功能
FIRST_Bilirubin_Direct AS
    (SELECT DISTINCT ON (LAB.HADM_ID) LAB.HADM_ID,LAB.VALUENUM,
LAB.VALUEUOM,
    LAB.charttime AS Bilirubin_Direct_charttime,
```

```
    LAB.storetime AS Bilirubin_Direct_storetime
    FROM basic AS SUBJECT,
    MIMICIV_DERIVED.ICUSTAY_DETAIL AS ICU,
    MIMICIV_HOSP.LABEVENTS AS LAB
    WHERE LAB.ITEMID = 50883
    AND LAB.CHARTTIME >= ICU.ICU_INTIME
    AND SUBJECT.STAY_ID = ICU.STAY_ID
    AND SUBJECT.HADM_ID = LAB.HADM_ID
    ORDER BY LAB.HADM_ID,LAB.CHARTTIME)
,
-- 间接胆红素 Bilirubin, Indirect 肝肾功能
FIRST_Bilirubin_Indirect AS
    (SELECT DISTINCT ON (LAB.HADM_ID) LAB.HADM_ID,LAB.VALUENUM,
LAB.VALUEUOM,
    LAB.charttime AS Bilirubin_Indirect_charttime,
    LAB.storetime AS Bilirubin_Indirect_storetime
    FROM basic AS SUBJECT,
    MIMICIV_DERIVED.ICUSTAY_DETAIL AS ICU,
    MIMICIV_HOSP.LABEVENTS AS LAB
    WHERE LAB.ITEMID = 50884
    AND LAB.CHARTTIME >= ICU.ICU_INTIME
    AND SUBJECT.STAY_ID = ICU.STAY_ID
    AND SUBJECT.HADM_ID = LAB.HADM_ID
    ORDER BY LAB.HADM_ID,LAB.CHARTTIME)
,
-- 总胆红素 Bilirubin, Total 肝肾功能
FIRST_Bilirubin_Total AS
    (SELECT DISTINCT ON (LAB.HADM_ID) LAB.HADM_ID,LAB.VALUENUM,
LAB.VALUEUOM,
    LAB.charttime AS Bilirubin_Total_charttime,
    LAB.storetime AS Bilirubin_Total_storetime
    FROM basic AS SUBJECT,
    MIMICIV_DERIVED.ICUSTAY_DETAIL AS ICU,
    MIMICIV_HOSP.LABEVENTS AS LAB
    WHERE LAB.ITEMID = 50885
    AND LAB.CHARTTIME >= ICU.ICU_INTIME
    AND SUBJECT.STAY_ID = ICU.STAY_ID
    AND SUBJECT.HADM_ID = LAB.HADM_ID
    ORDER BY LAB.HADM_ID,LAB.CHARTTIME)
,
-- 肌酐 Creatinine 肝肾功能
FIRST_Creatinine AS
    (SELECT DISTINCT ON (LAB.HADM_ID) LAB.HADM_ID,LAB.VALUENUM,
LAB.VALUEUOM,
```

```
    LAB.charttime AS Creatinine_charttime,
    LAB.storetime AS Creatinine_storetime
    FROM basic AS SUBJECT,
    MIMICIV_DERIVED.ICUSTAY_DETAIL AS ICU,
    MIMICIV_HOSP.LABEVENTS AS LAB
    WHERE LAB.ITEMID = 50912
    AND LAB.CHARTTIME >= ICU.ICU_INTIME
    AND SUBJECT.STAY_ID = ICU.STAY_ID
    AND SUBJECT.HADM_ID = LAB.HADM_ID
    ORDER BY LAB.HADM_ID,LAB.CHARTTIME)
,
-- 尿素氮 Urea Nitrogen 肝肾功能
FIRST_Urea_Nitrogen AS
    (SELECT DISTINCT ON (LAB.HADM_ID) LAB.HADM_ID,LAB.VALUENUM,
LAB.VALUEUOM,
    LAB.charttime AS Urea_Nitrogen_charttime,
    LAB.storetime AS Urea_Nitrogen_storetime
    FROM basic AS SUBJECT,
    MIMICIV_DERIVED.ICUSTAY_DETAIL AS ICU,
    MIMICIV_HOSP.LABEVENTS AS LAB
    WHERE LAB.ITEMID = 51006
    AND LAB.CHARTTIME >= ICU.ICU_INTIME
    AND SUBJECT.STAY_ID = ICU.STAY_ID
    AND SUBJECT.HADM_ID = LAB.HADM_ID
    ORDER BY LAB.HADM_ID,LAB.CHARTTIME)
,
-- 尿酸 Uric Acid 肝肾功能
FIRST_Uric_Acid AS
    (SELECT DISTINCT ON (LAB.HADM_ID) LAB.HADM_ID,LAB.VALUENUM,
LAB.VALUEUOM,
    LAB.charttime AS Uric_Acid_charttime,
    LAB.storetime AS Uric_Acid_storetime
    FROM basic AS SUBJECT,
    MIMICIV_DERIVED.ICUSTAY_DETAIL AS ICU,
    MIMICIV_HOSP.LABEVENTS AS LAB
    WHERE LAB.ITEMID = 51007
    AND LAB.CHARTTIME >= ICU.ICU_INTIME
    AND SUBJECT.STAY_ID = ICU.STAY_ID
    AND SUBJECT.HADM_ID = LAB.HADM_ID
    ORDER BY LAB.HADM_ID,LAB.CHARTTIME)
,
-- 肌酸激酶（CK） Creatine Kinase (CK) 心肌酶谱
FIRST_Creatine_Kinase_CK AS
```

```sql
    (SELECT DISTINCT ON (LAB.HADM_ID) LAB.HADM_ID,LAB.VALUENUM,
LAB.VALUEUOM,
    LAB.charttime AS Creatine_Kinase_CK_charttime,
    LAB.storetime AS Creatine_Kinase_CK_storetime
    FROM basic AS SUBJECT,
    MIMICIV_DERIVED.ICUSTAY_DETAIL AS ICU,
    MIMICIV_HOSP.LABEVENTS AS LAB
    WHERE LAB.ITEMID = 50910
    AND LAB.CHARTTIME >= ICU.ICU_INTIME
    AND SUBJECT.STAY_ID = ICU.STAY_ID
    AND SUBJECT.HADM_ID = LAB.HADM_ID
    ORDER BY LAB.HADM_ID,LAB.CHARTTIME)
,
-- 肌酸激酶，MB 亚型  Creatine Kinase, MB Isoenzyme  心肌酶谱
FIRST_Creatine_Kinase_MB_Isoenzyme AS
    (SELECT DISTINCT ON (LAB.HADM_ID) LAB.HADM_ID,LAB.VALUENUM,
LAB.VALUEUOM,
    LAB.charttime AS Creatine_Kinase_MB_Isoenzyme_charttime,
    LAB.storetime AS Creatine_Kinase_MB_Isoenzyme_storetime
    FROM basic AS SUBJECT,
    MIMICIV_DERIVED.ICUSTAY_DETAIL AS ICU,
    MIMICIV_HOSP.LABEVENTS AS LAB
    WHERE LAB.ITEMID = 50911
    AND LAB.CHARTTIME >= ICU.ICU_INTIME
    AND SUBJECT.STAY_ID = ICU.STAY_ID
    AND SUBJECT.HADM_ID = LAB.HADM_ID
    ORDER BY LAB.HADM_ID,LAB.CHARTTIME)
,
-- 乳酸脱氢酶（LD）  Lactate Dehydrogenase (LD)  心肌酶谱
FIRST_Lactate_Dehydrogenase_LD AS
    (SELECT DISTINCT ON (LAB.HADM_ID) LAB.HADM_ID,LAB.VALUENUM,
LAB.VALUEUOM,
    LAB.charttime AS Lactate_Dehydrogenase_LD_charttime,
    LAB.storetime AS Lactate_Dehydrogenase_LD_storetime
    FROM basic AS SUBJECT,
    MIMICIV_DERIVED.ICUSTAY_DETAIL AS ICU,
    MIMICIV_HOSP.LABEVENTS AS LAB
    WHERE LAB.ITEMID = 50954
    AND LAB.CHARTTIME >= ICU.ICU_INTIME
    AND SUBJECT.STAY_ID = ICU.STAY_ID
    AND SUBJECT.HADM_ID = LAB.HADM_ID
    ORDER BY LAB.HADM_ID,LAB.CHARTTIME)
,
-- NT 前体 B 型钠尿肽  NTproBNP  心肌酶谱
```

```
FIRST_NTproBNP AS
    (SELECT DISTINCT ON (LAB.HADM_ID) LAB.HADM_ID,LAB.VALUENUM,
LAB.VALUEUOM,
    LAB.charttime AS NTproBNP_charttime,
    LAB.storetime AS NTproBNP_storetime
    FROM basic AS SUBJECT,
    MIMICIV_DERIVED.ICUSTAY_DETAIL AS ICU,
    MIMICIV_HOSP.LABEVENTS AS LAB
    WHERE LAB.ITEMID = 50963
    AND LAB.CHARTTIME >= ICU.ICU_INTIME
    AND SUBJECT.STAY_ID = ICU.STAY_ID
    AND SUBJECT.HADM_ID = LAB.HADM_ID
    ORDER BY LAB.HADM_ID,LAB.CHARTTIME)
,
-- 肌钙蛋白 T Troponin T  心肌酶谱
FIRST_Troponin_T AS
    (SELECT DISTINCT ON (LAB.HADM_ID) LAB.HADM_ID,LAB.VALUENUM,
LAB.VALUEUOM,
    LAB.charttime AS Troponin_T_charttime,
    LAB.storetime AS Troponin_T_storetime
    FROM basic AS SUBJECT,
    MIMICIV_DERIVED.ICUSTAY_DETAIL AS ICU,
    MIMICIV_HOSP.LABEVENTS AS LAB
    WHERE LAB.ITEMID = 51003
    AND LAB.CHARTTIME >= ICU.ICU_INTIME
    AND SUBJECT.STAY_ID = ICU.STAY_ID
    AND SUBJECT.HADM_ID = LAB.HADM_ID
    ORDER BY LAB.HADM_ID,LAB.CHARTTIME)
,
-- 肌钙蛋白 I Troponin I  心肌酶谱
FIRST_Troponin_I AS
    (SELECT DISTINCT ON (LAB.HADM_ID) LAB.HADM_ID,LAB.VALUENUM,
LAB.VALUEUOM,
    LAB.charttime AS Troponin_I_charttime,
    LAB.storetime AS Troponin_I_storetime
    FROM basic AS SUBJECT,
    MIMICIV_DERIVED.ICUSTAY_DETAIL AS ICU,
    MIMICIV_HOSP.LABEVENTS AS LAB
    WHERE LAB.ITEMID = 52642
    AND LAB.CHARTTIME >= ICU.ICU_INTIME
    AND SUBJECT.STAY_ID = ICU.STAY_ID
    AND SUBJECT.HADM_ID = LAB.HADM_ID
    ORDER BY LAB.HADM_ID,LAB.CHARTTIME)
,
```

```
-- 镁离子 Magnesium
FIRST_Magnesium AS
    (SELECT DISTINCT ON (LAB.HADM_ID) LAB.HADM_ID,LAB.VALUENUM,
LAB.VALUEUOM,
    LAB.charttime AS Magnesium_charttime,
    LAB.storetime AS Magnesium_storetime
    FROM basic AS SUBJECT,
    MIMICIV_DERIVED.ICUSTAY_DETAIL AS ICU,
    MIMICIV_HOSP.LABEVENTS AS LAB
    WHERE LAB.ITEMID = 50960
    AND LAB.CHARTTIME >= ICU.ICU_INTIME
    AND SUBJECT.STAY_ID = ICU.STAY_ID
    AND SUBJECT.HADM_ID = LAB.HADM_ID
    ORDER BY LAB.HADM_ID,LAB.CHARTTIME)
,
-- 磷酸盐 Phosphate
FIRST_Phosphate AS
    (SELECT DISTINCT ON (LAB.HADM_ID) LAB.HADM_ID,LAB.VALUENUM,
LAB.VALUEUOM,
    LAB.charttime AS Phosphate_charttime,
    LAB.storetime AS Phosphate_storetime
    FROM basic AS SUBJECT,
    MIMICIV_DERIVED.ICUSTAY_DETAIL AS ICU,
    MIMICIV_HOSP.LABEVENTS AS LAB
    WHERE LAB.ITEMID = 50970
    AND LAB.CHARTTIME >= ICU.ICU_INTIME
    AND SUBJECT.STAY_ID = ICU.STAY_ID
    AND SUBJECT.HADM_ID = LAB.HADM_ID
    ORDER BY LAB.HADM_ID,LAB.CHARTTIME)
,
-- 淋巴细胞 Lymphocytes
FIRST_Lymphocytes AS
    (SELECT DISTINCT ON (LAB.HADM_ID) LAB.HADM_ID,LAB.VALUENUM,
LAB.VALUEUOM,
    LAB.charttime AS Lymphocytes_charttime,
    LAB.storetime AS Lymphocytes_storetime
    FROM basic AS SUBJECT,
    MIMICIV_DERIVED.ICUSTAY_DETAIL AS ICU,
    MIMICIV_HOSP.LABEVENTS AS LAB
    WHERE LAB.ITEMID = 51244
    AND LAB.CHARTTIME >= ICU.ICU_INTIME
    AND SUBJECT.STAY_ID = ICU.STAY_ID
    AND SUBJECT.HADM_ID = LAB.HADM_ID
    ORDER BY LAB.HADM_ID,LAB.CHARTTIME)
```

```
,
-- 单核细胞  Monocytes
FIRST_Monocytes AS
    (SELECT DISTINCT ON (LAB.HADM_ID) LAB.HADM_ID,LAB.VALUENUM,
LAB.VALUEUOM,
    LAB.charttime AS Monocytes_charttime,
    LAB.storetime AS Monocytes_storetime
    FROM basic AS SUBJECT,
    MIMICIV_DERIVED.ICUSTAY_DETAIL AS ICU,
    MIMICIV_HOSP.LABEVENTS AS LAB
    WHERE LAB.ITEMID = 51254
    AND LAB.CHARTTIME >= ICU.ICU_INTIME
    AND SUBJECT.STAY_ID = ICU.STAY_ID
    AND SUBJECT.HADM_ID = LAB.HADM_ID
    ORDER BY LAB.HADM_ID,LAB.CHARTTIME)
,
-- 中性粒细胞  Neutrophils
FIRST_Neutrophils AS
    (SELECT DISTINCT ON (LAB.HADM_ID) LAB.HADM_ID,LAB.VALUENUM,
LAB.VALUEUOM,
    LAB.charttime AS Neutrophils_charttime,
    LAB.storetime AS Neutrophils_storetime
    FROM basic AS SUBJECT,
    MIMICIV_DERIVED.ICUSTAY_DETAIL AS ICU,
    MIMICIV_HOSP.LABEVENTS AS LAB
    WHERE LAB.ITEMID = 51256
    AND LAB.CHARTTIME >= ICU.ICU_INTIME
    AND SUBJECT.STAY_ID = ICU.STAY_ID
    AND SUBJECT.HADM_ID = LAB.HADM_ID
    ORDER BY LAB.HADM_ID,LAB.CHARTTIME)
,
-- 绝对单核细胞计数  Absolute Monocyte Count
FIRST_Absolute_Monocyte_Count AS
    (SELECT DISTINCT ON (LAB.HADM_ID) LAB.HADM_ID,LAB.VALUENUM,
LAB.VALUEUOM,
    LAB.charttime AS Absolute_Monocyte_Count_charttime,
    LAB.storetime AS Absolute_Monocyte_Count_storetime
    FROM basic AS SUBJECT,
    MIMICIV_DERIVED.ICUSTAY_DETAIL AS ICU,
    MIMICIV_HOSP.LABEVENTS AS LAB
    WHERE LAB.ITEMID = 52074
    AND LAB.CHARTTIME >= ICU.ICU_INTIME
    AND SUBJECT.STAY_ID = ICU.STAY_ID
    AND SUBJECT.HADM_ID = LAB.HADM_ID
```

```
    ORDER BY LAB.HADM_ID,LAB.CHARTTIME)

 SELECT
    DISTINCT(SUBJECT.SUBJECT_ID),
    SUBJECT.STAY_ID,
    SUBJECT.HADM_ID,
    ROUND(FIRST_Absolute_Lymphocyte_Count.VALUENUM::numeric,2) AS
FIRST_Absolute_Lymphocyte_Count,
    FIRST_Absolute_Lymphocyte_Count.VALUEUOM AS
FIRST_Absolute_Lymphocyte_Count_UOM,
    FIRST_Absolute_Lymphocyte_Count.Absolute_Lymphocyte_Count_charttime,
    FIRST_Absolute_Lymphocyte_Count.Absolute_Lymphocyte_Count_storetime,
    ROUND(FIRST_Hematocrit.VALUENUM::numeric,2) AS FIRST_Hematocrit,
    FIRST_Hematocrit.VALUEUOM AS FIRST_Hematocrit_UOM,
    FIRST_Hematocrit.Hematocrit_charttime,
    FIRST_Hematocrit.Hematocrit_storetime,
    ROUND(FIRST_Hemoglobin.VALUENUM::numeric,2) AS FIRST_Hemoglobin,
    FIRST_Hemoglobin.VALUEUOM AS FIRST_Hemoglobin_UOM,
    FIRST_Hemoglobin.Hemoglobin_charttime,
    FIRST_Hemoglobin.Hemoglobin_storetime,
    ROUND(FIRST_Platelet_Count.VALUENUM::numeric,2) AS FIRST_Platelet_Count,
    FIRST_Platelet_Count.VALUEUOM AS FIRST_Platelet_Count_UOM,
    FIRST_Platelet_Count.Platelet_Count_charttime,
    FIRST_Platelet_Count.Platelet_Count_storetime,
    ROUND(FIRST_RDW.VALUENUM::numeric,2) AS FIRST_RDW,
    FIRST_RDW.VALUEUOM AS FIRST_RDW_UOM,
    FIRST_RDW.RDW_charttime,
    FIRST_RDW.RDW_storetime,
    ROUND(FIRST_Red_Blood_Cells.VALUENUM::numeric,2) AS FIRST_Red_Blood_Cells,
    FIRST_Red_Blood_Cells.VALUEUOM AS FIRST_Red_Blood_Cells_UOM,
    FIRST_Red_Blood_Cells.Red_Blood_Cells_charttime,
    FIRST_Red_Blood_Cells.Red_Blood_Cells_storetime,
    ROUND(FIRST_White_Blood_Cells.VALUENUM::numeric,2) AS
FIRST_White_Blood_Cells,
    FIRST_White_Blood_Cells.VALUEUOM AS FIRST_White_Blood_Cells_UOM,
    FIRST_White_Blood_Cells.White_Blood_Cells_charttime,
    FIRST_White_Blood_Cells.White_Blood_Cells_storetime,
    ROUND(FIRST_Absolute_Neutrophil_Count.VALUENUM::numeric,2) AS
FIRST_Absolute_Neutrophil_Count,
    FIRST_Absolute_Neutrophil_Count.VALUEUOM AS
FIRST_Absolute_Neutrophil_Count_UOM,
    FIRST_Absolute_Neutrophil_Count.Absolute_Neutrophil_Count_charttime,
    FIRST_Absolute_Neutrophil_Count.Absolute_Neutrophil_Count_storetime,
    ROUND(FIRST_Hemoglobin_A1c.VALUENUM::numeric,2) AS FIRST_Hemoglobin_A1c,
```

```
    FIRST_Hemoglobin_A1c.VALUEUOM AS FIRST_Hemoglobin_A1c_UOM,
    FIRST_Hemoglobin_A1c.Hemoglobin_A1c_charttime,
    FIRST_Hemoglobin_A1c.Hemoglobin_A1c_storetime,
    ROUND(FIRST_Albumin.VALUENUM::numeric,2) AS FIRST_Albumin,
    FIRST_Albumin.VALUEUOM AS FIRST_Albumin_UOM,
    FIRST_Albumin.Albumin_charttime,
    FIRST_Albumin.Albumin_storetime,
    ROUND(FIRST_Anion_Gap.VALUENUM::numeric,2) AS FIRST_Anion_Gap,
    FIRST_Anion_Gap.VALUEUOM AS FIRST_Anion_Gap_UOM,
    FIRST_Anion_Gap.Anion_Gap_charttime,
    FIRST_Anion_Gap.Anion_Gap_storetime,
    ROUND(FIRST_Calcium_Total.VALUENUM::numeric,2) AS FIRST_Calcium_Total,
    FIRST_Calcium_Total.VALUEUOM AS FIRST_Calcium_Total_UOM,
    FIRST_Calcium_Total.Calcium_Total_charttime,
    FIRST_Calcium_Total.Calcium_Total_storetime,
    ROUND(FIRST_Chloride.VALUENUM::numeric,2) AS FIRST_Chloride,
    FIRST_Chloride.VALUEUOM AS FIRST_Chloride_UOM,
    FIRST_Chloride.Chloride_charttime,
    FIRST_Chloride.Chloride_storetime,
    ROUND(FIRST_Globulin.VALUENUM::numeric,2) AS FIRST_Globulin,
    FIRST_Globulin.VALUEUOM AS FIRST_Globulin_UOM,
    FIRST_Globulin.Globulin_charttime,
    FIRST_Globulin.Globulin_storetime,
    ROUND(FIRST_Glucose.VALUENUM::numeric,2) AS FIRST_Glucose,
    FIRST_Glucose.VALUEUOM AS FIRST_Glucose_UOM,
    FIRST_Glucose.Glucose_charttime,
    FIRST_Glucose.Glucose_storetime,
    ROUND(FIRST_Potassium.VALUENUM::numeric,2) AS FIRST_Potassium,
    FIRST_Potassium.VALUEUOM AS FIRST_Potassium_UOM,
    FIRST_Potassium.Potassium_charttime,
    FIRST_Potassium.Potassium_storetime,
    ROUND(FIRST_Protein_Total.VALUENUM::numeric,2) AS FIRST_Protein_Total,
    FIRST_Protein_Total.VALUEUOM AS FIRST_Protein_Total_UOM,
    FIRST_Protein_Total.Protein_Total_charttime,
    FIRST_Protein_Total.Protein_Total_storetime,
    ROUND(FIRST_Sodium.VALUENUM::numeric,2) AS FIRST_Sodium,
    FIRST_Sodium.VALUEUOM AS FIRST_Sodium_UOM,
    FIRST_Sodium.Sodium_charttime,
    FIRST_Sodium.Sodium_storetime,
    ROUND(FIRST_Calculated_Total_CO2.VALUENUM::numeric,2) AS
FIRST_Calculated_Total_CO2,
    FIRST_Calculated_Total_CO2.VALUEUOM AS FIRST_Calculated_Total_CO2_UOM,
    FIRST_Calculated_Total_CO2.Calculated_Total_CO2_charttime,
    FIRST_Calculated_Total_CO2.Calculated_Total_CO2_storetime,
```

```
    ROUND(FIRST_Free_Calcium.VALUENUM::numeric,2) AS FIRST_Free_Calcium,
    FIRST_Free_Calcium.VALUEUOM AS FIRST_Free_Calcium_UOM,
    FIRST_Free_Calcium.Free_Calcium_charttime,
    FIRST_Free_Calcium.Free_Calcium_storetime,
    ROUND(FIRST_Lactate.VALUENUM::numeric,2) AS FIRST_Lactate,
    FIRST_Lactate.VALUEUOM AS FIRST_Lactate_UOM,
    FIRST_Lactate.Lactate_charttime,
    FIRST_Lactate.Lactate_storetime,
    ROUND(FIRST_pCO2.VALUENUM::numeric,2) AS FIRST_pCO2,
    FIRST_pCO2.VALUEUOM AS FIRST_pCO2_UOM,
    FIRST_pCO2.pCO2_charttime,
    FIRST_pCO2.pCO2_storetime,
    ROUND(FIRST_pH.VALUENUM::numeric,2) AS FIRST_pH,
    FIRST_pH.VALUEUOM AS FIRST_pH_UOM,
    FIRST_pH.pH_charttime,
    FIRST_pH.pH_storetime,
    ROUND(FIRST_pO2.VALUENUM::numeric,2) AS FIRST_pO2,
    FIRST_pO2.VALUEUOM AS FIRST_pO2_UOM,
    FIRST_pO2.pO2_charttime,
    FIRST_pO2.pO2_storetime,
    ROUND(FIRST_DDimer.VALUENUM::numeric,2) AS FIRST_DDimer,
    FIRST_DDimer.VALUEUOM AS FIRST_DDimer_UOM,
    FIRST_DDimer.DDimer_charttime,
    FIRST_DDimer.DDimer_storetime,
    ROUND(FIRST_Fibrinogen_Functional.VALUENUM::numeric,2) AS
FIRST_Fibrinogen_Functional,
    FIRST_Fibrinogen_Functional.VALUEUOM AS FIRST_Fibrinogen_Functional_UOM,
    FIRST_Fibrinogen_Functional.Fibrinogen_Functional_charttime,
    FIRST_Fibrinogen_Functional.Fibrinogen_Functional_storetime,
    ROUND(FIRST_INRPT.VALUENUM::numeric,2) AS FIRST_INRPT,
    FIRST_INRPT.VALUEUOM AS FIRST_INRPT_UOM,
    FIRST_INRPT.INRPT_charttime,
    FIRST_INRPT.INRPT_storetime,
    ROUND(FIRST_PT.VALUENUM::numeric,2) AS FIRST_PT,
    FIRST_PT.VALUEUOM AS FIRST_PT_UOM,
    FIRST_PT.PT_charttime,
    FIRST_PT.PT_storetime,
    ROUND(FIRST_PTT.VALUENUM::numeric,2) AS FIRST_PTT,
    FIRST_PTT.VALUEUOM AS FIRST_PTT_UOM,
    FIRST_PTT.PTT_charttime,
    FIRST_PTT.PTT_storetime,
    ROUND(FIRST_Thrombin.VALUENUM::numeric,2) AS FIRST_Thrombin,
    FIRST_Thrombin.VALUEUOM AS FIRST_Thrombin_UOM,
    FIRST_Thrombin.Thrombin_charttime,
```

```
    FIRST_Thrombin.Thrombin_storetime,
    ROUND(FIRST_Cholesterol_HDL.VALUENUM::numeric,2) AS FIRST_Cholesterol_HDL,
    FIRST_Cholesterol_HDL.VALUEUOM AS FIRST_Cholesterol_HDL_UOM,
    FIRST_Cholesterol_HDL.Cholesterol_HDL_charttime,
    FIRST_Cholesterol_HDL.Cholesterol_HDL_storetime,
    ROUND(FIRST_Cholesterol_LDL_Calculated.VALUENUM::numeric,2) AS
FIRST_Cholesterol_LDL_Calculated,
    FIRST_Cholesterol_LDL_Calculated.VALUEUOM AS
FIRST_Cholesterol_LDL_Calculated_UOM,
    FIRST_Cholesterol_LDL_Calculated.Cholesterol_LDL_Calculated_charttime,
    FIRST_Cholesterol_LDL_Calculated.Cholesterol_LDL_Calculated_storetime,
    ROUND(FIRST_Cholesterol_Total.VALUENUM::numeric,2) AS FIRST_Cholesterol_Total,
    FIRST_Cholesterol_Total.VALUEUOM AS FIRST_Cholesterol_Total_UOM,
    FIRST_Cholesterol_Total.Cholesterol_Total_charttime,
    FIRST_Cholesterol_Total.Cholesterol_Total_storetime,
    ROUND(FIRST_Triglycerides.VALUENUM::numeric,2) AS FIRST_Triglycerides,
    FIRST_Triglycerides.VALUEUOM AS FIRST_Triglycerides_UOM,
    FIRST_Triglycerides.Triglycerides_charttime,
    FIRST_Triglycerides.Triglycerides_storetime,
    ROUND(FIRST_Alanine_Aminotransferase_ALT.VALUENUM::numeric,2) AS
FIRST_Alanine_Aminotransferase_ALT,
    FIRST_Alanine_Aminotransferase_ALT.VALUEUOM AS
FIRST_Alanine_Aminotransferase_ALT_UOM,
    FIRST_Alanine_Aminotransferase_ALT.Alanine_Aminotransferase_ALT_charttime,
    FIRST_Alanine_Aminotransferase_ALT.Alanine_Aminotransferase_ALT_storetime,
    ROUND(FIRST_Asparate_Aminotransferase_AST.VALUENUM::numeric,2) AS
FIRST_Asparate_Aminotransferase_AST,
    FIRST_Asparate_Aminotransferase_AST.VALUEUOM AS
FIRST_Asparate_Aminotransferase_AST_UOM,
    FIRST_Asparate_Aminotransferase_AST.Asparate_Aminotransferase_AST_charttime,
    FIRST_Asparate_Aminotransferase_AST.Asparate_Aminotransferase_AST_storetime,
    ROUND(FIRST_Bilirubin_Direct.VALUENUM::numeric,2) AS FIRST_Bilirubin_Direct,
    FIRST_Bilirubin_Direct.VALUEUOM AS FIRST_Bilirubin_Direct_UOM,
    FIRST_Bilirubin_Direct.Bilirubin_Direct_charttime,
    FIRST_Bilirubin_Direct.Bilirubin_Direct_storetime,
    ROUND(FIRST_Bilirubin_Indirect.VALUENUM::numeric,2) AS FIRST_Bilirubin_Indirect,
    FIRST_Bilirubin_Indirect.VALUEUOM AS FIRST_Bilirubin_Indirect_UOM,
    FIRST_Bilirubin_Indirect.Bilirubin_Indirect_charttime,
    FIRST_Bilirubin_Indirect.Bilirubin_Indirect_storetime,
    ROUND(FIRST_Bilirubin_Total.VALUENUM::numeric,2) AS FIRST_Bilirubin_Total,
    FIRST_Bilirubin_Total.VALUEUOM AS FIRST_Bilirubin_Total_UOM,
    FIRST_Bilirubin_Total.Bilirubin_Total_charttime,
    FIRST_Bilirubin_Total.Bilirubin_Total_storetime,
    ROUND(FIRST_Creatinine.VALUENUM::numeric,2) AS FIRST_Creatinine,
```

```
    FIRST_Creatinine.VALUEUOM AS FIRST_Creatinine_UOM,
    FIRST_Creatinine.Creatinine_charttime,
    FIRST_Creatinine.Creatinine_storetime,
    ROUND(FIRST_Urea_Nitrogen.VALUENUM::numeric,2) AS FIRST_Urea_Nitrogen,
    FIRST_Urea_Nitrogen.VALUEUOM AS FIRST_Urea_Nitrogen_UOM,
    FIRST_Urea_Nitrogen.Urea_Nitrogen_charttime,
    FIRST_Urea_Nitrogen.Urea_Nitrogen_storetime,
    ROUND(FIRST_Uric_Acid.VALUENUM::numeric,2) AS FIRST_Uric_Acid,
    FIRST_Uric_Acid.VALUEUOM AS FIRST_Uric_Acid_UOM,
    FIRST_Uric_Acid.Uric_Acid_charttime,
    FIRST_Uric_Acid.Uric_Acid_storetime,
    ROUND(FIRST_Creatine_Kinase_CK.VALUENUM::numeric,2) AS
FIRST_Creatine_Kinase_CK,
    FIRST_Creatine_Kinase_CK.VALUEUOM AS FIRST_Creatine_Kinase_CK_UOM,
    FIRST_Creatine_Kinase_CK.Creatine_Kinase_CK_charttime,
    FIRST_Creatine_Kinase_CK.Creatine_Kinase_CK_storetime,
    ROUND(FIRST_Creatine_Kinase_MB_Isoenzyme.VALUENUM::numeric,2) AS
FIRST_Creatine_Kinase_MB_Isoenzyme,
    FIRST_Creatine_Kinase_MB_Isoenzyme.VALUEUOM AS
FIRST_Creatine_Kinase_MB_Isoenzyme_UOM,
    FIRST_Creatine_Kinase_MB_Isoenzyme.Creatine_Kinase_MB_Isoenzyme_charttime,
    FIRST_Creatine_Kinase_MB_Isoenzyme.Creatine_Kinase_MB_Isoenzyme_storetime,
    ROUND(FIRST_Lactate_Dehydrogenase_LD.VALUENUM::numeric,2) AS
FIRST_Lactate_Dehydrogenase_LD,
    FIRST_Lactate_Dehydrogenase_LD.VALUEUOM AS
FIRST_Lactate_Dehydrogenase_LD_UOM,
    FIRST_Lactate_Dehydrogenase_LD.Lactate_Dehydrogenase_LD_charttime,
    FIRST_Lactate_Dehydrogenase_LD.Lactate_Dehydrogenase_LD_storetime,
    ROUND(FIRST_NTproBNP.VALUENUM::numeric,2) AS FIRST_NTproBNP,
    FIRST_NTproBNP.VALUEUOM AS FIRST_NTproBNP_UOM,
    FIRST_NTproBNP.NTproBNP_charttime,
    FIRST_NTproBNP.NTproBNP_storetime,
    ROUND(FIRST_Troponin_T.VALUENUM::numeric,2) AS FIRST_Troponin_T,
    FIRST_Troponin_T.VALUEUOM AS FIRST_Troponin_T_UOM,
    FIRST_Troponin_T.Troponin_T_charttime,
    FIRST_Troponin_T.Troponin_T_storetime,
    ROUND(FIRST_Troponin_I.VALUENUM::numeric,2) AS FIRST_Troponin_I,
    FIRST_Troponin_I.VALUEUOM AS FIRST_Troponin_I_UOM,
    FIRST_Troponin_I.Troponin_I_charttime,
    FIRST_Troponin_I.Troponin_I_storetime,
    ROUND(FIRST_Magnesium.VALUENUM::numeric,2) AS FIRST_Magnesium,
    FIRST_Magnesium.VALUEUOM AS FIRST_Magnesium_UOM,
    FIRST_Magnesium.Magnesium_charttime,
    FIRST_Magnesium.Magnesium_storetime,
```

```
    ROUND(FIRST_Phosphate.VALUENUM::numeric,2) AS FIRST_Phosphate,
    FIRST_Phosphate.VALUEUOM AS FIRST_Phosphate_UOM,
    FIRST_Phosphate.Phosphate_charttime,
    FIRST_Phosphate.Phosphate_storetime,
    ROUND(FIRST_Lymphocytes.VALUENUM::numeric,2) AS FIRST_Lymphocytes,
    FIRST_Lymphocytes.VALUEUOM AS FIRST_Lymphocytes_UOM,
    FIRST_Lymphocytes.Lymphocytes_charttime,
    FIRST_Lymphocytes.Lymphocytes_storetime,
    ROUND(FIRST_Monocytes.VALUENUM::numeric,2) AS FIRST_Monocytes,
    FIRST_Monocytes.VALUEUOM AS FIRST_Monocytes_UOM,
    FIRST_Monocytes.Monocytes_charttime,
    FIRST_Monocytes.Monocytes_storetime,
    ROUND(FIRST_Neutrophils.VALUENUM::numeric,2) AS FIRST_Neutrophils,
    FIRST_Neutrophils.VALUEUOM AS FIRST_Neutrophils_UOM,
    FIRST_Neutrophils.Neutrophils_charttime,
    FIRST_Neutrophils.Neutrophils_storetime,
    ROUND(FIRST_Absolute_Monocyte_Count.VALUENUM::numeric,2) AS
FIRST_Absolute_Monocyte_Count,
    FIRST_Absolute_Monocyte_Count.VALUEUOM AS
FIRST_Absolute_Monocyte_Count_UOM,
    FIRST_Absolute_Monocyte_Count.Absolute_Monocyte_Count_charttime,
    FIRST_Absolute_Monocyte_Count.Absolute_Monocyte_Count_storetime
    FROM basic AS SUBJECT
LEFT JOIN FIRST_Absolute_Lymphocyte_Count ON SUBJECT.HADM_ID =
FIRST_Absolute_Lymphocyte_Count.HADM_ID
LEFT JOIN FIRST_Hematocrit ON SUBJECT.HADM_ID = FIRST_Hematocrit.HADM_ID
LEFT JOIN FIRST_Hemoglobin ON SUBJECT.HADM_ID = FIRST_Hemoglobin.HADM_ID
LEFT JOIN FIRST_Platelet_Count ON SUBJECT.HADM_ID =
FIRST_Platelet_Count.HADM_ID
LEFT JOIN FIRST_RDW ON SUBJECT.HADM_ID = FIRST_RDW.HADM_ID
LEFT JOIN FIRST_Red_Blood_Cells ON SUBJECT.HADM_ID =
FIRST_Red_Blood_Cells.HADM_ID
LEFT JOIN FIRST_White_Blood_Cells ON SUBJECT.HADM_ID =
FIRST_White_Blood_Cells.HADM_ID
LEFT JOIN FIRST_Absolute_Neutrophil_Count ON SUBJECT.HADM_ID =
FIRST_Absolute_Neutrophil_Count.HADM_ID
LEFT JOIN FIRST_Hemoglobin_A1c ON SUBJECT.HADM_ID =
FIRST_Hemoglobin_A1c.HADM_ID
LEFT JOIN FIRST_Albumin ON SUBJECT.HADM_ID = FIRST_Albumin.HADM_ID
LEFT JOIN FIRST_Anion_Gap ON SUBJECT.HADM_ID = FIRST_Anion_Gap.HADM_ID
LEFT JOIN FIRST_Calcium_Total ON SUBJECT.HADM_ID =
FIRST_Calcium_Total.HADM_ID
LEFT JOIN FIRST_Chloride ON SUBJECT.HADM_ID = FIRST_Chloride.HADM_ID
LEFT JOIN FIRST_Globulin ON SUBJECT.HADM_ID = FIRST_Globulin.HADM_ID
```

```
LEFT JOIN FIRST_Glucose ON SUBJECT.HADM_ID = FIRST_Glucose.HADM_ID
LEFT JOIN FIRST_Potassium ON SUBJECT.HADM_ID = FIRST_Potassium.HADM_ID
LEFT JOIN FIRST_Protein_Total ON SUBJECT.HADM_ID = FIRST_Protein_Total.HADM_ID
LEFT JOIN FIRST_Sodium ON SUBJECT.HADM_ID = FIRST_Sodium.HADM_ID
LEFT JOIN FIRST_Calculated_Total_CO2 ON SUBJECT.HADM_ID =
FIRST_Calculated_Total_CO2.HADM_ID
LEFT JOIN FIRST_Free_Calcium ON SUBJECT.HADM_ID =
FIRST_Free_Calcium.HADM_ID
LEFT JOIN FIRST_Lactate ON SUBJECT.HADM_ID = FIRST_Lactate.HADM_ID
LEFT JOIN FIRST_pCO2 ON SUBJECT.HADM_ID = FIRST_pCO2.HADM_ID
LEFT JOIN FIRST_pH ON SUBJECT.HADM_ID = FIRST_pH.HADM_ID
LEFT JOIN FIRST_pO2 ON SUBJECT.HADM_ID = FIRST_pO2.HADM_ID
LEFT JOIN FIRST_DDimer ON SUBJECT.HADM_ID = FIRST_DDimer.HADM_ID
LEFT JOIN FIRST_Fibrinogen_Functional ON SUBJECT.HADM_ID =
FIRST_Fibrinogen_Functional.HADM_ID
LEFT JOIN FIRST_INRPT ON SUBJECT.HADM_ID = FIRST_INRPT.HADM_ID
LEFT JOIN FIRST_PT ON SUBJECT.HADM_ID = FIRST_PT.HADM_ID
LEFT JOIN FIRST_PTT ON SUBJECT.HADM_ID = FIRST_PTT.HADM_ID
LEFT JOIN FIRST_Thrombin ON SUBJECT.HADM_ID = FIRST_Thrombin.HADM_ID
LEFT JOIN FIRST_Cholesterol_HDL ON SUBJECT.HADM_ID =
FIRST_Cholesterol_HDL.HADM_ID
LEFT JOIN FIRST_Cholesterol_LDL_Calculated ON SUBJECT.HADM_ID =
FIRST_Cholesterol_LDL_Calculated.HADM_ID
LEFT JOIN FIRST_Cholesterol_Total ON SUBJECT.HADM_ID =
FIRST_Cholesterol_Total.HADM_ID
LEFT JOIN FIRST_Triglycerides ON SUBJECT.HADM_ID = FIRST_Triglycerides.HADM_ID
LEFT JOIN FIRST_Alanine_Aminotransferase_ALT ON SUBJECT.HADM_ID =
FIRST_Alanine_Aminotransferase_ALT.HADM_ID
LEFT JOIN FIRST_Asparate_Aminotransferase_AST ON SUBJECT.HADM_ID =
FIRST_Asparate_Aminotransferase_AST.HADM_ID
LEFT JOIN FIRST_Bilirubin_Direct ON SUBJECT.HADM_ID =
FIRST_Bilirubin_Direct.HADM_ID
LEFT JOIN FIRST_Bilirubin_Indirect ON SUBJECT.HADM_ID =
FIRST_Bilirubin_Indirect.HADM_ID
LEFT JOIN FIRST_Bilirubin_Total ON SUBJECT.HADM_ID =
FIRST_Bilirubin_Total.HADM_ID
LEFT JOIN FIRST_Creatinine ON SUBJECT.HADM_ID = FIRST_Creatinine.HADM_ID
LEFT JOIN FIRST_Urea_Nitrogen ON SUBJECT.HADM_ID =
FIRST_Urea_Nitrogen.HADM_ID
LEFT JOIN FIRST_Uric_Acid ON SUBJECT.HADM_ID = FIRST_Uric_Acid.HADM_ID
LEFT JOIN FIRST_Creatine_Kinase_CK ON SUBJECT.HADM_ID =
FIRST_Creatine_Kinase_CK.HADM_ID
LEFT JOIN FIRST_Creatine_Kinase_MB_Isoenzyme ON SUBJECT.HADM_ID =
FIRST_Creatine_Kinase_MB_Isoenzyme.HADM_ID
```

```
LEFT JOIN FIRST_Lactate_Dehydrogenase_LD ON SUBJECT.HADM_ID =
FIRST_Lactate_Dehydrogenase_LD.HADM_ID
LEFT JOIN FIRST_NTproBNP ON SUBJECT.HADM_ID = FIRST_NTproBNP.HADM_ID
LEFT JOIN FIRST_Troponin_T ON SUBJECT.HADM_ID = FIRST_Troponin_T.HADM_ID
LEFT JOIN FIRST_Troponin_I ON SUBJECT.HADM_ID = FIRST_Troponin_I.HADM_ID
LEFT JOIN FIRST_Magnesium ON SUBJECT.HADM_ID = FIRST_Magnesium.HADM_ID
LEFT JOIN FIRST_Phosphate ON SUBJECT.HADM_ID = FIRST_Phosphate.HADM_ID
LEFT JOIN FIRST_Lymphocytes ON SUBJECT.HADM_ID = FIRST_Lymphocytes.HADM_ID
LEFT JOIN FIRST_Monocytes ON SUBJECT.HADM_ID = FIRST_Monocytes.HADM_ID
LEFT JOIN FIRST_Neutrophils ON SUBJECT.HADM_ID = FIRST_Neutrophils.HADM_ID
LEFT JOIN FIRST_Absolute_Monocyte_Count ON SUBJECT.HADM_ID =
FIRST_Absolute_Monocyte_Count.HADM_ID
ORDER BY SUBJECT.SUBJECT_ID;
WITH
basic AS (
    WITH
    --Diagnosis included
    -- Cardiogenic shock
    includedDiagnose1 AS (
        SELECT DISTINCT hadm_id
        FROM mimiciv_hosp.diagnoses_icd
        WHERE icd_code IN ('78551')
    ),
    -- Cardiogenic shock
    includedDiagnose2 AS (
        SELECT DISTINCT hadm_id
        FROM mimiciv_hosp.diagnoses_icd
        WHERE icd_code IN ('R570')
    )
    SELECT
        DISTINCT ON (icu.subject_id)
        icu.subject_id,
        icu.stay_id,
        icu.hadm_id,
        icu.los_icu
    FROM mimiciv_derived.icustay_detail AS icu
    JOIN mimiciv_derived.age AS age ON age.hadm_id = icu.hadm_id
    WHERE
        TRUE
        AND age.age BETWEEN 18 AND 120
        AND icu.los_icu > 24.0/24.0
        AND icu.first_icu_stay = true
        AND icu.first_hosp_stay = true
        AND (icu.survival_day >= 1 OR icu.survival_day IS NULL)
```

```
        --Diagnosis included
        AND (
            -- Cardiogenic shock
            icu.hadm_id IN (SELECT hadm_id FROM includedDiagnose1)
            -- Cardiogenic shock
            OR icu.hadm_id IN (SELECT hadm_id FROM includedDiagnose2)
        )
)
--SELECT * FROM basic;

 -- 是不是做过机械通气，做机械通气小时数，首次做机械通气时间
, VENTILATION AS
    (SELECT SUBJECT.STAY_ID,
            ROUND(SUM(MIMICIV_DERIVED.DATETIME_DIFF(VENTILATION.ENDTIM
E,VENTILATION.STARTTIME,'HOUR')),2) AS VENTILATION_HOUR,
            MIN(VENTILATION.STARTTIME) AS VENTILATION_FIRST_TIME
        FROM basic AS SUBJECT,
            MIMICIV_DERIVED.VENTILATION AS VENTILATION,
        MIMICIV_DERIVED.ICUSTAY_DETAIL AS ICU
        WHERE SUBJECT.STAY_ID = VENTILATION.STAY_ID
                AND SUBJECT.STAY_ID = ICU.STAY_ID
         AND VENTILATION.STARTTIME >= ICU.ICU_INTIME

        GROUP BY SUBJECT.STAY_ID)
SELECT SUBJECT.SUBJECT_ID,
    SUBJECT.STAY_ID,
    SUBJECT.HADM_ID,
    VENTILATION.VENTILATION_HOUR,
    CASE WHEN VENTILATION.VENTILATION_HOUR IS NOT NULL THEN 1 ELSE
NULL END AS VENTILATION,
    VENTILATION.VENTILATION_FIRST_TIME
FROM basic AS SUBJECT
LEFT JOIN VENTILATION ON SUBJECT.STAY_ID = VENTILATION.STAY_ID
ORDER BY SUBJECT.SUBJECT_ID;
basic AS (
    WITH
    --Diagnosis included
    -- Cardiogenic shock
    includedDiagnose1 AS (
        SELECT DISTINCT hadm_id
        FROM mimiciv_hosp.diagnoses_icd
        WHERE icd_code IN ('78551')
    ),
    -- Cardiogenic shock
```

```
    includedDiagnose2 AS (
        SELECT DISTINCT hadm_id
        FROM mimiciv_hosp.diagnoses_icd
        WHERE icd_code IN ('R570')
    )
    SELECT
        DISTINCT ON (icu.subject_id)
        icu.subject_id,
        icu.stay_id,
        icu.hadm_id,
        icu.los_icu
    FROM mimiciv_derived.icustay_detail AS icu
    JOIN mimiciv_derived.age AS age ON age.hadm_id = icu.hadm_id
    WHERE
        TRUE
        AND age.age BETWEEN 18 AND 120
        AND icu.los_icu > 24.0/24.0
        AND icu.first_icu_stay = true
        AND icu.first_hosp_stay = true
        AND (icu.survival_day >= 1 OR icu.survival_day IS NULL)
        --Diagnosis included
        AND (
            -- Cardiogenic shock
            icu.hadm_id IN (SELECT hadm_id FROM includedDiagnose1)
            -- Cardiogenic shock
            OR icu.hadm_id IN (SELECT hadm_id FROM includedDiagnose2)
        )
)
--SELECT * FROM basic;

-- 是不是做过 CRRT，做 CRRT 天数，首次做 CRRT 时间
, CRRT AS
    (SELECT SUBJECT.STAY_ID,
            COUNT(DISTINCT DATE(CHARTTIME)) AS CRRT_DAY,
            MIN(CRRT.CHARTTIME) AS CRRT_FIRST_TIME
        FROM basic AS SUBJECT,
            MIMICIV_DERIVED.CRRT AS CRRT,
        MIMICIV_DERIVED.ICUSTAY_DETAIL AS ICU
        WHERE SUBJECT.STAY_ID = CRRT.STAY_ID
                 AND SUBJECT.STAY_ID = ICU.STAY_ID
         AND CRRT.CHARTTIME >= ICU.ICU_INTIME
        GROUP BY SUBJECT.STAY_ID)
SELECT SUBJECT.SUBJECT_ID,
    SUBJECT.STAY_ID,
```

```
    SUBJECT.HADM_ID,
    CASE WHEN CRRT.CRRT_DAY IS NOT NULL THEN 1 ELSE NULL END AS CRRT,
    CRRT.CRRT_DAY,
    CRRT.CRRT_FIRST_TIME
FROM basic AS SUBJECT
LEFT JOIN CRRT ON SUBJECT.STAY_ID = CRRT.STAY_ID
ORDER BY SUBJECT.SUBJECT_ID;
SELECT
        DISTINCT ON (icu.subject_id)
        icu.subject_id,
        icu.stay_id,
        icu.hadm_id,
        icu.los_icu
    FROM mimiciv_derived.icustay_detail AS icu
    JOIN mimiciv_derived.age AS age ON age.hadm_id = icu.hadm_id
    WHERE
        TRUE
        AND age.age BETWEEN 18 AND 120
        AND icu.los_icu > 24.0/24.0
        AND icu.first_icu_stay = true
        AND icu.first_hosp_stay = true
        AND (icu.survival_day >= 1 OR icu.survival_day IS NULL)
        --Diagnosis included
        AND (
            -- Cardiogenic shock
            icu.hadm_id IN (SELECT hadm_id FROM includedDiagnose1)
            -- Cardiogenic shock
            OR icu.hadm_id IN (SELECT hadm_id FROM includedDiagnose2)
        )
)
--SELECT * FROM basic;


,
-- SedativeAnalgesic
SA AS
(SELECT DISTINCT(prescriptions.hadm_id),
        1 AS flag,
        MAX(prescriptions.starttime) AS SA_prescriptions_starttime,
        MAX(prescriptions.stoptime) AS SA_prescriptions_stoptime
FROM mimiciv_hosp.prescriptions AS prescriptions,
     basic AS subject
WHERE prescriptions.hadm_id = subject.hadm_id
```

```
    AND prescriptions.gsn IN
('041385','041384','048287','015883','015882','015881','059102','015880','022358','016796','06588
8','066228','003780','070134','051279','003779','074967','070306','070132','044671','070877')
GROUP BY prescriptions.hadm_id
),
SA_val AS
(SELECT DISTINCT(prescriptions.hadm_id),
SUM(CAST(prescriptions.dose_val_rx AS numeric)) AS dose_val_rx,
        MAX(prescriptions.dose_unit_rx) AS dose_unit_rx
  FROM mimiciv_hosp.prescriptions AS prescriptions, basic AS subject
  WHERE prescriptions.hadm_id = subject.hadm_id
        AND prescriptions.gsn IN
('041385','041384','048287','015883','015882','015881','059102','015880','022358','016796','06588
8','066228','003780','070134','051279','003779','074967','070306','070132','044671','070877')
        AND prescriptions.dose_val_rx ~ '^[0-9]+(\.[0-9]+)?$'
  GROUP BY prescriptions.hadm_id
  ),
-- Vasopressor
VP AS
(SELECT DISTINCT(prescriptions.hadm_id),
         1 AS flag,
         MAX(prescriptions.starttime) AS VP_prescriptions_starttime,
         MAX(prescriptions.stoptime) AS VP_prescriptions_stoptime
FROM mimiciv_hosp.prescriptions AS prescriptions,
     basic AS subject
WHERE prescriptions.hadm_id = subject.hadm_id
    AND prescriptions.gsn IN
('004977','004985','004975','064575','062006','004939','066419','066452','004937','004931','06533
6','028633','003388','003389','003390','003385','052187','004934','003387','052188','003386','0080
22','008062','005068','063864','063863','066206','005066','074949','007764','008061','048541','060
981','073081','006612','000141','064535','021502','064538')
GROUP BY prescriptions.hadm_id
),
VP_val AS
(SELECT DISTINCT(prescriptions.hadm_id),
SUM(CAST(prescriptions.dose_val_rx AS numeric)) AS dose_val_rx,
        MAX(prescriptions.dose_unit_rx) AS dose_unit_rx
  FROM mimiciv_hosp.prescriptions AS prescriptions, basic AS subject
  WHERE prescriptions.hadm_id = subject.hadm_id
        AND prescriptions.gsn IN
('004977','004985','004975','064575','062006','004939','066419','066452','004937','004931','06533
6','028633','003388','003389','003390','003385','052187','004934','003387','052188','003386','0080
22','008062','005068','063864','063863','066206','005066','074949','007764','008061','048541','060
981','073081','006612','000141','064535','021502','064538')
```

```
          AND prescriptions.dose_val_rx ~ '^[0-9]+(\.[0-9]+)?$'
   GROUP BY prescriptions.hadm_id
   ),
--Glucocorticoids
GC AS
(SELECT DISTINCT(prescriptions.hadm_id),
           1 AS flag,
           MAX(prescriptions.starttime) AS GC_prescriptions_starttime,
           MAX(prescriptions.stoptime) AS GC_prescriptions_stoptime
FROM mimiciv_hosp.prescriptions AS prescriptions,
      basic AS subject
WHERE prescriptions.hadm_id = subject.hadm_id
     AND prescriptions.gsn IN
('066110','006705','051558','006704','007544','023906','006696','006858','007545','007543','006724','006725','006753','007894','007892','006786','006784','006788','006776','006778','006789','006721','067556','047282','006745','060958','062053','006780','013701','006762','006758','006812','066112','026721','006749','006738','006742','006754','006748','006750')
GROUP BY prescriptions.hadm_id
),
GC_val AS
(SELECT DISTINCT(prescriptions.hadm_id),
SUM(CAST(prescriptions.dose_val_rx AS numeric)) AS dose_val_rx,
          MAX(prescriptions.dose_unit_rx) AS dose_unit_rx
   FROM mimiciv_hosp.prescriptions AS prescriptions, basic AS subject
   WHERE prescriptions.hadm_id = subject.hadm_id
          AND prescriptions.gsn IN
('066110','006705','051558','006704','007544','023906','006696','006858','007545','007543','006724','006725','006753','007894','007892','006786','006784','006788','006776','006778','006789','006721','067556','047282','006745','060958','062053','006780','013701','006762','006758','006812','066112','026721','006749','006738','006742','006754','006748','006750')
          AND prescriptions.dose_val_rx ~ '^[0-9]+(\.[0-9]+)?$'
   GROUP BY prescriptions.hadm_id
   )

   SELECT
        DISTINCT(subject.subject_id),
        subject.stay_id,
        subject.hadm_id,
        SA.flag AS SA,SA_val.dose_val_rx AS SAtotalval, SA_val.dose_unit_rx AS SAunit,
        SA.SA_prescriptions_starttime,
        SA.SA_prescriptions_stoptime,
        VP.flag AS VP,VP_val.dose_val_rx AS VPtotalval, VP_val.dose_unit_rx AS VPunit,
        VP.VP_prescriptions_starttime,
        VP.VP_prescriptions_stoptime,
```

```
        GC.flag AS GC,GC_val.dose_val_rx AS GCtotalval, GC_val.dose_unit_rx AS GCunit,
        GC.GC_prescriptions_starttime,
        GC.GC_prescriptions_stoptime
FROM basic AS subject
--SedativeAnalgesic
LEFT JOIN SA ON subject.hadm_id = SA.hadm_id
LEFT JOIN SA_val ON subject.hadm_id = SA_val.hadm_id
--（升压药） Vasopressor
LEFT JOIN VP ON subject.hadm_id = VP.hadm_id
LEFT JOIN VP_val ON subject.hadm_id = VP_val.hadm_id
-- Glucocorticoids
LEFT JOIN GC ON subject.hadm_id = GC.hadm_id
LEFT JOIN GC_val ON subject.hadm_id = GC_val.hadm_id
ORDER BY subject.subject_id;

,
YuHou_Final AS (
    SELECT
        subject.subject_id,
        MAX(icu.admittime) AS admit_time,
        MAX(icu.dischtime) AS disch_time,
        MAX(icu.icu_intime) AS icu_intime,
        MAX(icu.icu_outtime) AS icu_outtime,
        MAX(admission.deathtime) AS dead_time,
        MAX(icu.dod) AS icu_dod,
                CASE WHEN MAX(admission.deathtime) IS NOT NULL OR MAX(icu.dod)IS
NOT NULL THEN 1 ELSE NULL END AS is_dead,

                CASE
                    WHEN MAX(admission.deathtime) IS NOT NULL
                    AND MAX(admission.deathtime) NOT BETWEEN MAX(icu.icu_intime)
AND MAX(icu.icu_outtime) THEN 1
                    ELSE 0
                END AS is_hosp_dead,
                CASE
                   WHEN MAX(admission.deathtime) BETWEEN MAX(icu.icu_intime) AND
MAX(icu.icu_outtime) THEN 1
                   ELSE 0
                END AS is_icu_dead
    FROM
        basic AS subject
    LEFT JOIN mimiciv_derived.icustay_detail AS icu ON subject.subject_id = icu.subject_id
    LEFT JOIN mimiciv_hosp.admissions AS admission ON admission.subject_id =
icu.subject_id
```

```
    GROUP BY subject.subject_id
),

YuHou AS (
    SELECT
        subject.subject_id,
        icu.stay_id,
        icu.hadm_id,
-- 患者总 ICU 停留时间
subject.los_icu,
        icu.admittime AS admit_time,
        icu.dischtime AS disch_time,
        icu.icu_intime AS icu_intime,
        icu.icu_outtime AS icu_outtime,
        YuHou_Final.dead_time,
        YuHou_Final.icu_dod,
            YuHou_Final.is_dead,
        CASE
            WHEN adm.deathtime IS NOT NULL THEN 1
            ELSE 0
        END AS death_in_current_adm,

        YuHou_Final.is_hosp_dead,
        YuHou_Final.is_icu_dead,
        ROUND(EXTRACT(EPOCH FROM (icu.dischtime - icu.admittime)) / 86400, 2) AS
hosp_day,
        ROUND(EXTRACT(EPOCH FROM (icu.icu_outtime - icu.icu_intime)) / 86400, 2) AS
icu_day,
        ROUND(EXTRACT(EPOCH FROM (YuHou_Final.dead_time - icu.admittime)) / 86400,
2) AS hosp_survival_time,
        ROUND(EXTRACT(EPOCH FROM (YuHou_Final.dead_time - icu.icu_intime)) / 86400,
2) AS icu_survival_time,
        CASE
            WHEN YuHou_Final.dead_time IS NOT NULL AND
                (YuHou_Final.dead_time - icu.admittime) <= INTERVAL '28 days'
            THEN 1
            ELSE 0
        END AS death_within_hosp_28days,
        CASE
            WHEN YuHou_Final.dead_time IS NOT NULL AND
                (YuHou_Final.dead_time - icu.icu_intime) <= INTERVAL ' 28 days'
            THEN 1
            ELSE 0
        END AS death_within_icu_28days
```

```sql
    FROM
        basic AS subject
    LEFT JOIN mimiciv_derived.icustay_detail AS icu
        ON subject.subject_id = icu.subject_id
    LEFT JOIN mimiciv_hosp.admissions AS adm
        ON icu.hadm_id = adm.hadm_id
    LEFT JOIN YuHou_Final
        ON YuHou_Final.subject_id = subject.subject_id
    GROUP BY
        subject.subject_id,
        icu.stay_id,
        icu.hadm_id,
        icu.admittime,
        icu.dischtime,
        icu.icu_intime,
        icu.icu_outtime,
        YuHou_Final.dead_time,
        YuHou_Final.icu_dod,
        YuHou_Final.is_dead,
        YuHou_Final.is_hosp_dead,
        YuHou_Final.is_icu_dead,
        adm.deathtime,subject.los_icu
)

SELECT * FROM YuHou ORDER BY subject_id,admit_time
```
