# Supplementary material for: The red blood cell distribution width-to-albumin ratio and 28-day mortality in patients with severe cardiogenic shock: a retrospective cohort study based on MIMIC-IV with single-center external validation
Source: Front Med (Lausanne). 2026 Sep 30;13:1857535. doi: 10.3389/fmed.2026.1857535 (PMC13661497; doi:10.3389/fmed.2026.1857535)
Supplement: Supplementary file 2 [file Datasheet2.pdf]

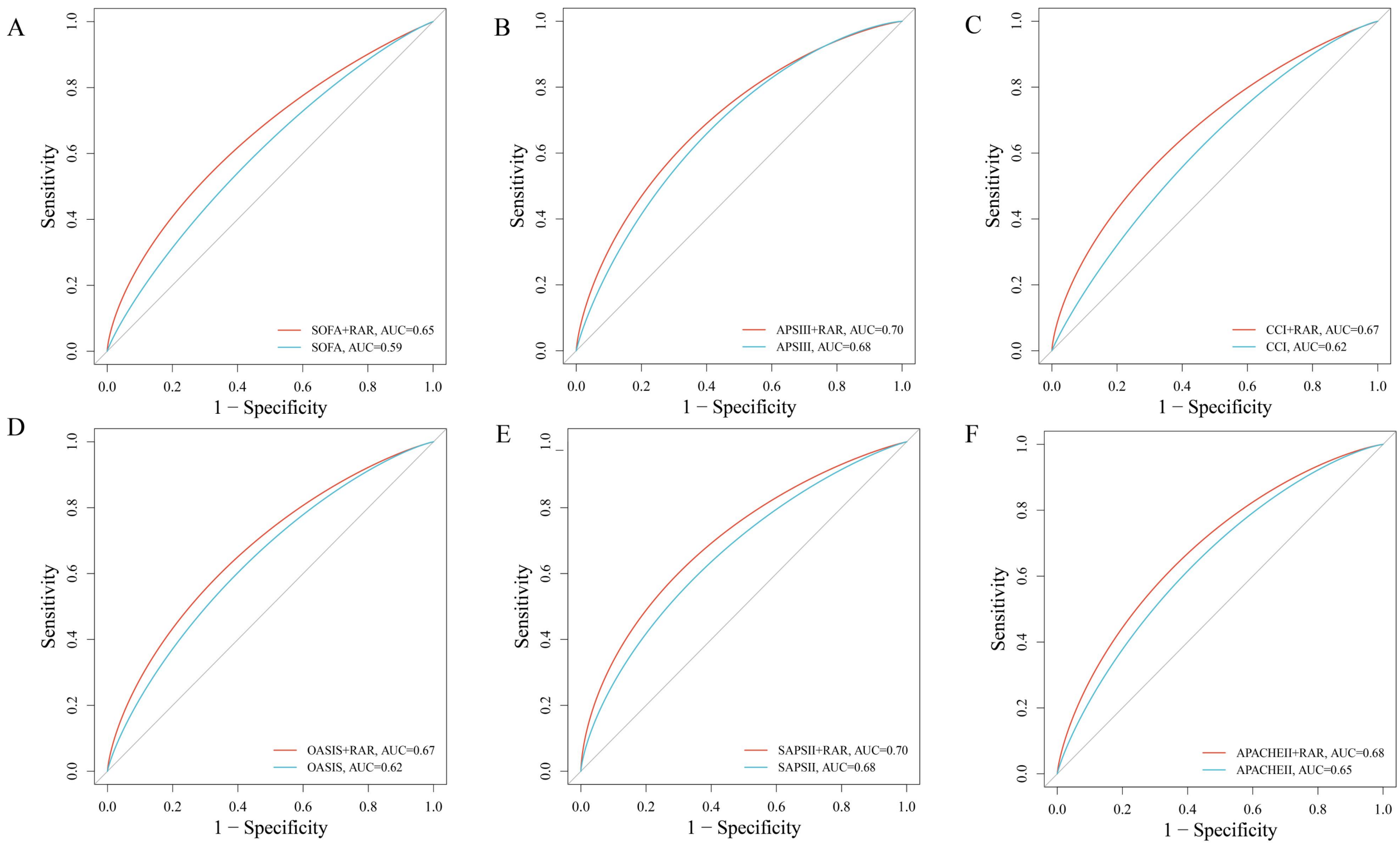

Figure S1: Incremental Predictive Value of RAR Added to Traditional Severity Scores for 28-Day ICU Mortality in Cardiogenic Shock.

Note: This figure illustrates the improvement in discriminative ability (assessed by the area under the receiver operating characteristic curve, AUC) when the red cell distribution width-to-albumin ratio (RAR) is added to each of six traditional critical illness severity scores for predicting 28-day ICU mortality in patients with cardiogenic shock. The AUC values for each score alone (light bars) and for the score combined with RAR (dark bars) are presented in separate panels. (A) SOFA: SOFA alone, AUC = 0.59; SOFA + RAR, AUC = 0.65; (B) APS III: APS III alone, AUC = 0.68; APS III + RAR, AUC = 0.70; (C) CCI: CCI alone, AUC = 0.62; CCI + RAR, AUC = 0.67; (D) OASIS: OASIS alone, AUC = 0.62; OASIS + RAR, AUC = 0.67; (E) SAPS II: SAPS II alone, AUC = 0.68; SAPS II + RAR, AUC = 0.70; (F) APACHE II: APACHE II alone, AUC = 0.65; APACHE II + RAR, AUC = 0.68.

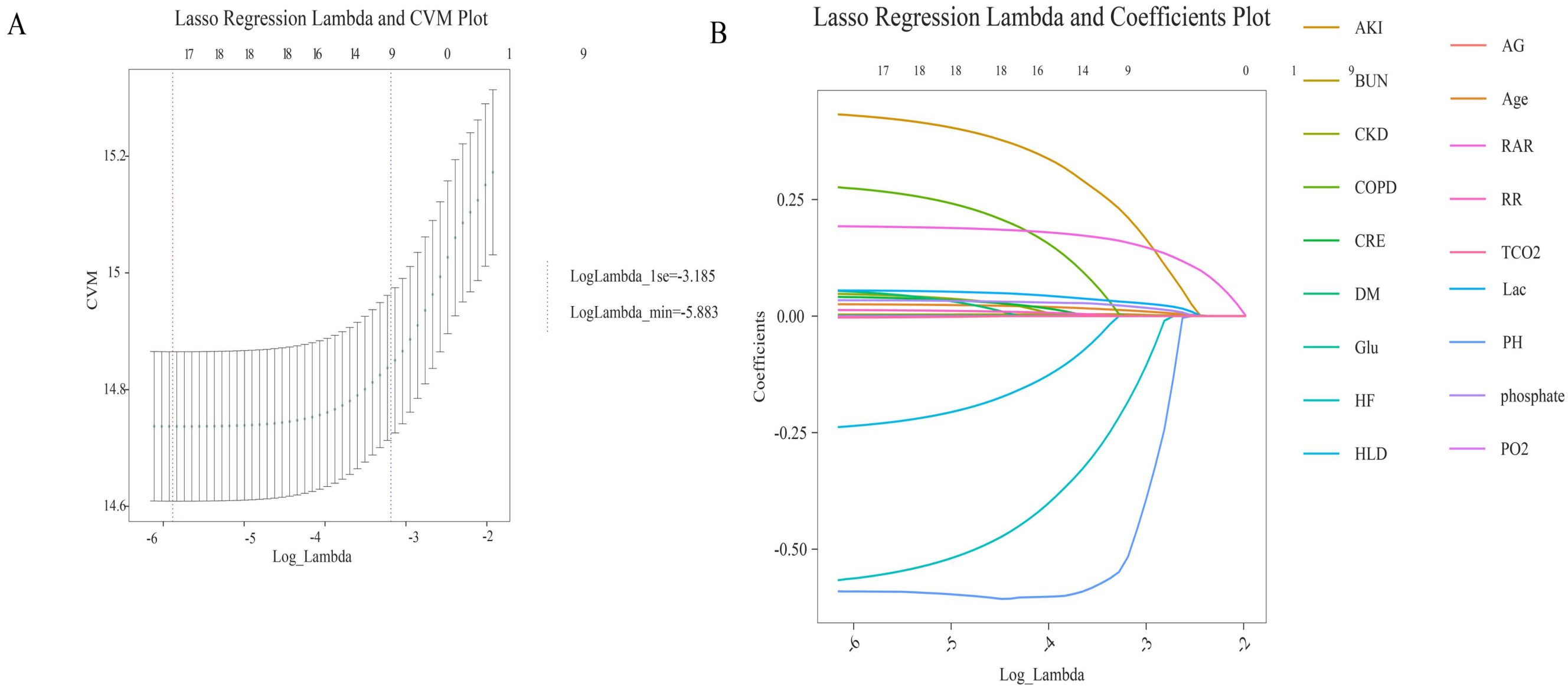

Figure S2: Feature Selection via LASSO Regression.

Note: (A) Cross-validated mean squared error (MSE) plotted against log(λ). Left vertical dashed line: λ at minimum MSE (λ.min). Right vertical dashed line: largest λ within one standard error of the minimum MSE (λ.1se), chosen for a more parsimonious model. (B) LASSO coefficient profiles of candidate variables. Each curve shows the coefficient path of a variable as the penalty term log(λ) increases. The vertical dashed line at the selected λ.1se identifies the final set of features with non-zero coefficients.

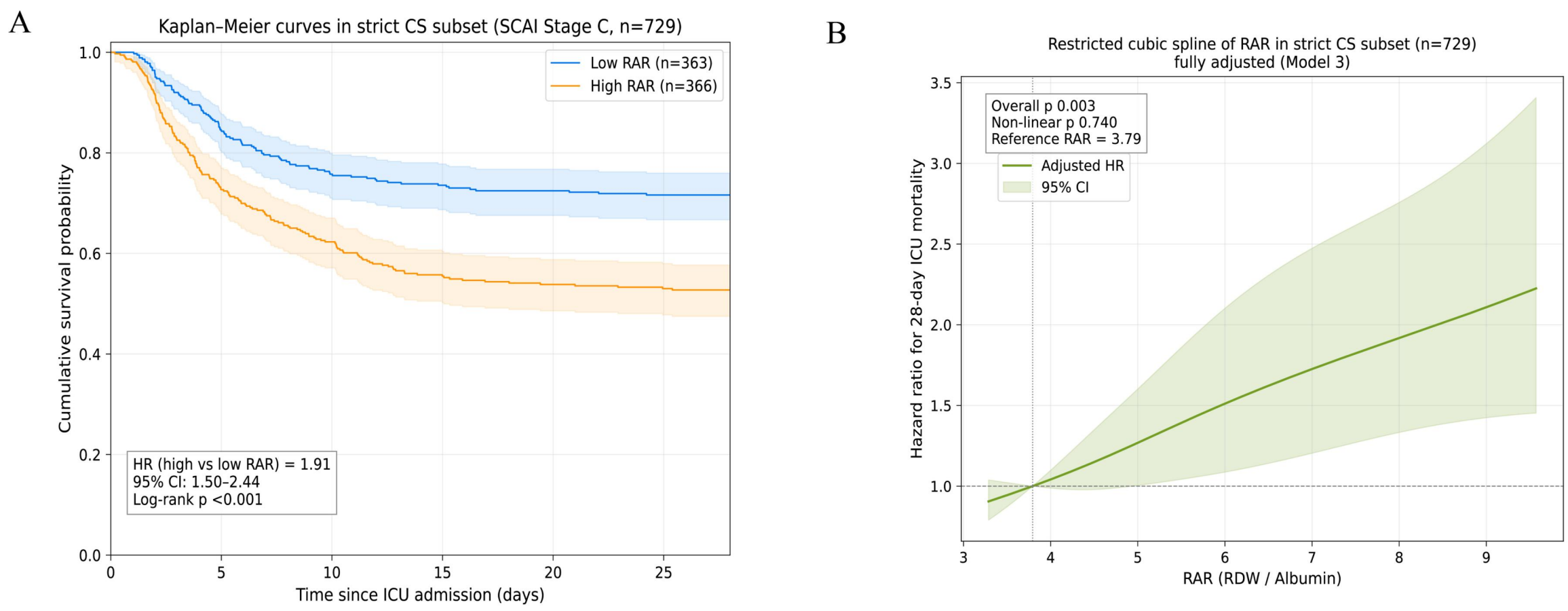

Figure S3: sensitivity analysis in strict CS subset

Note: (A) Kaplan–Meier curves for 28-day ICU mortality in the strict CS subset, high vs low RAR (median split). HR 1.91 (95% CI 1.50–2.44), log-rank $p < 0.001$. (B) Restricted cubic spline of RAR versus 28-day ICU mortality in the strict CS subset, fully adjusted (Model 3). Overall p = 0.003; non-linear p = 0.74.

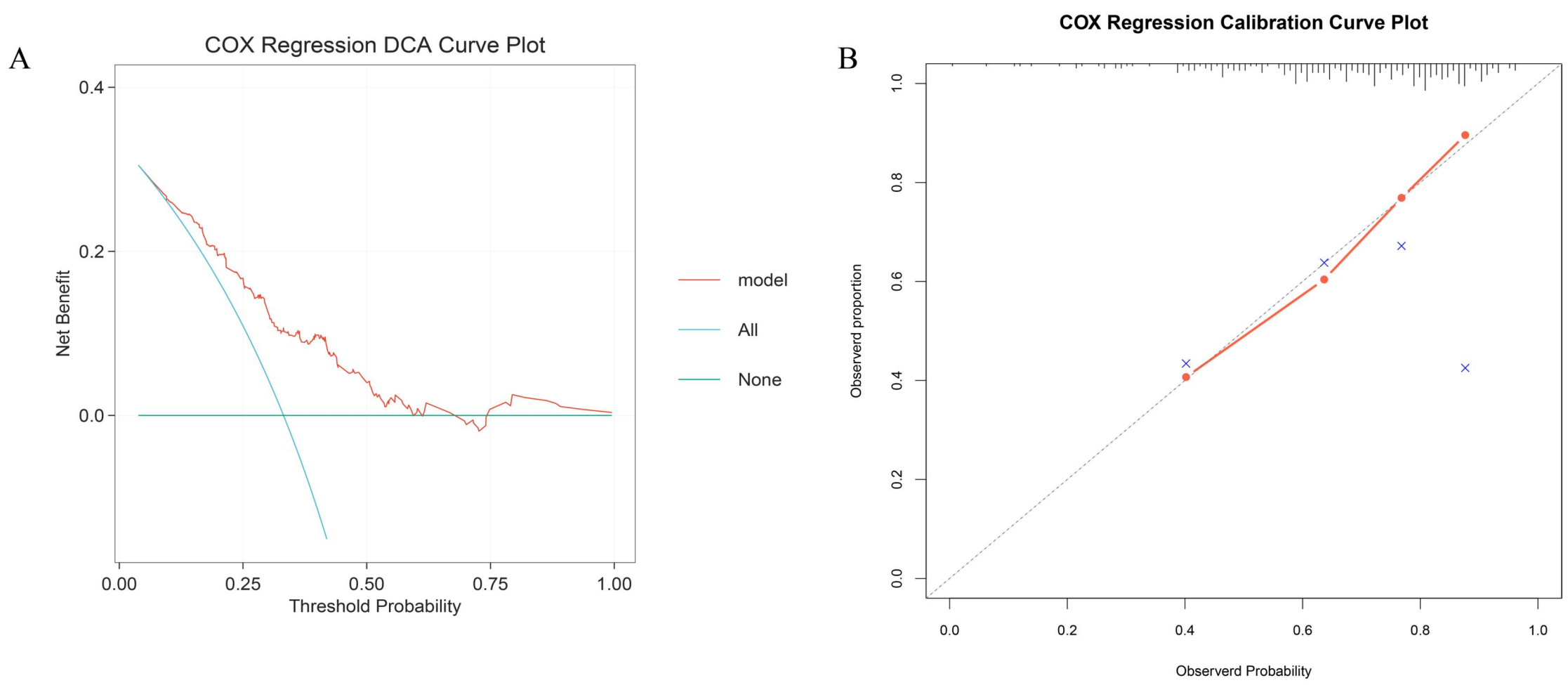

Figure S4: Clinical utility and calibration of the prognostic model in the internal test set.

Note: (A) Decision curve analysis (DCA) showing net benefit across threshold probabilities. The model curve (blue) remains above the "All" and "None" strategies across a wide range of thresholds (approximately 0.2–0.8), indicating clinical net benefit. (B) Calibration curve showing agreement between predicted and observed probabilities in the internal test set. The curve aligns closely with the ideal diagonal, demonstrating good calibration.

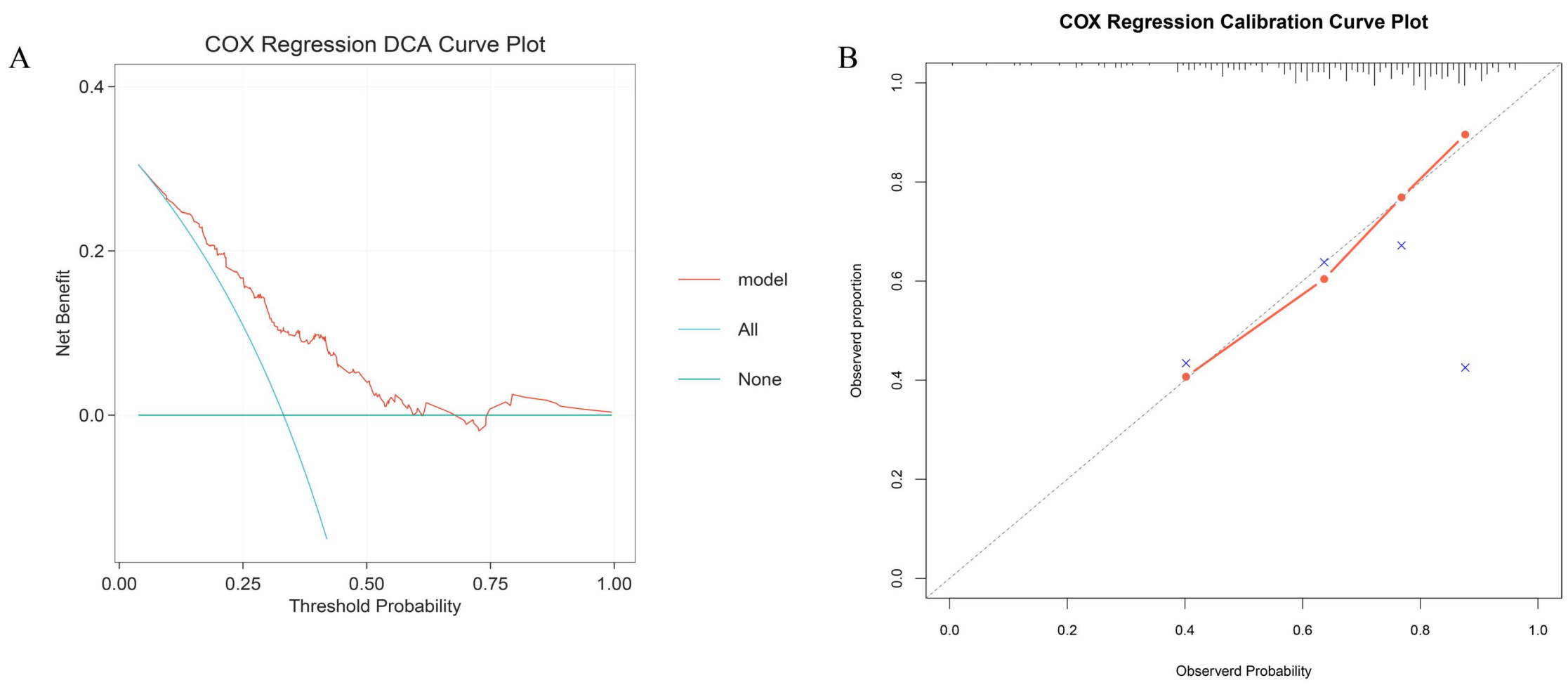

Figure S5: Clinical utility and calibration of the prognostic model in the external validation cohort.

Note: (A) Decision curve analysis (DCA) showing net benefit across threshold probabilities. (B) Calibration curve showing agreement between predicted and observed probabilities. The model demonstrates good clinical utility and calibration in the independent external cohort

TableS1: Baseline characteristics and clinical outcomes of internal cohort patients stratified by survival status

**Variable**	**ALL**	**Survivor**	**No-survivor**	**P-value**
	N=1314	*N=870*	*N=444*	
RAR (RDW/Albumin), unitless	5.23 (1.61)	4.96 (1.30)	5.77 (1.98)	<0.001
RAR quartile:				<0.001
Q1	328 (25.0%)	255 (29.3%)	73 (16.4%)	
Q2	328 (25.0%)	241 (27.7%)	87 (19.6%)	
Q3	328 (25.0%)	200 (23.0%)	128 (28.8%)	
Q4	330 (25.1%)	174 (20.0%)	156 (35.1%)	
Age, years	68.8 (14.6)	67.2 (14.8)	72.1 (13.8)	<0.001
Gender:				0.676
F	512 (39.0%)	335 (38.5%)	177 (39.9%)	
M	802 (61.0%)	535 (61.5%)	267 (60.1%)	
Race:				0.161
Other races	526 (40.0%)	336 (38.6%)	190 (42.8%)	
White	788 (60.0%)	534 (61.4%)	254 (57.2%)	
BMI, kg/m²	29.2 (7.44)	29.0 (7.15)	29.5 (7.97)	0.264
Hypertension:				0.093

Variable	ALL *N=1314*	Survivor *N=870*	No-survivor *N=444*	P-value
No	1001 (76.2%)	650 (74.7%)	351 (79.1%)	
Yes	313 (23.8%)	220 (25.3%)	93 (20.9%)	
AKI:				<0.001
No	402 (30.6%)	312 (35.9%)	90 (20.3%)	
Yes	912 (69.4%)	558 (64.1%)	354 (79.7%)	
CKD:				<0.001
No	887 (67.5%)	617 (70.9%)	270 (60.8%)	
Yes	427 (32.5%)	253 (29.1%)	174 (39.2%)	
Diabetes:				0.018
No	829 (63.1%)	569 (65.4%)	260 (58.6%)	
Yes	485 (36.9%)	301 (34.6%)	184 (41.4%)	
Hyperlipidemia:				0.033
No	720 (54.8%)	458 (52.6%)	262 (59.0%)	
Yes	594 (45.2%)	412 (47.4%)	182 (41.0%)	
HF:				0.001
No	290 (22.1%)	168 (19.3%)	122 (27.5%)	

Variable	ALL<br>*N=1314*	Survivor<br>*N=870*	No-survivor<br>*N=444*	P-value
Yes	1024 (77.9%)	702 (80.7%)	322 (72.5%)	
MI:				0.574
No	855 (65.1%)	561 (64.5%)	294 (66.2%)	
Yes	459 (34.9%)	309 (35.5%)	150 (33.8%)	
IHD:				0.536
No	416 (31.7%)	270 (31.0%)	146 (32.9%)	
Yes	898 (68.3%)	600 (69.0%)	298 (67.1%)	
COPD:				0.005
No	1074 (81.7%)	730 (83.9%)	344 (77.5%)	
Yes	240 (18.3%)	140 (16.1%)	100 (22.5%)	
HR, beats/min	92.5 (22.7)	91.9 (22.4)	93.8 (23.2)	0.151
NBPS, mmHg	110 (22.2)	111 (21.8)	110 (23.0)	0.533
NBPD, mmHg	66.7 (18.6)	67.4 (18.4)	65.4 (18.9)	0.075
RR, insp/min	20.7 (6.69)	20.3 (6.77)	21.5 (6.46)	0.002
SpO_2, %	95.9 (5.46)	96.1 (5.22)	95.6 (5.88)	0.125
Temperature, °F	97.6 (5.68)	97.6 (6.21)	97.6 (4.46)	0.947

Variable	ALL	Survivor	No-survivor	P-value
	N=1314	*N=870*	*N=444*	
SOFA, Score	7.70 (3.62)	7.27 (3.51)	8.53 (3.70)	<0.001
APS III, Score	59.3 (21.9)	54.6 (20.1)	68.3 (22.4)	<0.001
SAPS II, Score	46.3 (14.5)	43.2 (13.3)	52.4 (14.7)	<0.001
OASIS, Score	36.2 (8.94)	34.8 (8.69)	38.8 (8.86)	<0.001
CCI, Score	6.28 (2.82)	5.86 (2.73)	7.11 (2.81)	<0.001
APACHE II, Score	22.9 (7.50)	21.5 (7.26)	25.5 (7.28)	<0.001
HCT, %	34.4 (7.28)	34.9 (7.34)	33.4 (7.08)	<0.001
Hb, g/dL	11.1 (2.46)	11.3 (2.49)	10.7 (2.35)	<0.001
PLT, K/μL	207 (102)	207 (96.8)	208 (113)	0.873
RDW, %	15.4 (2.54)	15.1 (2.27)	16.1 (2.88)	<0.001
RBC, M/μL	3.75 (0.84)	3.82 (0.85)	3.62 (0.80)	<0.001
WBC, K/μL	14.5 (8.04)	14.1 (6.88)	15.2 (9.89)	0.053
ALB, g/dL	3.08 (0.59)	3.15 (0.55)	2.95 (0.63)	<0.001
AG, mEq/L	16.7 (5.16)	16.2 (4.97)	17.7 (5.37)	<0.001
TCa, mg/dL	8.34 (0.91)	8.34 (0.89)	8.33 (0.96)	0.806
Cl, mEq/L	102 (7.35)	102 (7.42)	101 (7.23)	0.499

Variable	ALL<br>*N=1314*	Survivor<br>*N=870*	No-survivor<br>*N=444*	P-value
GLU, mg/dL	177 (89.4)	172 (81.6)	187 (102)	0.011
K, mEq/L	4.46 (0.86)	4.42 (0.84)	4.52 (0.90)	0.064
Na, mEq/L	137 (5.91)	137 (5.80)	137 (6.11)	0.083
Mg, mg/dL	2.17 (0.57)	2.18 (0.56)	2.14 (0.61)	0.262
TCO2, mEq/L	22.8 (5.51)	23.2 (5.40)	22.1 (5.66)	0.001
Lac, mmol/L	3.11 (2.58)	2.85 (2.27)	3.63 (3.04)	<0.001
PCO_2, mmHg	42.4 (11.9)	42.0 (11.0)	43.1 (13.5)	0.139
pH, pH units	7.33 (0.11)	7.34 (0.10)	7.31 (0.12)	<0.001
PO_2, mmHg	131 (116)	136 (120)	121 (107)	0.026
INR, Ratio	1.87 (1.39)	1.83 (1.38)	1.95 (1.39)	0.116
PT, seconds	20.2 (13.8)	19.7 (13.5)	21.2 (14.5)	0.066
APTT, seconds	53.5 (37.7)	53.3 (37.5)	53.9 (38.1)	0.755
ALT, IU/L	335 (922)	340 (929)	325 (912)	0.777
AST, IU/L	535 (1606)	550 (1543)	505 (1724)	0.638
TB, mg/dL	1.26 (1.71)	1.18 (1.28)	1.41 (2.33)	0.053
CRE, mg/dL	1.97 (1.64)	1.84 (1.60)	2.22 (1.69)	<0.001

**Variable**	**ALL**	**Survivor**	**No-survivor**	**P-value**
	N=1314	*N=870*	*N=444*	
BUN, mg/dL	37.9 (25.4)	34.8 (23.5)	43.9 (27.7)	<0.001
CK-MB, ng/mL	53.2 (94.6)	55.4 (97.0)	48.8 (89.7)	0.224
LDH, IU/L	903 (1551)	861 (1533)	986 (1585)	0.173
cTnI, ng/mL	1.96 (3.95)	1.97 (3.76)	1.92 (4.30)	0.839
Phosphate, mg/dL	4.52 (1.74)	4.34 (1.63)	4.88 (1.89)	<0.001
Ventilation duration, hours	113 (128)	121 (140)	99.2 (96.4)	0.001
Ventilation:				0.376
No	116 (8.83%)	72 (8.28%)	44 (9.91%)	
Yes	1198 (91.2%)	798 (91.7%)	400 (90.1%)	
CRRT:				<0.001
No	1038 (79.0%)	737 (84.7%)	301 (67.8%)	
Yes	276 (21.0%)	133 (15.3%)	143 (32.2%)	
CRRT duration, days	1.55 (5.02)	1.34 (5.45)	1.95 (4.03)	0.021
Sympathomimetic/inotrope:				<0.001
No	237 (18.0%)	182 (20.9%)	55 (12.4%)	
Yes	1077 (82.0%)	688 (79.1%)	389 (87.6%)	

Variable	ALL	Survivor	No-survivor	P-value
	N=1314	*N=870*	*N=444*	
Vasopressor:				0.025
No	22 (1.67%)	20 (2.30%)	2 (0.45%)	
Yes	1292 (98.3%)	850 (97.7%)	442 (99.5%)	
Glucocorticoid:				<0.001
No	963 (73.3%)	667 (76.7%)	296 (66.7%)	
Yes	351 (26.7%)	203 (23.3%)	148 (33.3%)	
Hospital length of stay, days	17.1 (16.6)	20.5 (18.6)	10.4 (8.68)	<0.001
ICU length of stay, days	8.57 (8.54)	9.36 (9.63)	7.01 (5.53)	<0.001
28-day survival time, days	20.9 (10.4)	28.0 (0.00)	7.01 (5.53)	<0.001

Note: Data are presented as mean ± standard deviation or n (%). Abbreviations: Red Cell Distribution Width-to-Albumin Ratio (RAR), Body Mass Index (BMI), Acute Kidney Injury (AKI), Chronic Kidney Disease (CKD), Diabetes Mellitus (DM), Hyperlipidemia (HLD), Heart Failure (HF), Myocardial Infarction (MI), Ischemic Heart Disease (IHD), Chronic Obstructive Pulmonary Disease (COPD), Heart Rate (HR), Non-Invasive Blood Pressure—Systolic (NBPS), Non-Invasive Blood Pressure—Diastolic (NBPD), Respiratory Rate (RR), Peripheral Oxygen Saturation (SpO_2), Temperature (Fahrenheit), Sequential Organ Failure Assessment (SOFA), Acute Physiology Score III (APS III), Simplified Acute Physiology Score II (SAPS II), Oxford Acute Severity of Illness Score (OASIS), Charlson Comorbidity Index (CCI), Acute Physiology and Chronic Health Evaluation II (APACHE II), Hematocrit (HCT), Hemoglobin (Hb), Platelet Count (PLT), Red Cell Distribution Width (RDW), Red Blood Cell Count (RBC), White Blood Cell Count (WBC), Albumin (ALB), Anion Gap (AG), Total Calcium (TCa), Chloride (Cl), Glucose (Glu), Potassium (K), Sodium (Na), Magnesium (Mg), Total Carbon Dioxide (TCO_2), Lactate (Lac), Partial Pressure of Carbon Dioxide (PCO_2), Potential of

Hydrogen (pH), Partial Pressure of Oxygen (PO_2), International Normalized Ratio (INR), Prothrombin Time (PT), Activated Partial Thromboplastin Time (APTT), Alanine Aminotransferase (ALT), Aspartate Aminotransferase (AST), Total Bilirubin (TB), Creatinine (CRE), Blood Urea Nitrogen (BUN), Creatine Kinase-MB (CK- MB), Lactate Dehydrogenase (LDH), Cardiac Troponin I (cTnI), Phosphate, Ventilation Hours, Ventilation, Continuous Renal Replacement Therapy (CRRT), CRRT Days, Vasopressor Administration (sa), Total Vasopressor Dose (satotalval), Vasopressor Type VP, Total VP Dose (vptotalval), Glucocorticoid Use (GC), Total Glucocorticoid Dose (gctotalval), Hospital Length of Stay (hosp_day), ICU Length of Stay (icu_day).

TableS2: Baseline characteristics and outcomes of internal cohort patients stratified by in- hospital survival status

**Variable**	**ALL**	**Survivor**	**No-survivor**	**P-value**

	N=1314	*N=824*	*N=490*	
RAR (RDW/Albumin), unitless	5.23 (1.61)	4.91 (1.26)	5.78 (1.95)	<0.001
RAR quartile:				<0.001
Q1	328 (25.0%)	250 (30.3%)	78 (15.9%)	
Q2	328 (25.0%)	234 (28.4%)	94 (19.2%)	
Q3	328 (25.0%)	189 (22.9%)	139 (28.4%)	
Q4	330 (25.1%)	151 (18.3%)	179 (36.5%)	
Age, years	68.8 (14.6)	67.2 (14.7)	71.7 (14.1)	<0.001
Gender:				0.515
F	512 (39.0%)	315 (38.2%)	197 (40.2%)	
M	802 (61.0%)	509 (61.8%)	293 (59.8%)	
Race:				0.120
Other races	526 (40.0%)	316 (38.3%)	210 (42.9%)	
White	788 (60.0%)	508 (61.7%)	280 (57.1%)	
BMI, kg/m²	29.2 (7.44)	29.0 (7.11)	29.5 (7.96)	0.333
Hypertension:				0.021
No	1001 (76.2%)	610 (74.0%)	391 (79.8%)	
Yes	313 (23.8%)	214 (26.0%)	99 (20.2%)	

**Variable**	**ALL**	**Survivor**	**No-survivor**	**P-value**
	N=1314	*N=824*	*N=490*	
AKI:				<0.001
No	402 (30.6%)	295 (35.8%)	107 (21.8%)	
Yes	912 (69.4%)	529 (64.2%)	383 (78.2%)	
CKD:				<0.001
No	887 (67.5%)	588 (71.4%)	299 (61.0%)	
Yes	427 (32.5%)	236 (28.6%)	191 (39.0%)	
Diabetes:				0.020
No	829 (63.1%)	540 (65.5%)	289 (59.0%)	
Yes	485 (36.9%)	284 (34.5%)	201 (41.0%)	
Hyperlipidemia:				0.085
No	720 (54.8%)	436 (52.9%)	284 (58.0%)	
Yes	594 (45.2%)	388 (47.1%)	206 (42.0%)	
HF:				0.005
No	290 (22.1%)	161 (19.5%)	129 (26.3%)	
Yes	1024 (77.9%)	663 (80.5%)	361 (73.7%)	
MI:				0.202

Variable	ALL	Survivor	No-survivor	P-value
	N=1314	*N=824*	*N=490*	
No	855 (65.1%)	525 (63.7%)	330 (67.3%)	
Yes	459 (34.9%)	299 (36.3%)	160 (32.7%)	
IHD:				0.203
No	416 (31.7%)	250 (30.3%)	166 (33.9%)	
Yes	898 (68.3%)	574 (69.7%)	324 (66.1%)	
COPD:				0.005
No	1074 (81.7%)	693 (84.1%)	381 (77.8%)	
Yes	240 (18.3%)	131 (15.9%)	109 (22.2%)	
HR, beats/min	92.5 (22.7)	91.6 (22.3)	94.1 (23.2)	0.051
NBPS, mmHg	110 (22.2)	111 (21.8)	109 (23.0)	0.309
NBPD, mmHg	66.7 (18.6)	67.3 (18.2)	65.7 (19.3)	0.130
RR, insp/min	20.7 (6.69)	20.3 (6.73)	21.4 (6.57)	0.005
SpO_2, %	95.9 (5.46)	96.1 (5.28)	95.7 (5.74)	0.152
Temperature, °F	97.6 (5.68)	97.6 (6.37)	97.6 (4.27)	0.818
SOFA, Score	7.70 (3.62)	7.24 (3.48)	8.46 (3.73)	<0.001
APS III, Score	59.3 (21.9)	54.4 (20.1)	67.4 (22.3)	<0.001

Variable	ALL	Survivor	No-survivor	P-value
	N=1314	*N=824*	*N=490*	
SAPS II, Score	46.3 (14.5)	43.2 (13.3)	51.5 (14.9)	<0.001
OASIS, Score	36.2 (8.94)	34.8 (8.71)	38.5 (8.87)	<0.001
CCI, Score	6.28 (2.82)	5.85 (2.73)	7.00 (2.83)	<0.001
APACHE II, Score	22.9 (7.50)	21.4 (7.20)	25.3 (7.36)	<0.001
HCT, %	34.4 (7.28)	35.1 (7.29)	33.3 (7.14)	<0.001
Hb, g/dL	11.1 (2.46)	11.4 (2.48)	10.7 (2.37)	<0.001
PLT, K/μL	207 (102)	208 (97.4)	206 (110)	0.812
RDW, %	15.4 (2.54)	15.0 (2.21)	16.1 (2.87)	<0.001
RBC, M/μL	3.75 (0.84)	3.83 (0.84)	3.62 (0.81)	<0.001
WBC, K/μL	14.5 (8.04)	14.1 (6.73)	15.1 (9.83)	0.061
ALB, g/dL	3.08 (0.59)	3.16 (0.55)	2.95 (0.63)	<0.001
AG, mEq/L	16.7 (5.16)	16.2 (4.90)	17.6 (5.45)	<0.001
TCa, mg/dL	8.34 (0.91)	8.34 (0.89)	8.34 (0.94)	0.946
Cl, mEq/L	102 (7.35)	102 (7.43)	101 (7.23)	0.321
GLU, mg/dL	177 (89.4)	173 (82.7)	183 (99.4)	0.065
K, mEq/L	4.46 (0.86)	4.42 (0.84)	4.51 (0.90)	0.069

**Variable**	**ALL**	**Survivor**	**No-survivor**	**P-value**
	N=1314	*N=824*	*N=490*	
Na, mEq/L	137 (5.91)	137 (5.84)	137 (6.03)	0.166
Mg, mg/dL	2.17 (0.57)	2.17 (0.55)	2.15 (0.61)	0.576
TCO2, mEq/L	22.8 (5.51)	23.2 (5.35)	22.2 (5.72)	0.003
Lac, mmol/L	3.11 (2.58)	2.82 (2.22)	3.59 (3.04)	<0.001
PCO_2, mmHg	42.4 (11.9)	42.0 (10.9)	42.9 (13.4)	0.209
pH, pH units	7.33 (0.11)	7.34 (0.10)	7.32 (0.12)	<0.001
PO_2, mmHg	131 (116)	136 (120)	122 (109)	0.023
INR, Ratio	1.87 (1.39)	1.84 (1.41)	1.92 (1.34)	0.294
PT, seconds	20.2 (13.8)	19.8 (13.8)	20.8 (14.0)	0.187
APTT, seconds	53.5 (37.7)	53.4 (37.4)	53.7 (38.2)	0.888
ALT, IU/L	335 (922)	351 (951)	309 (873)	0.417
AST, IU/L	535 (1606)	571 (1581)	475 (1647)	0.303
TB, mg/dL	1.26 (1.71)	1.18 (1.27)	1.39 (2.26)	0.064
CRE, mg/dL	1.97 (1.64)	1.82 (1.47)	2.22 (1.87)	<0.001
BUN, mg/dL	37.9 (25.4)	34.7 (23.0)	43.2 (28.1)	<0.001
CK-MB, ng/mL	53.2 (94.6)	56.3 (98.6)	47.9 (87.4)	0.111

Variable	ALL	Survivor	No-survivor	P-value
	N=1314	*N=824*	*N=490*	
LDH, IU/L	903 (1551)	873 (1559)	954 (1538)	0.363
cTnI, ng/mL	1.96 (3.95)	2.01 (3.84)	1.87 (4.14)	0.526
Phosphate, mg/dL	4.52 (1.74)	4.34 (1.62)	4.83 (1.89)	<0.001
Ventilation duration, hours	113 (128)	121 (136)	100 (112)	0.002
Ventilation:				0.209
No	116 (8.83%)	66 (8.01%)	50 (10.2%)	
Yes	1198 (91.2%)	758 (92.0%)	440 (89.8%)	
CRRT:				<0.001
No	1038 (79.0%)	700 (85.0%)	338 (69.0%)	
Yes	276 (21.0%)	124 (15.0%)	152 (31.0%)	
CRRT duration, days	1.55 (5.02)	1.31 (5.31)	1.96 (4.46)	0.017
Sympathomimetic/inotrope:				0.005
No	237 (18.0%)	168 (20.4%)	69 (14.1%)	
Yes	1077 (82.0%)	656 (79.6%)	421 (85.9%)	
Vasopressor:				0.100
No	22 (1.67%)	18 (2.18%)	4 (0.82%)	

Variable	ALL<br>*N=1314*	Survivor<br>*N=824*	No-survivor<br>*N=490*	P-value
Yes	1292 (98.3%)	806 (97.8%)	486 (99.2%)	
Glucocorticoid:				<0.001
No	963 (73.3%)	637 (77.3%)	326 (66.5%)	
Yes	351 (26.7%)	187 (22.7%)	164 (33.5%)	
Hospital length of stay, days	17.1 (16.6)	20.5 (18.7)	11.5 (10.3)	<0.001
ICU length of stay, days	8.57 (8.54)	9.34 (9.31)	7.26 (6.87)	<0.001
Survival time, days	20.9 (10.4)	28.0 (0.00)	8.98 (8.08)	<0.001

Note: Data are presented as mean ± standard deviation or n (%). Abbreviations: Red Cell Distribution Width-to-Albumin Ratio (RAR), Body Mass Index (BMI), Acute Kidney Injury (AKI), Chronic Kidney Disease (CKD), Diabetes Mellitus (DM), Hyperlipidemia (HLD), Heart Failure (HF), Myocardial Infarction (MI), Ischemic Heart Disease (IHD), Chronic Obstructive Pulmonary Disease (COPD), Heart Rate (HR), Non-Invasive Blood Pressure—Systolic (NBPS), Non-Invasive Blood Pressure—Diastolic (NBPD), Respiratory Rate (RR), Peripheral Oxygen Saturation (SpO_2), Temperature (Fahrenheit), Sequential Organ Failure Assessment (SOFA), Acute Physiology Score III (APS III), Simplified Acute Physiology Score II (SAPS II), Oxford Acute Severity of Illness Score (OASIS), Charlson Comorbidity Index (CCI), Acute Physiology and Chronic Health Evaluation II (APACHE II), Hematocrit (HCT), Hemoglobin (Hb), Platelet Count (PLT), Red Cell Distribution Width (RDW), Red Blood Cell Count (RBC), White Blood Cell Count (WBC), Albumin (ALB), Anion Gap (AG), Total Calcium (TCa), Chloride (Cl), Glucose (Glu), Potassium (K), Sodium (Na), Magnesium (Mg), Total Carbon Dioxide (TCO_2), Lactate (Lac), Partial Pressure of Carbon Dioxide (PCO_2), Potential of Hydrogen (pH), Partial Pressure of Oxygen (PO_2), International Normalized Ratio (INR), Prothrombin Time (PT), Activated Partial Thromboplastin Time (APTT), Alanine Aminotransferase (ALT), Aspartate Aminotransferase (AST), Total Bilirubin (TB), Creatinine (CRE), Blood Urea Nitrogen (BUN), Creatine Kinase-MB (CK- MB), Lactate Dehydrogenase (LDH), Cardiac Troponin I (cTnI), Phosphate, Ventilation Hours, Ventilation, Continuous Renal Replacement Therapy (CRRT),

CRRT Days, Vasopressor Administration (sa), Total Vasopressor Dose (satotalval), Vasopressor Type VP, Total VP Dose (vptotalval), Glucocorticoid Use (GC), Total Glucocorticoid Dose (gctotalval), Hospital Length of Stay (hosp_day), ICU Length of Stay (icu_day).

Table S3: Baseline characteristics of the external validation cohort stratified by survival status

**Variable**	**ALL**<br>*N=276*	**Survivor**<br>*N=187*	**No-survivor**<br>*N=89*	**P-value**
RAR (RDW/Albumin), unitless	5.04 (1.45)	4.77 (1.18)	5.62 (1.78)	<0.001

**Variable**	**ALL**	**Survivor**	**No-survivor**	**P-value**
	N=276	*N=187*	*N=89*	
Age, years	70.2 (13.7)	68.6 (13.9)	73.6 (12.5)	0.004
Gender:				0.791
F	107 (38.8%)	74 (39.6%)	33 (37.1%)	
M	169 (61.2%)	113 (60.4%)	56 (62.9%)	
BMI, kg/m²	29.1 (7.15)	28.9 (6.26)	29.6 (8.75)	0.490
Hypertension:				0.681
No	205 (74.3%)	137 (73.3%)	68 (76.4%)	
Yes	71 (25.7%)	50 (26.7%)	21 (23.6%)	
AKI:				0.018
No	97 (35.1%)	75 (40.1%)	22 (24.7%)	
Yes	179 (64.9%)	112 (59.9%)	67 (75.3%)	
CKD:				0.051
No	179 (64.9%)	129 (69.0%)	50 (56.2%)	
Yes	97 (35.1%)	58 (31.0%)	39 (43.8%)	
Diabetes:				0.281
No	154 (55.8%)	109 (58.3%)	45 (50.6%)	

**Variable**	**ALL**	**Survivor**	**No-survivor**	**P-value**
	N=276	*N=187*	*N=89*	
Yes	122 (44.2%)	78 (41.7%)	44 (49.4%)	
Hyperlipidemia:				0.116
No	147 (53.3%)	93 (49.7%)	54 (60.7%)	
Yes	129 (46.7%)	94 (50.3%)	35 (39.3%)	
HF:				1.000
No	56 (20.3%)	38 (20.3%)	18 (20.2%)	
Yes	220 (79.7%)	149 (79.7%)	71 (79.8%)	
MI:				0.053
No	181 (65.6%)	115 (61.5%)	66 (74.2%)	
Yes	95 (34.4%)	72 (38.5%)	23 (25.8%)	
IHD:				0.443
No	80 (29.0%)	51 (27.3%)	29 (32.6%)	
Yes	196 (71.0%)	136 (72.7%)	60 (67.4%)	
COPD:				0.495
No	225 (81.5%)	155 (82.9%)	70 (78.7%)	
Yes	51 (18.5%)	32 (17.1%)	19 (21.3%)	

Variable	ALL	Survivor	No-survivor	P-value
	N=276	*N=187*	*N=89*	
HR, beats/min	90.7 (21.5)	90.4 (22.0)	91.4 (20.5)	0.711
NBPS, mmHg	111 (21.9)	110 (21.9)	113 (22.0)	0.336
NBPD, mmHg	66.8 (18.3)	67.4 (18.3)	65.5 (18.4)	0.413
RR, insp/min	20.5 (6.31)	19.9 (6.11)	21.9 (6.54)	0.014
SpO_2, %	96.3 (4.84)	96.1 (5.07)	96.8 (4.31)	0.269
Temperature, °F	97.7 (4.05)	97.7 (4.78)	97.5 (1.68)	0.539
SOFA, Score	7.64 (3.64)	7.25 (3.60)	8.47 (3.61)	0.009
APS III, Score	59.4 (21.5)	54.8 (19.8)	69.0 (21.9)	<0.001
SAPS II, Score	46.3 (14.8)	43.6 (14.2)	52.0 (14.5)	<0.001
OASIS, Score	36.0 (8.93)	34.8 (8.37)	38.4 (9.61)	0.003
CCI, Score	6.57 (2.71)	6.16 (2.64)	7.43 (2.65)	<0.001
APACHE II, Score	22.9 (7.38)	21.6 (7.12)	25.6 (7.22)	<0.001
HCT, %	34.5 (7.56)	35.2 (7.72)	33.2 (7.06)	0.032
Hb, g/dL	11.2 (2.53)	11.4 (2.58)	10.7 (2.35)	0.017
PLT, K/μL	213 (94.5)	222 (100.0)	195 (79.6)	0.019
RDW, %	15.4 (2.53)	14.9 (2.22)	16.3 (2.89)	<0.001

Variable	ALL	Survivor	No-survivor	P-value
	N=276	*N=187*	*N=89*	
RBC, M/µL	3.75 (0.82)	3.79 (0.84)	3.66 (0.79)	0.226
WBC, K/µL	14.4 (7.15)	14.3 (6.05)	14.4 (9.09)	0.941
ALB, g/dL	3.17 (0.58)	3.24 (0.56)	3.03 (0.58)	0.005
AG, mEq/L	16.8 (4.93)	16.3 (4.69)	17.9 (5.25)	0.013
TCa, mg/dL	8.44 (0.88)	8.46 (0.86)	8.40 (0.93)	0.586
Cl, mEq/L	101 (7.57)	102 (7.64)	99.9 (7.24)	0.022
Glu, mg/dL	185 (98.9)	183 (88.7)	190 (118)	0.610
K, mEq/L	4.47 (0.84)	4.41 (0.80)	4.62 (0.92)	0.066
Na, mEq/L	137 (5.86)	137 (5.79)	136 (5.95)	0.122
Mg, mg/dL	2.21 (0.60)	2.24 (0.67)	2.13 (0.43)	0.091
TCO2, mEq/L	23.0 (5.47)	23.1 (5.19)	22.9 (6.05)	0.708
Lactate, mmol/L	3.03 (2.26)	2.82 (1.98)	3.47 (2.72)	0.047
PCO_2, mmHg	42.6 (11.8)	41.6 (10.4)	44.8 (14.1)	0.057
pH, pH units	7.33 (0.11)	7.34 (0.10)	7.31 (0.13)	0.020
PO_2, mmHg	135 (120)	129 (116)	148 (127)	0.246
INR, Ratio	1.81 (1.24)	1.74 (1.32)	1.96 (1.04)	0.131

**Variable**	**ALL**	**Survivor**	**No-survivor**	**P-value**
	N=276	*N=187*	*N=89*	
PT, seconds	19.7 (13.5)	19.0 (14.5)	21.2 (11.0)	0.156
APTT, seconds	51.4 (35.6)	50.1 (33.6)	54.1 (39.6)	0.413
ALT, IU/L	255 (685)	230 (498)	309 (969)	0.467
AST, IU/L	407 (954)	390 (844)	441 (1157)	0.713
TB, mg/dL	1.18 (1.56)	1.04 (1.09)	1.48 (2.22)	0.080
CRE, mg/dL	1.95 (1.43)	1.76 (1.36)	2.36 (1.49)	0.002
BUN, mg/dL	37.8 (25.2)	33.4 (21.3)	47.1 (30.0)	<0.001
CK-MB, ng/mL	56.0 (100)	64.1 (106)	39.0 (84.6)	0.036
LDH, IU/L	822 (1213)	777 (1058)	918 (1490)	0.423
cTnI, ng/mL	1.93 (4.46)	1.93 (3.30)	1.91 (6.25)	0.973
Phosphate, mg/dL	4.58 (1.58)	4.34 (1.44)	5.09 (1.75)	0.001
Ventilation duration, hours	126 (131)	133 (142)	112 (103)	0.163
Mechanical ventilation:				0.485
No	19 (6.88%)	11 (5.88%)	8 (8.99%)	
Yes	257 (93.1%)	176 (94.1%)	81 (91.0%)	
Survival time, days	21.2 (10.2)	26.6 (5.23)	9.83 (8.47)	<0.001

Note: Data are presented as mean ± standard deviation or n (%). Abbreviations: Red Cell Distribution Width-to-Albumin Ratio (RAR), Body Mass Index (BMI), Acute Kidney Injury (AKI), Chronic Kidney Disease (CKD), Diabetes Mellitus (DM), Hyperlipidemia (HLD), Heart Failure (HF), Myocardial Infarction (MI), Ischemic Heart Disease (IHD), Chronic Obstructive Pulmonary Disease (COPD), Heart Rate (HR), Non-Invasive Blood Pressure—Systolic (NBPS), Non-Invasive Blood Pressure—Diastolic (NBPD), Respiratory Rate (RR), Peripheral Oxygen Saturation (SpO_2), Temperature (Fahrenheit), Sequential Organ Failure Assessment (SOFA), Acute Physiology Score III (APS III), Simplified Acute Physiology Score II (SAPS II), Oxford Acute Severity of Illness Score (OASIS), Charlson Comorbidity Index (CCI), Acute Physiology and Chronic Health Evaluation II (APACHE II), Hematocrit (HCT), Hemoglobin (Hb), Platelet Count (PLT), Red Cell Distribution Width (RDW), Red Blood Cell Count (RBC), White Blood Cell Count (WBC), Albumin (ALB), Anion Gap (AG), Total Calcium (TCa), Chloride (Cl), Glucose (Glu), Potassium (K), Sodium (Na), Magnesium (Mg), Total Carbon Dioxide (TCO_2), Lactate (Lac), Partial Pressure of Carbon Dioxide (PCO_2), Potential of Hydrogen (pH), Partial Pressure of Oxygen (PO_2), International Normalized Ratio (INR), Prothrombin Time (PT), Activated Partial Thromboplastin Time (APTT), Alanine Aminotransferase (ALT), Aspartate Aminotransferase (AST), Total Bilirubin (TB), Creatinine (CRE), Blood Urea Nitrogen (BUN), Creatine Kinase-MB (CK- MB), Lactate Dehydrogenase (LDH), Cardiac Troponin I (cTnI), Phosphate

Table S4: Coherent linear screening (VIF) in each cohort

ICU-28Dead		Hosp_28Dead		external validation cohort	
**Variable Names**	**VIF**	**Variable Names**	**VIF**	**Variable Names**	**VIF**
VP	1.05	GC	1.09	RR	1.25
GC	1.08	COPD	1.16	CK_MB	1.30
HLD	1.09	RR	1.21	PLT	1.39

ICU-28Dead		Hosp_28Dead		external validation cohort	
**Variable Names**	**VIF**	**Variable Names**	**VIF**	**Variable Names**	**VIF**
COPD	1.15	AKI	1.26	AKI	1.45
RR	1.21	Sa	1.29	Cl	1.46
Ventilation	1.24	DM	1.30	RAR	1.56
PO2	1.25	PO2	1.30	CRRT	1.72
HF	1.30	ventilation_hour	1.31	PH	1.90
Sa	1.31	RAR	1.38	CKD	2.20
AKI	1.31	HF	1.39	Age	2.30
RAR	1.36	hypertension	1.41	phosphate	2.34
Glu	1.42	TCO2	1.79	CCI	2.56
ventilation_hour	1.52	PH	1.86	CRE	2.59
DM	1.56	CKD	1.91	BUN	2.72
TCO2	1.58	Age	1.91	Lac	2.86
Lac	1.74	phosphate	2.16	AG	2.96
CKD	1.77	CRE	2.27	SOFA	3.21
PH	1.77	CRRT_day	2.38	APACHEII	3.58
phosphate	1.81	BUN	2.39	OASIS	3.80
Age	1.97	CRRT	2.40	APSIII	3.83
CRE	2.21	CCI	2.50	SAPSII	5.52
BUN	2.31	Lac	2.71	HCT	15.12
CRRT	2.48	SOFA	2.71	Hb	16.17
CRRT_day	2.58	OASIS	2.80		
CCI	2.64	AG	3.31		
OASIS	2.76	APACHEII	3.55		
SOFA	2.79	APSIII	4.24		

ICU-28Dead		Hosp_28Dead		external validation cohort	
**Variable Names**	**VIF**	**Variable Names**	**VIF**	**Variable Names**	**VIF**
APACHEII	3.50	SAPSII	5.08		
APSIII	4.29	RBC	7.73		
SAPSII	5.08	Hb	20.47		
RBC	7.65	HCT	27.39		
Hb	21.59				
HCT	27.57				

Table S5: Cohort comparability

**Variable**	**MIMIC-IV (n=1314)**	**External (n=276)**	**p**
Age, years	68.8 ± 14.6	70.2 ± 13.7	0.21
SBP, mmHg	110.3 ± 22.2	111.0 ± 21.9	0.70
SBP <90, %	16.4%	14.5%	0.48
Lactate, mmol/L	3.1 ± 2.6	3.0 ± 2.3	0.98
Lactate >2, %	55.6%	56.9%	0.74
SOFA	7.7 ± 3.6	7.6 ± 3.6	0.88
APACHE II	22.9 ± 7.5	22.9 ± 7.4	0.99
Mechanical ventilation, %	91.2%	93.1%	0.35
Vasopressor use, %	98.3%	99.5%	0.96
28-day ICU mortality, %	33.8%	32.2%	0.67

Table S6: Cox in strict CS subset sensitivity analysis

RAR	Model 1 (unadjusted)	Model 2 (demo+comorbid)	Model 3 (fully adjusted)
Continuous RAR	1.212 (1.140–1.289), p<0.001	1.205 (1.127–1.288), p<0.001	1.146 (1.067–1.231), p<0.001
Q2 vs Q1	1.125 (0.767–1.650), p=0.55	1.125 (0.764–1.655), p=0.55	0.883 (0.593–1.316), p=0.54
Q3 vs Q1	1.876 (1.317–2.673), p<0.001	1.755 (1.222–2.518), p=0.002	1.401 (0.957–2.051), p=0.083
Q4 vs Q1	2.176 (1.541–3.072), p<0.001	2.042 (1.437–2.902), p<0.001	1.585 (1.092–2.301), p=0.015

Table S7: Baseline Characteristics of the Internal (MIMIC-IV) and External Validation Cohorts

Characteristic	Internal Cohort (MIMIC-IV)	External Validation Cohort	P value
Demographics			
Age (years), mean ± SD	68.8 ± 14.6	70.2 ± 13.7	0.141
Male, n (%)	802 (61.0%)	169 (61.2%)	0.952
BMI (kg/m²), mean ± SD	29.2 ± 7.44	29.1 ± 7.15	0.837
Comorbidities, n (%)			
Hypertension	313 (23.8%)	71 (25.7%)	0.497
Acute kidney injury (AKI)	912 (69.4%)	179 (64.9%)	0.142
Chronic kidney disease (CKD)	427 (32.5%)	97 (35.1%)	0.395
Diabetes mellitus (DM)	485 (36.9%)	122 (44.2%)	0.023

Characteristic	Internal Cohort (MIMIC-IV)	External Validation Cohort	P value
Hyperlipidemia (HLD)	594 (45.2%)	129 (46.7%)	0.647
Heart failure (HF)	1024 (77.9%)	220 (79.7%)	0.507
Myocardial infarction (MI)	459 (34.9%)	95 (34.4%)	0.875
Ischemic heart disease (IHD)	898 (68.3%)	196 (71.0%)	0.376
Chronic obstructive pulmonary disease (COPD)	240 (18.3%)	51 (18.5%)	0.934
Disease severity scores, mean ± SD			
SOFA	7.70 ± 3.62	7.64 ± 3.64	0.802
APACHE II	22.9 ± 7.50	22.9 ± 7.38	0.965
SAPS II	46.3 ± 14.5	46.3 ± 14.8	0.985
APS III	59.3 ± 21.9	59.4 ± 21.5	0.938
OASIS	36.2 ± 8.94	36.0 ± 8.93	0.749
Charlson Comorbidity Index (CCI)	6.28 ± 2.82	6.57 ± 2.71	0.117
Laboratory parameters, mean ± SD			
Red cell distribution width (RDW, %)	15.4 ± 2.54	15.1 ± 2.38	0.069
Albumin (g/dL)	3.08 ± 0.59	3.04 ± 0.52	0.285
RAR	5.23 ± 1.61	5.04 ± 1.45	0.064
Lactate (mmol/L)	3.11 ± 2.58	3.15 ± 2.41	0.812
Creatinine (mg/dL)	1.97 ± 1.64	1.95 ± 1.43	0.846
Blood urea nitrogen (BUN, mg/dL)	37.9 ± 25.4	37.8 ± 25.2	0.950
Hemoglobin (g/dL)	11.1 ± 2.46	10.9 ± 2.28	0.209

Characteristic	Internal Cohort (MIMIC-IV)	External Validation Cohort	P value
Platelet count (×10³/μL)	207 ± 102	196 ± 99	0.097
Treatments and interventions, n (%)			
Vasopressor use	1292 (98.3%)	267 (96.7%)	0.092
Mechanical ventilation	1198 (91.2%)	253 (91.7%)	0.796
Continuous renal replacement therapy (CRRT)	276 (21.0%)	37 (13.4%)	0.003
Outcomes			
28-day ICU mortality, n (%)	444 (33.8%)	89 (32.2%)	0.618

Abbreviations: SD, standard deviation; BMI, body mass index; AKI, acute kidney injury; CKD, chronic kidney disease; DM, diabetes mellitus; HLD, hyperlipidemia; HF, heart failure; MI, myocardial infarction; IHD, ischemic heart disease; COPD, chronic obstructive pulmonary disease; SOFA, Sequential Organ Failure Assessment; APACHE II, Acute Physiology and Chronic Health Evaluation II; SAPS II, Simplified Acute Physiology Score II; APS III, Acute Physiology Score III; OASIS, Oxford Acute Severity of Illness Score; CCI, Charlson Comorbidity Index; RDW, red cell distribution width; RAR, red cell distribution width-to-albumin ratio; BUN, blood urea nitrogen; CRRT, continuous renal replacement therapy; ICU, intensive care unit. Note: P values were calculated using t-test or Mann-Whitney U test for continuous variables and chi-square test or Fisher's exact test for categorical variables, as appropriate. Statistically significant differences ($P < 0.05$) are highlighted in bold.
